# A score of DNA damage repair pathway with the predictive ability for chemotherapy and immunotherapy is strongly associated with immune signaling pathway in pan-cancer

**DOI:** 10.3389/fimmu.2022.943090

**Published:** 2022-08-23

**Authors:** Ke Ding, Youhua He, Jinfen Wei, Shuying Fu, Jiajian Wang, Zixi Chen, Haibo Zhang, Yimo Qu, Keying Liang, Xiaocheng Gong, Li Qiu, Dong Chen, Botao Xiao, Hongli Du

**Affiliations:** ^1^ School of Biology and Biological Engineering, South China University of Technology, Guangzhou, China; ^2^ College of Life Science, Zhaoqing University, Zhaoqing, China; ^3^ Clinical Laboratory Department of Longgang District People’s Hospital of Shenzhen & The Second Affiliated Hospital of the Chinese University of Hong Kong, Shenzhen, China; ^4^ Fangrui Institute of Innovative Drugs, South China University of Technology, Guangzhou, China

**Keywords:** DNA damage repair (DDR), therapeutic sensitivity, pan-cancer, genomic instability, tumor microenvironment

## Abstract

DNA damage repair (DDR) is critical in maintaining normal cellular function and genome integrity and is associated with cancer risk, progression, and therapeutic response. However, there is still a lack of a thorough understanding of the effects of DDR genes’ expression level in cancer progression and therapeutic resistance. Therefore, we defined a tumor-related DDR score (TR-DDR score), utilizing the expression levels of 20 genes, to quantify the tumor signature of DNA damage repair pathways in tumors and explore the possible function and mechanism for the score among different cancers. The TR-DDR score has remarkably predictive power for tumor tissues. It is a more accurate indicator for the response of chemotherapy or immunotherapy combined with the tumor-infiltrating lymphocyte (TIL) and G2M checkpoint score than the pre-existing predictors (CD8 or PD-L1). This study points out that the TR-DDR score generally has positive correlations with patients of advanced-stage, genome-instability, and cell proliferation signature, while negative correlations with inflammatory response, apoptosis, and p53 pathway signature. In the context of tumor immune response, the TR-DDR score strongly positively correlates with the number of T cells (CD4+ activated memory cells, CD8+ cells, T regs, Tfh) and macrophages M1 polarization. In addition, by difference analysis and correlation analysis, *COL2A1*, *MAGEA4*, *FCRL4*, and *ZIC1* are screened out as the potential modulating factors for the TR-DDR score. In summary, we light on a new biomarker for DNA damage repair pathways and explore its possible mechanism to guide therapeutic strategies and drug response prediction.

## Introduction

DNA damage repair (DDR) plays an essential role in maintaining the normal function of cells and genome integrity. When DNA damage occurs, Base Excision Repair (BER), Nucleotide Excision Repair (NER), and the Direct Repair (DR) pathways are activated to help with repairing DNA base damage. At the same time, Mismatch Repair (MMR) contributes to correcting base mispairs and small loops. Homology-Dependent Recombination (HDR), Non-Homologous End Joining (NHEJ), Fanconi Anemia (FA), and Translesion Synthesis (TLS) pathways are, alone or together, involved in the repair of DNA strand breaks and complex events like interstrand crosslinks (1, 2).

The functional abnormalities of DNA damage repair (DDR) affect cancer risk, progression, and therapeutic effect (3–5). DDR deficiencies, in many cancers, have built a well-established connection with cancer development through analyses of specific pathways loss (6) or single-gene mutations (7, 8) like *TP53 (* 3, 9–11). Meanwhile, DDR deficiencies can activate the innate immune system by up-regulating the STING pathway (12). Furthermore, the defect of MMR genes results in the accumulation of mutations and the production of neoantigens, enhancing the anti-cancer immune response (13, 14). In conclusion, alterations of DNA damage repair genes produce multifaceted effects on cancer patients by allowing genomic instability (15, 16), arousing immune responses (17, 18), and changing the tumor microenvironment from multiple aspects (19, 20).

Previous studies have suggested that DDR pathways are associated with sensitivity to chemotherapy, immunotherapies, and radiotherapy (21–23). Most chemotherapy regimens contain agents that directly induce DNA damage triggering cell apoptosis, such as anthracyclines and alkylating agents, while radiotherapy can also cause DNA damage to uncomplicated locoregional tumors (24). It is reported that alteration of some DDR genes in cancers, including *BRCA1*, *BRCA2*, *RAD51B*, and *RAD51C*, is associated with therapy sensitivity (25–29). For immunotherapies, tumors with abnormal DDR function tend to accumulate tumor-specific neoantigens, which result in a strong anti-tumor immune response (30, 31). For prior studies provide interesting insights between DDR genes and therapy sensitivity, some assays have been developed and validated to detect DDR pathway (such as HDR or FA) function in tumors as a predictor of response and prognosis after chemotherapy, radiotherapy, or with adjuvant settings (23, 32, 33). However, the primary mechanism driving DDR function abnormal remains unknown on a pan-cancer scale and it is incomplete to understand how DDR genes affect the cancer treatment effect of chemotherapy and immunotherapy.

Since the DNA repair mechanisms are essential for preventing tumor formation (34), there is still a lack of a comprehensive study of gene expression of the DDR pathway and their association with cancer progression and resistance to therapy. To fill this gap, we defined the DNA damage repair tumor score using a 20-gene signature to create a pan-cancer quantification and explore the signature’s function and significance in great depth. This is the first study to identify gene expression signatures of DNA damage repair that reflect the efficacy of treatments, which can be easily applied to a multitude of patient samples. Our study strongly suggests that a high tumor DDR score is associated with elevated proliferation and decreased inflammatory response. Furthermore, it provides the theoretical basis for understanding the critical roles of DDR level alteration, which lights on a framework to guide therapeutic strategies.

## Materials and methods

### Multi-omics data and clinical data collection

We downloaded available level-3 molecular data, including mRNA expression data, copy number alterations (CNAs) data, genomic somatic mutation (SNAs) data, and clinical information across 11 cancer types with normal sample numbers greater than 30 and tumor sample numbers greater than 100 from the Cancer Genome Atlas (TCGA) data portal (35). The chemotherapy treatment information of six cancer types used in this study, including therapy types, drug names, and response measures, was extracted from the TCGA dataset. The immunotherapy dataset consists of the GSE78220 dataset from the National Center for Biotechnology Information Gene Expression Omnibus (NCBI GEO) (36, 37) and the other four datasets of previous studies (38–41). As the chemotherapy dataset from TCGA only included data from untreated samples, we accordingly screened out samples containing pre-treatment transcriptome data and post-treatment clinical response information (42) from the immunotherapy dataset.

For genomic instability, we obtained data of the samples from 11 cancer cohorts in TCGA, including mutation counts, fraction genome altered, and MSI sensor score through the cBioPortal website (43). Single-nucleotide variant neoantigens and indel neoantigens were calculated by Thorsson et al. (44, 45).

### Cancer cell lines data collection and preprocessing

We collected mRNA expression data of cell lines from the Cancer Cell Line Encyclopedia (CCLE) (46). The drug response data was collected from Genomics of Drug Sensitivity in Cancer (GDSC), including 153 drugs and 1016 cell lines (47). We used half-maximal inhibitory concentration (IC_50_) values to distinguish drug-resistant and drug-sensitive cell lines. For the specific drug, we defined the drug-resistant cell line as the cell line whose IC_50_ value was greater than the mean value plus 0.3 times the SD of all cell lines (48). Otherwise, the drug-sensitive cell line was defined as the cell line with IC_50_ less than the mean value minus 0.3 times the SD.

### DDR genes’ copy number alterations, genomic somatic mutation, and mRNA expression analysis in cancers patients

The proportions of CNAs and SNAs for 71 DDR genes were calculated among tumor samples in 11 cancer types. The copy number segmentation data (SCNA score) was calculated by the Circular Binary Segmentation (CBS) algorithm, and the Genomic Identification of Significant Targets in Cancer calls was calculated using GISTIC2.0, comprising -2 (deletion), -1 (loss), 0 (diploid), 1 (gain) and 2 (amplification) (44, 49). Gene Set Enrichment Analysis (GSEA) interpreted the association between CNAs and mRNA expression of a gene (50). The R-package “DESeq2” was used to assess DEGs between tumor and normal tissue. Genes with |Log2FC| > 1 and FDR < 0.01 were considered as DEGs.

### Tumor-related DNA damage repair score calculation and classification

Focusing on expression dysregulation of the 71 core DNA damage repair pathway-specific genes (6). We chose 20 genes whose expressions were up-regulated between tumor and normal tissue in more than four cancer types and had an average TPM higher than 1. We termed these DDR genes (*POLQ*, *BRIP1*, *FANCA*, *XRCC2*, *EXO1*, *EME1*, *BLM*, *BRCA2*, *RAD51*, *SHFM1*, *BRCA1*, *UBE2T*, *SLX1A*, *FANCB*, *FANCD2*, *FEN1*, *ERCC3*, *GEN1*, and *PRKDC*) as “Tumor-related core DDR gene set”. Because of their importance in cancer, we used Gene Set Variation Analysis (GSVA) to calculate the TR-DDR score based on this core DDR gene set for each sample (51). We respectively calculated the TR-DDR score across all cancer samples to obtain the difference among 11 cancer types and in each cancer type to classify samples into high-score and low-score groups. The score distribution was shown in **Figure S4** for each cancer. Patients were divided into two groups (high-score and low-score) using the median value as a threshold for each cancer type.

### Cancer cell lines data collection and preprocessing for gene set enrichment analysis

The single-sample gene set enrichment score was calculated using the GSVA program to derive absolute enrichment scores of gene sets from previously experimentally validated gene signatures from several publications or MsigDB. These signatures include tumor proliferation signature, tumor inflammation signature, DNA replication, G2M checkpoint, Stem cell signature, MYC targets, EMT markers, collagen formation, P53 pathways, apoptosis, degradation of ECM, angiogenesis, inflammatory response, ECM related genes, TGFB signaling, genes up-regulated by ROS, radiosensitivity index, PI3K/AKT/mTOR pathway and hypoxia signature (52–54) (**Table S4**). The RNA expression counts matrix was used as an input to calculate the GSVA score of each signature for each sample in RNA-seq mode. Spearman correlation between TR-DDR score and scores of differentially enriched gene sets were calculated in 11 cancer types and CCLE cell lines. Only correlation with p<0.05 were demonstrated in the heatmap.

### Immune cells proportion and tumor-infiltrating lymphocyte Z score

The estimated proportion of individual 22 immune cell types was obtained using CIVERSORT (55). For a given sample, we computed the Spearman correlation coefficient between TR-DDR score and relative abundance of each immune cell type in 11 cancer types and visualized the correlation with p<0.05 in the heatmap. In CIBERSORT, LM22 (22 immune cell types) for signature gene file, 100 for permutations, and disabled quantile normalization for all runs were selected.

The comprehensive TIL score for each sample was calculated by applying an algorithmically optimized method, which used the expression of representative genes or gene sets of single samples from 20 single factors and six immune cell types. We conducted the calculation using the available R code developed by Charoentong et al. (56). RNA expression matrix transformed into log2(TPM+1) values were used as the input, and the average Z score in the output file was selected as TIL comprehensive score.

### Identification of pathways alterations between TR-DDR score high and low tumors

The difference in gene expression between TR-DDR score high and -low groups was analyzed using the R-package “DESeq2”. Genes with |Log2FC| > 2 and FDR < 0.01were considered as significantly differentially expressed. We choose the genes co-upregulated in at least three cancer types as DDR positively related genes. R-package “cluster profile” was used to process the Gene Ontology (GO) term and Kyoto Encyclopedia of Genes and Genomes (KEGG) pathway enrichment analysis.

### Statistical analysis

R-package “pROC” was used to plot the receiver operator characteristic (ROC) curve. Spearman rank correlation analysis was applied to estimate the statistical significance between TR-DDR score and other continuous variables, including gene set enrichment score, immune cells proportion, and genomic instability. Wilcoxon rank-sum test was used to obtain the significance of differences between continuous values, while Pearson’s Chi-square test and Fisher’s exact test were used on categorical variables.

## Results

### A systematic analysis revealing common dysregulation of DDR genes in human cancers

**Figure 1:**
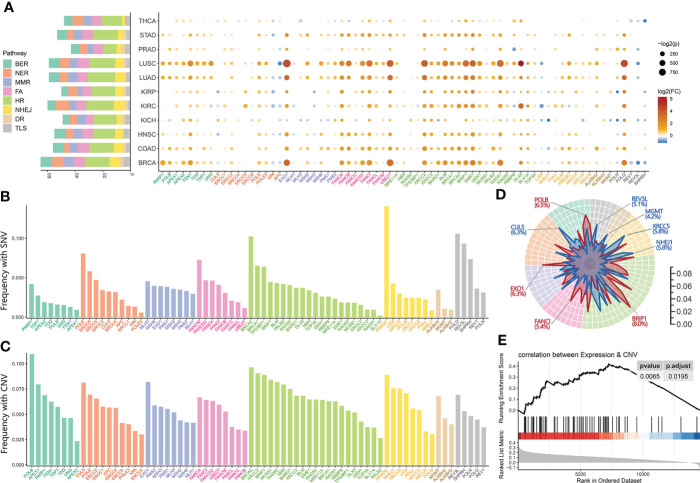
Overview of core DDR genes dysregulation across pan-cancer. **(A)** The bar chart (left) indicates the total number of DDR genes identified as differentially expressed genes (DEGs) between neoplastic and normal tissue, and the dot plots (right) respectively show the fold change (dot’s color) and p-Value (dot’s size) of 71 core DDR genes across eight DDR pathways in 11 cancer types. **(B, C)** The pan-cancer prevalence of somatic single-nucleotide variants (SNVs) **(B)** and copy number alterations (CNAs) **(C)** events in the 71 core DDR genes in the 11 TCGA cancer tumors. **(D)** Radar plot of the prevalence of copy number gain (red line) and loss (blue line) events. The most prevalent gene (the gene belonged to copy number gain uses red color while the loss uses blue) in each DDR pathway is marked with the pan-cancer prevalence. **(E)** The Gene Set Enrichment Analysis (GSEA) of DDR pathways on genes ranked based on their correlation between expression and copy number alterations in the pan-cancer tumors. Both **(A–D)** share the same color bar representing different DDR pathways.

We scrutinized the expression alterations of 71 core genes involved in DNA damage repair pathways, including Base Excision Repair (BER), Nucleotide Excision Repair (NER), Direct Repair (DR), Mismatch Repair (MMR), Homology-Dependent Recombination (HDR), Non-homologous End Joining (NHEJ), Fanconi Anemia (FA) and Translesion Synthesis (TLS) (6) (**Table S1**), and observed recurrent up-regulation of core DDR genes expression, relative to normal controls, among the 6166 The Cancer Genome Atlas (TCGA) cancer samples across 11 tumor types (**Table S2**). Among the eight DDR pathways, the HDR and BER pathways responsible for the double-strand break (DSB) and the single-strand break (SSB) restoration, as well as the FA pathway were significantly up-regulated across the pan-cancer cohort (**Figure 1A**), which meant at least two genes of the pathway were up-regulated expression in all cancer types.

On the individual gene level, the majority of core DDR genes were identified as differentially expressed genes (DEGs) (FDR < 0.05) between tumor and normal tissue. Six cancers owned more than 50 DEGs of DDR, including BRCA (65 DEGs), LUAD (59 DEGs), LUSC (59 DEGs), COAD (56 DEGs), HNSC (55 DEGs), and STAD (53 DEGs), presented in **Figure 1A** (**Table S3**). We also observed that all cancer types harbored similar change patterns of DDR gene expression levels. Among these DDR DEGs, there were 7 DDR genes (*POLQ*, *BRIP1*, *FANCA*, *XRCC2*, *EXO1*, and *EME1*) identified in nine or more cancer types and 13 DDR genes (*BLM*, *BRCA2*, *RAD51*, *SHFM1*, *BRCA1*, *UBE2T*, *SLX1A*, *FANCB*, *FANCD2*, *FEN1*, *ERCC3*, *GEN1*, and *PRKDC*) were found in four to nine kinds of cancers. These 20 DDR-DEGs had significantly increased expression levels in cancers, so we termed these genes a “Tumor-related core DDR gene set” (**Table S1**).

In addition, the mutations and copy number alterations of core DDR genes were also observed across 11 major cancer types (**Figures 1B, C**). NER and TLS pathways responsible for the bulky adducts restoration, as well as HDR pathway repairing DNA double-strand breaks, had significantly high mutation frequencies which meant the pathway contained over two mutated genes with more than 5% of samples. On the individual gene level, *PRKDC* belonged to the NHEJ pathway, as the DDR gene with the most prevalent mutation (14.1% in pan-cancer) was one of the top 2 DDR genes ranked by mutation frequency in 10 kinds of cancers including COAD (23.68%), STAD (22.71%), LIHC (14.94%), and LUAD (14.49%) (**Figure S1**). We also observed significant regions alteration of all DDR pathways except DR when analyzed copy number data across the pan-cancer cohort, using identical filter conditions with mutation. Under the same criteria, the HDR, NHEJ, BER, and FA pathways exhibited significant amplification, while only NHEJ and HDR pathways showed significant deletion (**Figure 1D**). The most frequently amplified genes across the pan-cancer atlas were *BRIP1* (HDR, 8.04%), *POLB* (BER, 6.54%), *MUS81* (HDR, 6.42%), *NBN* (HDR, 6.31%), and *EXO1* (MMR, 6.26%), while the most frequently deleted genes were *BRCA2* (HDR, 6.44%), *XRCC2* (HDR, 6.38%), and *CUL5* (NER, 6,28%). The amplified DDR pathways were noticeably similar to the overexpression DDR pathways, so we investigated the correlation between DNA copy number and mRNA expression in pan-cancer samples. The results showed the overexpression of the DDR genes might be driven by copy number amplification in the tumor tissues (**Figure 1E**).

### Neoplasm discrimination by a DNA damage repair gene expression signature

**Figure 2:**
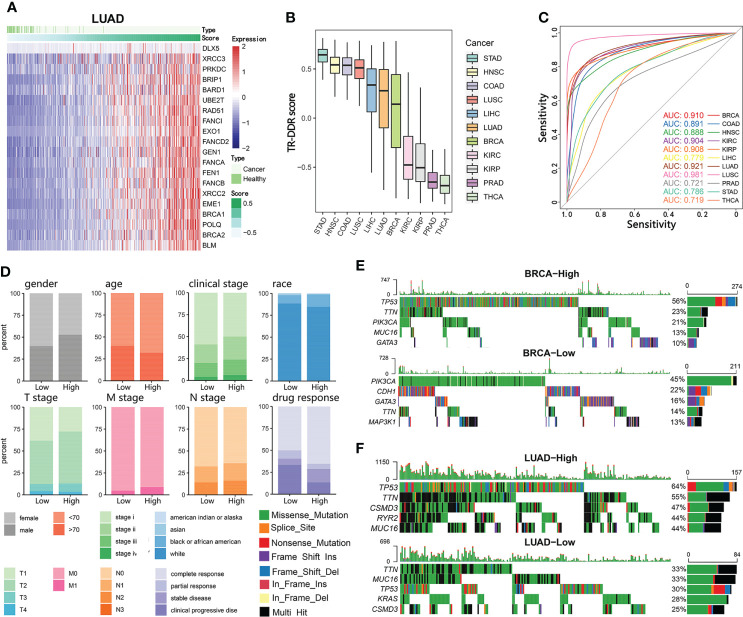
Identification of a DNA damage repair gene expression signature and its association with clinical outcomes. **(A)** Samples are ordered from lowest to highest Tumor-related DNA damage repair (TR-DDR) scores with 20-gene expression distribution in LUAD. The top color bar shows the samples’ type and TR-DDR score. **(B)** TR-DDR scores are calculated based on the mRNA abundance signature in 11 tumor types, sorted by the median GSVA score for each cancer type. **(C)** ROC curves for the performance of TR-DDR score for neoplasm discrimination for 11 cancer types from TCGA cohorts. Area Under the Curve (AUC) values are presented in the figures and **Table 1**. **(D)** Comparison of conventional clinical parameters between TR-DDR high- and low-group patients in LUAD. **(E, F)** Top 5 frequently mutated genes in TR-DDR score high-group (top) and low-group (bottom) in BRCA **(E)** and LUAD **(F)** cohort. Genes are ranked by their mutation frequency in patients.

Because most of the DDR genes with an altered expression between tumor and normal tissue belonged to the “Tumor-related core DDR gene set”, we used the expression profile of the 20 core genes to obtain a tumor-related DDR score (TR-DDR score) by Gene Set Variation Analysis (GSVA). Examining whether the TR-DDR score was only affected by a small group of key genes, we found the contribution of individual genes to the score was approximately uniform (**Figure 2A**). The sample’s TR-DDR score was calculated across all cancer types (**Figure 2B**) and in each cancer type (**Figure S2**), respectively. The TR-DDR score greatly varied among different cancer types. Some cancers had high scores such as STAD, HNSC, COAD, and LUSC, while others had low scores such as THCA, PRAD, KIRP, and KIRC (**Figure 2B**).

Compared with normal tissues, the TR-DDR score was significantly higher in tumor tissues across all cancer types (**Figure S2**). The distribution of the normal sample significantly concentrated on the low-scored regions (**Figure 2A** and **Figure S3**). Hence, we evaluated whether the TR-DDR score would be a tumor predictive marker for all cancer types, and the receiver operating characteristic (ROC) curve was used to measure the true-positive rates against the false-positive rates at various thresholds of the TR-DDR score. The area under the ROC curve (AUC), representing the predictive power, ranged from 0.730 to 0.988 across 11 cancer types (**Figure 2C** and **Table 1**) and the results showed that the score had a strong predictive power to distinguish between tumor and normal tissue. Among them, an excellent predictive value having AUC higher than 0.9 was found in 6 cancers (LUSC, LUAD, KIRP, KIPC, BRCA, and COAD). What is more, we observed that cancer types sharing similar tissue origins or carcinogenic risk factors harbor similar AUC values, such as seen in LUSC (0.988) and LUAD (0.946), KIRP (0.930) and KIRC (0.927). These results demonstrate the TR-DDR score is altered in tumors and could serve as a potential indicator for tumor formation with substantial biological significance.

**Table 1:** The sample size statistics and AUC value of TR-DDR score for neoplasm discrimination.

Cancer Type	No. of Normal Tissues	No. of Cancer Tissues	AUC Value of TR-DDR
LUSC	49	496	0.988
LUAD	58	512	0.946
KIRP	32	288	0.930
KIRC	72	527	0.927
BRCA	99	1078	0.925
COAD	41	453	0.902
HNSC	44	497	0.897
LIHC	50	371	0.798
STAD	32	373	0.796
THCA	56	505	0.735
PRAD	51	482	0.730

To investigate whether the TR-DDR score was clinically relevant, we divided patients into two groups depending on the TR-DDR score in each dataset: high-score groups (the 50% samples at the top) and low-score groups (the 50% samples at the bottom). We observed that the high-score group had a higher proportion of patients classified pathologically as advanced-stage (tumor stage iii and iv) (**Figure 2D** and **Figure S5**).

We next assessed the correlation between the TR-DDR score and the somatic single-nucleotide variants (SNVs) features. After analyzing the SNVs features of two sample groups across 11 cancer types, we observed a strong correlation between the score and the SNV frequency (**Figure S6**). In the high-score group, the top 5 genes showed a significantly higher mutation rate than those in the low-score group, such as the *TTN* gene. Additionally, the point mutation rate of *TP53* had an extreme elevation in high-score groups compared to low-score groups among BRCA, COAD, LIHC, LUAD, LUSC, PRAD, and STAD (**Figures 2E, F** and **Figure S7**).

### The correlation between the key cancer hallmarks and TR-DDR score

**Figure 3:**
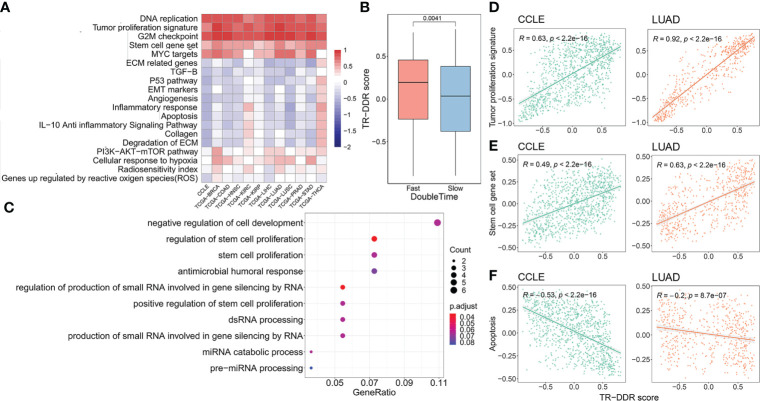
Association of TR-DDR score with cancer hallmarks and pathway signatures. **(A)** Heatmap showing the Pearson’s correlation coefficient for GSVA scores of cancer hallmarks signatures and TR-DDR score in tumors. Only the correlation coefficient with a p-Value less than 0.05 is shown in the heatmap. **(B)** The distribution of TR-DDR score of cancer cell lines with fast and slow proliferation rates is categorized according to the double-time of the cell lines. A two-sided Student’s t-test was used to assess the difference. p < 0.05. **(C)** GO term enrichment in the differentially expressed genes between high- and low- groups of TR-DDR score. **(D–F)** Spearman correlation between TR-DDR score and stem cell gene set, tumor proliferation signature, apoptosis score of tumor cells and tissues in CCLE and LUAD. p < 0.05.

To reveal the relationship between the cancer hallmarks and TR-DDR score in each cancer type, we calculated the signature enrichment score of each cancer hallmark (**Table S4**) by GSVA analysis, using the publicly available gene set in Molecular Signatures Database (MSigDB). And then the Pearson’s correlation coefficient R and corresponding P-Value were computed between the TR-DDR score and enrichment score of each cancer hallmark with independent signatures. Determining more reliable and consistent results of the correlation across multiple cancers, data from the Cancer Cell Line Encyclopedia database (CCLE) was included in the analysis. The heatmap of the correlations showed that DNA replication, Tumor proliferation signature, G2M checkpoint, Cell stemness signature, and MYC targets gene signatures had a strong positive correlation with TR-DDR score across 11 TCGA cancer types (mean correlation coefficient r > 0.53) and CCLE database (correlation coefficient r > 0.49), whereas ECM related genes signature, Angiogenesis, TGFB signature, Apoptosis, and p53 pathway signature had a negative correlation across 7–10 TCGA cancer types (mean correlation coefficient r < -0.14) and CCLE database (correlation coefficient r < -0.39) (**Figure 3A** and **Table S5-1**). It is worth mentioning that the coefficient R between Tumor cell proliferation signature score and TR-DDR score was relatively high in all cancer types (r = 0.75 – 0.92 in TCGA, r = 0.63 in CCLE), implying a strong correlation between tumor proliferation and TR-DDR score (**Figure 3D**). Using the data from the CCLE database for further analysis, we also observed that the group of cancer cell lines with fast growth (the cell lines with short 30% of the double-time) own a higher TR-DDR score than the group with slow growth (the cell lines with long 30% of the double-time), which verified the above results (**Figure 3B**). In addition, TR-DDR score had a significantly positive correlation with the Cell stemness signature score (r = 0.38 – 0.69 in TCGA, r = 0.49 in CCLE) and had a negative correlation with the Apoptosis signature score (r = -0.16 – -0.58 in TCGA, r = 0.53 in CCLE) (**Figures 3E, F**). These results were highly consistent among the different types of cancer tissue and cell line, implying indicative value across pan-cancer.

After identifying the DEGs (FDR < 0.05 and |log2FC| > 2) between high- and low-TR-DDR score groups in 11 cancer types respectively (**Table S6**), we screened a total of 75 DEGs (69 up-regulated and six downregulated genes) by choosing the DEGs overlapping in more than three cancers (**Table S7**). 11 GO terms were uncovered, and these terms contained three stem cell related terms and one cell development related term, including “regulation of stem cell proliferation”, “positive regulation of stem cell proliferation”, “stem cell proliferation” and “negative regulation of cell development” (**Figure 3C**). The results of GO analysis were consistent with the results of cancer hallmarks above.

### The predictive capability of score combining TR-DDR, TIL and G2M checkpoint for response to chemotherapy or immunotherapyacross multiple cancers

According to the results above, the TR-DDR score showed a positive correlation with tumor proliferation level and a negative correlation with the inflammatory response. Besides, previous studies have demonstrated that functions of DDR-related genes influence chemotherapy resistance leading to poor patient survival (57, 58). Therefore, we further speculated that the TR-DDR score could serve as a predictor of response to chemotherapy or immunotherapy.

**Figure 4:**
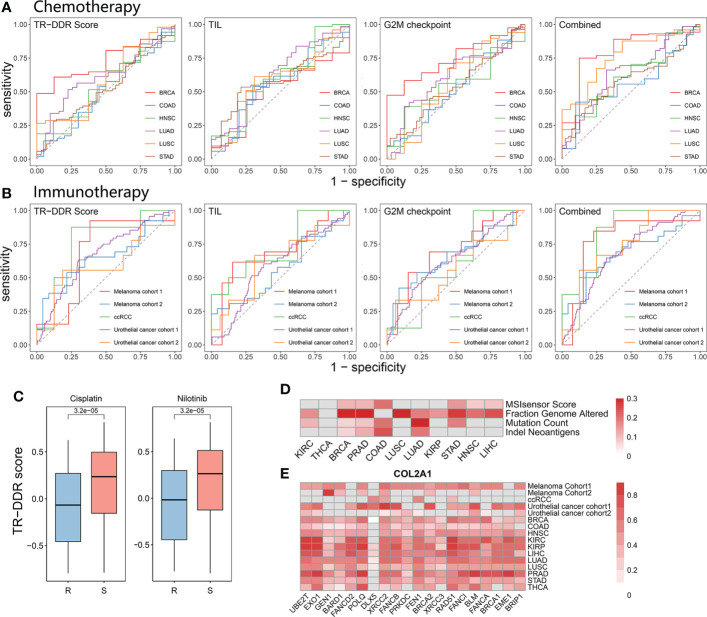
Predictive ability of a combination of the TR-DDR score and TIL score in clinical response of chemotherapy and immunotherapy across pan-cancer types. **(A, B)** ROC curves for the performance of TR-DDR score, TIL score, G2M checkpoint score, and the combination of both scores for predicting chemotherapy **(A)** and immunotherapy **(B)** response in patients. Area Under the Curve (AUC) values are presented in **Table 3**. **(C)** The distribution of TR-DDR score of cancer cell lines under resistant and sensitive conditions with Cisplatin and Nilotinib medication therapy. A two-sided Student’s t-test was used to assess the difference. p < 0.05. **(D)** Heatmap showing the Pearson’s correlation coefficient for genomic instability with TR-DDR score in tumors. **(E)** The Pearson’s correlation coefficient for *COL2A1* (the most correlated DEGs with TR-DDR score) and core-DDR genes. Only the correlation coefficient with a p-Value less than 0.05 is shown in the heatmap.

To verify this hypothesis, we first downloaded the IC_50_ data of 153 anti-cancer drugs in corresponding cancer cell lines from the CCLE database and grouped the cell lines as sensitivity groups or resistance groups for different agents respectively. Analyzing the correlation between TR-DDR score and drug sensitivity, we observed that cell lines in sensitivity groups showed relatively high levels of TR-DDR score in 128 drugs (83.7%), and it is in none of the drugs that the resistance group was correlated with high levels of TR-DDR score (**Table 2**). The TR-DDR score of sensitivity and resistance groups for the drug Cisplatin, Nilotinib, and others were shown in **Figure 4C** and **Table S8**.

**Table 2:** The percentage of sensitivity or resistance cell line groups having a high level of TR-DDR score.

The group with a high TR-DDR score	No. of drug agents	The percentage (%)
Sensitivity group	128	83.7
Resistance group	0	0
No significance	153	16.3

Moreover, we focused on clinical chemotherapy and immunotherapy by analyzing the datasets from TCGA treated with chemotherapy and five published GEO datasets on PD-L1/PD-1 blockade immunotherapy (**Table S2**) (59–63). The pre-treatment transcriptome information and post-treatment clinical response data were downloaded for the subsequent computation of predicting clinical response to the drug. The ROC curve and AUC were used to evaluate the capability of the TR-DDR score to be a predictor of response to chemotherapy or immunotherapy. The results showed that the AUC values were 0.432–0.7 (mean AUC = 0.557) in chemotherapeutic datasets (**Figure 4A**) and were 0.454–0.680 in immunotherapeutic treatment datasets (mean AUC = 0.594) (**Figure 4B**). We observed the predictive power of the TR-DDR score was not significantly higher than the pre-existing predictors, like the tumor-infiltrating lymphocytes (TILs), as well as numerous factors that independently predict clinical response, including *PDL1* expression, immune cell exhaustion, and disordered expression levels of cytokines (60, 61).

As the TILs score is usually used as a predictor for response to chemotherapy or immunotherapy (64–67), and coordinated activity of G2M checkpoint is also induced by DNA damage (68). So, we combined the TR-DDR score, the TIL score and G2M checkpoint score to optimize the predictive accuracy of drug response. The results showed that the AUC values of the combined score were 0.564–0.800 (mean AUC = 0.662) in chemotherapeutic datasets (**Figure 4A**) and 0.679–0.844 (mean AUC = 0.744) in immunotherapeutic treatment datasets (**Figure 4B**). The results showed that the combined score was more suitable for immunotherapy than chemotherapy. In this study, we did not focus only on pan-cancers but also on multiple therapeutic approaches and drugs. In chemotherapeutic datasets, the combined score has a better accuracy of predictive power in BRCA and LUSC, perhaps because of the multiple chemotherapeutic agents causing different toxic mechanisms in different cancer types. In addition to that, the results show that the combined score was more suitable for immunotherapy than chemotherapy, perhaps because tumor-infiltrating lymphocytes (TIL) is of critical importance in influencing immunotherapy (69, 70). Overall, the combination of the TR-DDR score, the TIL score and G2M checkpoint score has a higher AUC (mean AUC = 0.700) than the single TIL score (AUC = 0.505–0.686, mean AUC = 0.585) or G2M checkpoint score (AUC = 0.530-0.748, mean AUC = 0.610) alone for chemotherapy or immunotherapy, which suggests that this combined index exhibits higher accuracy to predict drug response (**Table 3**).

**Table 3:** The sample size statistics and AUC value of TR-DDR score, TIL score, G2M checkpoint score, and combined the two scores for the chemotherapy or immunotherapy research cohort.

Cancer Type	Therapy Type	No. of Responders	No. of Non-Responders	AUC Value			
				TR-DDR	TIL	G2M checkpoint	Combined score
BRCA	chemotherapy	156	8	0.700	0.505	0.748	0.800
LUSC	chemotherapy	49	16	0.624	0.587	0.568	0.761
LUAD	chemotherapy	62	31	0.590	0.618	0.608	0.640
HNSC	chemotherapy	64	8	0.432	0.602	0.547	0.619
STAD	chemotherapy	71	42	0.488	0.586	0.542	0.590
COAD	chemotherapy	52	20	0.508	0.564	0.530	0.564
ccRCC	immunotherapy	20	13	0.454	0.515	0.609	0.844
Melanoma cohort 1	immunotherapy	13	13	0.680	0.686	0.698	0.781
Urothelial cancer cohort 2	immunotherapy	9	16	0. 583	0.618	0.569	0.736
Urothelial cancer cohort 1	immunotherapy	68	230	0. 672	0.588	0.634	0.681
Melanoma cohort 2	immunotherapy	26	23	0.649	0.567	0.662	0.679

### Association of TR-DDR score with enhanced tumor immunogenicity, genome-instability, and expression of *COL2A1*, *MAGEA4*, *FCRL4*, *ZIC1*

To explore the mechanisms of drug resistance involved in DDR, we investigated the correlations between TR-DDR score and numerous factors that independently predict clinical response, including tumor mutation burden (TMB), neo-antigen genotype, and the expression of TR-DDR-related DEGs. The significant high levels of genomic instability, including neoantigens, MSI sensor, mutation count burden, and fraction genome altered, were observed in the high TR-DDR score group in 11 cancer types (**Figure 4D** and **Table S5-2**), which indicated tumor cells with high levels of TR-DDR score harbored more mutations and genomic rearrangements which would carry more neo-antigens. To be specific, the fraction of genome altered showed a significantly positive correlation with TR-DDR score among most cancer types (BRCA, LUSC, KIRC, KIRP, HNSC, LIHC, PRAD, LUAD, and STAD), while neoantigens had significantly high R values in 4 cancer types (BRCA, COAD, PRAD, and LUAD) (p <0.01). This result was consistent with those of previous research that the DDR pathways are associated with hypersensitivity to chemotherapeutic drugs (71). In addition to this, some recent evidence also showed that DDR pathways could change specific factors, such as TMB, which could affect immunotherapy response across different tumor types (72, 73).

We next explored the correlations between identified DEGs and TR-DDR genes using Spearman correlation analysis. As a result, seven DEGs correlated with more than 10 TR-DDR genes (r>0.3) in at least 10 cancers were regarded as candidate genes, including *COL2A1* (the R ranged from 0.39 to 0.60), *MAGEA4* (0.24–0.62), *FCRL4* (0.33–0.62), *ZIC1* (0.24–0.33), *LCN15* (0.20–0.41), *PRSS3* (0.24–0.35), *CSAG1* (0.31–0.38) (**Table S9**). What is more, the expression of *COL2A1* and *MAGEA4* especially had significantly positive correlations with 19 TR-DDR genes, which is an especially large number compared with other DEGs. Collectively, the data show that the expression of 4 DEGs (*COL2A1*, *MAGEA4*, *FCRL4*, *ZIC1*) are strongly associated with the TR-DDR score and may play a critical role in the DDR pathways (**Figure 4E** and **Figure S8**).

### The composition and abundance of immune cells among different TR-DDR score levels in the context of tumor immune microenvironment

**Figure 5:**
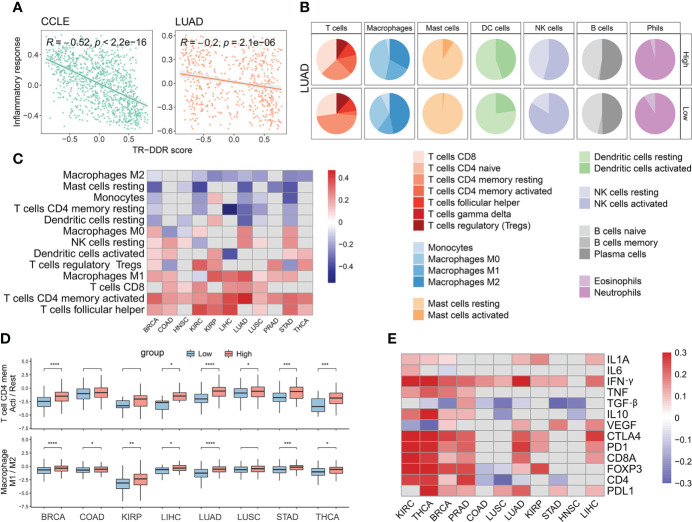
The abundance of immune cells and the expression of immune factors among high- and low- groups of TR-DDR score in 11 cancer types. **(A)** Spearman correlation of the TR-DDR score and Inflammatory response score of tumor cells and tissues in CCLE and LUAD. **(B)** The abundance difference of six main subclass immune cells in LUAD between high- and low- groups of TR-DDR score. **(C)** Heatmap showing the Pearson’s correlation coefficient for the proportion of 13 types of immune cells and TR-DDR score in tumors. Only the correlation coefficient with a p-Value less than 0.05 is shown in the heatmap. **(D)** The distribution of ratio of T cells CD4 memory activation/resting and macrophages M1/M2 polarization in 8 cancer kinds. A two-sided Student’s t-test was used to assess the difference. p < 0.05. **(E)** Heatmap showing the Pearson’s correlation coefficient for the expression of several immune-related characteristics with TR-DDR score in tumors. Only the correlation coefficient with a p-Value less than 0.05 is shown in the heatmap. *p < 0.05, **p < 0.01, ***p < 0.001 and ****p < 0.0001.

To further explore the DDR mechanisms of drug resistance, we next considered the potential effects of the TR-DDR score on the tumor microenvironment. It has been verified that the abundance of CD8+ T cells correlates with a better response to immunotherapies (74, 75). As inflammatory response signatures were negatively correlated with the TR-DDR score shown in the above result (r = -0.09 – -0.60) (**Figure 5A**), we sought to determine the differences in types and abundances of various immune cells with tumor samples of the high- or low-TR-DDR score. To apply machine learning-based CIBERSORT to classify and estimate the level of immune cell infiltration, we divided the 22 immune cells into seven categories: T cells, B cells, macrophages, dendritic cells, natural killer cells, mast cells, and granulocytes. The difference in abundance of seven types of immune cells in the high group and the low group of BRCA was shown that the high-score group contained a high level of many kinds of T lymphocytes and Macrophages M1 cells compared to the low-score group (**Figure 5B**). The results for other TCGA cancer cohorts were presented in **Supplementary Files** (see **Figure S9 and Table S10**).

We further observed a correlation between TR-DDR score and the composition of immune cells for several cancers. The infiltration level of CD4+ memory T cells activated, Macrophages M1, T cells follicular helper, CD8+ T cells, T cells regulatory, NK cells resting, and Dendritic cells activated had strongly positive correlations with TR-DDR score in at least five cancers, whereas the infiltration of CD4+ memory T cells resting, Macrophages M2, Mast cells resting, and Monocytes had negative correlations with TR-DDR score across in over five cancer types (**Figure 5C**). In addition, the ratio of CD4+ memory T cells activation/resting and macrophages M1/M2 polarization was significantly higher in the high TR-DDR score group than in the low TR-DDR group in 5 kinds of cancers (**Figure 5D**).

Among the T cells category, the infiltration level of CD4+ memory activated cells was positively correlated with TR-DDR score in 11 cancers significantly (r = 0.17 – 0.47), and the infiltration level of CD4+ memory resting cells was negatively correlated across six cancer types (r = -0.13 – -0.55) (**Table S5-3**). CD8+ T cells, mainly involved in killing tumor cells, also had a positive correlation with TR-DDR scores across six cancers, such as KIRC (r = 0.25), and LUAD (r = 0.25). Infiltration levels of T follicular helper cells, which mainly play a role in protective immunity and help B cells produce antibodies against foreign pathogens, had highly positive correlations with TR-DDR score in 8 cancers (r = 0.11 – 0.40) (**Table S5-3**). Infiltration levels of T regulatory cells (Tregs), a suppressor of immune cells, correlated inconsistently with TR-DDR score in different cancers (positive correlation in five cancer types, and negative correlation in two cancer types). These results revealed that the increased number of CD8+ and CD4+ T cells might be related to the advanced stage of patients. For macrophages, macrophages M0 are polarized into M1 classically activated macrophages (inflammatory macrophage) or M2 alternatively activated macrophages (anti-inflammatory macrophages) under different stimulation (76). The infiltration level of macrophages M1 increased significantly along with the TR-DDR score increased, while the infiltration level of macrophages M2 decreased along with the TR-DDR score increased.

### The expression patterns of immune-related genes in different levels of the TR-DDR score

Besides the different composition and abundance of lymphocytes in different TR-DDR score levels, immune genes, which had become one of the most important factors influencing tumor immune microenvironment, were taken into consideration to find out possible reasons for the above differences between the high- and low-TR-DDR group. We observed that the expression of genes related to activated immune cells (such as CD4+ T cells, CD8+ T cells, and NK cells) had significant increases in groups with high TR-DDR scores (**Table S5-4**). High-score groups exhibited higher expression of genes involved in stimulatory immune-related genes, such as chemokines (*IFNG*, *IL1A*, *etc.*), tumor necrosis factor receptor superfamily genes (*TNF*), and IFN response, and exhibited lower expression of *VEGF* (**Figure 5E**), compared to low-score groups. In addition, *IL1A*, *IL6*, and *TNF* up-regulated in high-group may play important roles in macrophage M1 polarization, while inhibitory cytokines (such as *IL10* and *TGF-*) secreted by M2-like macrophages exhibited a higher expression in low-score groups (**Figure 5E**). And then, we analyzed the expression of immune checkpoint genes, including *CD4*, *CD8A*, *CTLA4*, *FOXP3*, *PD1*, and *PDL1*, finding that *PDL1*, *PD1*, and *CTLA4* had significantly higher expression in high-score groups (**Figure 5E**). These results of expression analysis of immune genes could partially explain the above finding of the different composition and abundance of lymphocytes in different TR-DDR score groups.

Overall, we found that the TR-DDR score had positive correlations with the expression of *PDL1*, and *IFNG* in over ten cancers, suggesting that these signatures may play more roles in the crossroads of the DDR pathway and immune response (**Figure 5E**). Positive correlations were also observed between the TR-DDR score and expression of *PD1* and *CTLA4* in 8 cancer types. We further identified *CD4* displayed negative correlations in several TCGA cancers (LUSC, LUAD, STAD, and COAD), while positive correlations in other four cancer types (KIRC, THCA, BRCA, and PRAD) (**Figure 5E**). Worthy of note, the TR-DDR score was observed to have a positive correlation with all expressions of immunotherapy-related factors in KIRC and THCA, suggesting that DDR pathways may have a strong influence on immunotherapy-related factors in these two cancers.

## Discussion

For the first time, we have used expression profiling data of transcriptome from cancer patients and tumor cell lines at the bulk level, combined with clinical information, to elucidate the comprehensive and profound links between dysregulation of the DDR pathway with cell proliferation signaling, inflammatory responses, tumor immunogenicity, genomic instability, composition and abundance of immune cells, immune-related genes across pan-cancers. We also found DDR pathway scores have the predictive capability of response to chemotherapy or immunotherapy in pan-cancer.

It is observed that alterations in DNA damage repair (DDR) genes are prevalent in different cancers, including overexpression, somatic mutations, and significant enrichment for copy number amplification. Analysis of the genetic landscape of DDR genes in cancer will have important implications for tumor diagnosis, individualized therapy, and targeted drug use (7, 8, 77). Tumor cells are characterized by rapid clonal proliferation that differs from normal cells and is accompanied by higher genomic instability, such as defects in DDR genes (78, 79). Based on the function of DDR genes and pathways, it has been verified that many alterations of DDR genes may increase the number of specific mutation types and the overall mutational burden (80, 81). However, frequent overexpression of DDR genes, which may play critical roles in cancer occurrence and progression, has long been neglected in cancer studies. Here, we defined 20 DDR-DEGs with significantly increased expression as a “Tumor-related core DDR gene set”. The importance of these genes in genome maintenance has been verified by multiple studies (2, 82). In our study, the TR-DDR score reflects the expression level of tumor-related DDR genes. A high level of TR-DDR score in cancers seemingly represents a barrier to achieving uncontrolled cell growth. However, this barrier just cannot slow down the progression of the tumor (83). Interestingly, our results point out that cancers with a higher TR-DDR score grow faster than cancers with a lower score. Moreover, a higher TR-DDR score is correlated with greater genomic instability, including neoantigens, MSI sensor, mutation count burden, and fraction genome altered. This paradox could be explained by a dynamic continuum of cancer initiation and progression. DDR genes belong to tumor suppressor genes, and their loss of function is conducive to the occurrence and development of tumors (6), while it is also widely observed that alterations of copy number amplification or overexpression in DNA damage repair (DDR) genes are prevalent in different cancers (32, 84, 85).

During the initial stages of cancer initiation, dysregulation of the DDR can lead to genomic instability that promotes tumorigenesis (6). However, as normal cells evolve progressively to a neoplastic state, they acquire a succession of these hallmark capabilities, including sustaining proliferative signaling, evading growth suppressors, enabling replicative immortality and so on (86). With the progression of tumor malignancy, tumor cells characterized by rapid clonal proliferation would cause high DNA replication stress, which leads to DNA double-strand breaks, genomic instability and selective pressure for p53 mutations (9). Therefore, increased replication stress and endogenous DNA damage level need more expression of DDR genes to exercise their function. This has been verified in previous studies that oncogenic stress can cause the dysregulation of cell proliferation, leading to the activation of the DDR pathway (9, 87). This is also concordant with observations in the literature that alterations of copy number amplification or overexpression in DNA damage repair (DDR) genes are prevalent in different cancers (32, 84, 85). In this study, the TR-DDR score reflects the expression level of tumor-related DDR genes. Our results point out that cancers with a higher TR-DDR score grow faster than cancers with a lower score. Therefore, a higher TR-DDR score is correlated with high DNA replication stress which leads to genomic instability.

Another main contribution of this study is to point out the predictive capability of the TR-DDR score for the effect of chemotherapy or immunotherapy in specific cancer types, and to put forward a combined index consisting of the TR-DDR score, TIL score and G2M checkpoint score, which has a stronger predicting accuracy than the pre-existing predictors (*PDL1* expression or CD8+ T cell density) in immunotherapy. Some DNA damage repair pathways or genes have been considered as a potentially useful application of diagnostic biomarkers, which predicts the sensitivity of the agent. For instance, it has been reported that the aberrant expression of *BRCA1* impairs the DNA damage repair machinery in certain tumors, and therefore, its expression levels can be an effective biomarker of survival for non-small-cell lung cancer patients with cisplatin-based chemotherapy (88). For another example, defective HDR or FA pathway would be used to predict sensitized tumors that are prone to exhibit cellular hypersensitivity to chemotherapeutic drugs, such as alkylating and cisplatin (71). In comparison, coordinated activity of G2M checkpoint is induced by DNA damage to response prediction for chemotherapy and radiotherapy (68, 89). Apart from the explanation mentioned above, our research shows that the reason for the TR-DDR score being an indicator of response prediction for chemotherapy or immunotherapy resistance may mainly correlate with genomic instability and tumor immune microenvironment. Having a strong connection with genomic instability and a high level of mutation burden, the high TR-DDR score of tumors usually implies more “alien” peptides which means higher immunogenicity of the tumor (72, 73, 90). The neo-antigens in the tumor, presented by MHC molecules, can potentially be recognized by the endogenous T cell repertoire, causing increased T cell influx and a better overall response rate to therapeutics (14, 63, 91). A growing body of clinical observations indicates that aberrant expression of DNA damage repair pathways in human cancers is correlated with changes in genome stability or immune composition, influencing therapy response.

**Figure 6:**
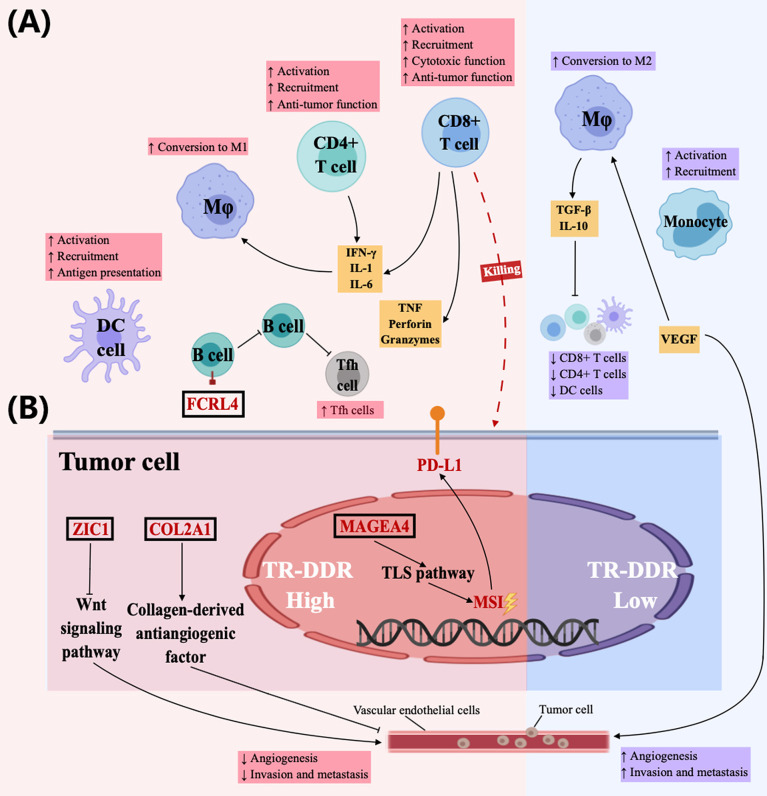
Possible mechanism underlying the difference in the efficacy of chemotherapy and immunotherapy in cancer patients with different TR-DDR scores. **(A)** The high-TR-DDR tumor microenvironment (TME) improves therapeutic effect through enhancing antitumor immunity of tumor-infiltrating lymphocytes (CD8+ T cells, CD4+ T cells), DCs, and M1-like macrophages, while low-TR-DDR tumor microenvironment has stronger ability of invasion and metastasis with an increasing number of Monocytes and M2-like macrophages. In high-TR-DDR tumors, CD8+ T cells exert cytotoxic effects through secreting TNF, perforin, and granzymes, and CD4+ T cells further activate other immune cells by secreting IL-1, IL-6, IFN-γ, and other cytokines, in which IL-1 and IL-6 play important roles to polarize macrophages toward M1 type with antitumor functions. Additionally, DCs promote tumor immunity through antigen processing and presentation. In contrast, VEGF-rich TME for low-TR-DDR tumors promotes M2 polarization of macrophages, which inhibits T cell (CD8+ T cells, CD4+ T cells) and antigen-presenting cells (DCs) function. In addition, recruitment and activation of Monocytes also could facilitate tumorigenesis by promoting immune suppression, angiogenesis, and tumor cell intravasation into the vasculature. **(B)** *COL2A1*, *MAGEA4*, *FCRL4*, and *ZIC1* possibly play roles in DDR function and immune microenvironment. *COL2A1* and *ZIC1* could suppress cancer metastasis by regulating cell signaling pathways and the Wnt signaling pathway respectively. *MAGEA4* exerts function in the TLS pathway, promoting genomic instability and tumor immunity. FCRL4+ B cell is capable of aborting B cell receptor-mediating signaling and proliferation, and producing TNF. The deep investigations of mechanisms of these DEGs at molecular levels have not been reported.

The tumor immune microenvironment, which is the key to influencing immunotherapy, appears heterogeneous across patients and tumor types. The heterogeneity of different immune cell interactions on immune activation can be observed between TR-DDR high- and low-score groups. Firstly, it has been documented that the aberrant DNA damage repair pathway has a strong correlation with genomic instability (92). Meanwhile, *MAGEA4*, one of the DEGs between TR-DDR high- and low-score groups, also promotes genome destabilization by contributing to TLS pathway activation, DNA-damage tolerance, and genome maintenance in cancers (93, 94). Other studies showed gene instability has an impact on the tumor microenvironment (90), including chemokines recruitment and activation of T lymphocytes, DCs, and NK cells (95–97). Among these immune cells, CD8+ T cells exert cytotoxic effects through secreting *TNF*, perforin, and granzymes (95), and CD4+ T cells further activate other immune cells by secreting IL-1, IL-6, IFN-γ, and other cytokines (96, 97), in which IL-1 and IL-6 play important roles to polarize macrophages toward M1 type with anti-tumor functions (98). In contrast, it has been confirmed that genomic stability tumors have more M2-like macrophages, which inhibit T cells and antigen-presenting cells by secreting TGF-β, and IL-10 (99, 100). In this study, the high-TR-DDR group contained high levels of not only genomic instability but also some immune cells (T lymphocytes, DCs, and Macrophages M1 cells), and had a strong association with high expression of immune factors (*IFN-γ*, *IL1*, *TNF*). In addition, it was found that *MAGEA4* had a high expression in the high-TR-DDR score group. The low-TR-DDR tumor was strongly correlated with a high level of M2-like macrophages and high expression of *TGF-β*. The results in the present study combing with the previous research further revealed that abnormality of DDR pathways might cause increased immune cell infiltration and cytotoxicity by promoting genomic instability (**Figure 6**). Secondly, it is widely accepted that DCs promote tumor immunity through antigen processing and presentation (101). The emergence of these “alien” peptides consequently brings higher immunogenicity to the tumor and better immunotherapy efficacy (73). In our results, high infiltration level of activated DCs was found in the high-TR-DDR group, linked with high immunogenicity of tumors. Therefore, the reason for the predictive ability of the TR-DDR score for chemotherapy or immunotherapy resistance may be explained by high immunogenicity caused by the effects of abnormal DDR pathways on the tumor immune microenvironment (**Figure 6**). Thirdly, we also noticed that mutations of certain DDR gene (*TP53*) correlate with increased *PDL1* expression in a cohort of lung adenocarcinoma patients (102). In our study, the significantly higher expression of immune checkpoint genes, including *PDL1*, *PD1*, and *CTLA4*, and elevation of the point mutation rate of *TP53* were found in high-TR-DDR score groups, which indicated that mutations of DDR genes in a tumor might influence treatment efficacy through altering expression levels of immune checkpoint molecules (**Figure 6**). Fourthly, previous studies revealed that both *COL2A1*, which encodes a component of type-II collagen and collages (103, 104), and *ZIC1* could suppress cancer metastasis by regulating the signaling pathway of the extracellular collagen-derived antiangiogenic factor (105, 106) and Wnt signaling pathway (107) respectively. While, FCRL4+ B cell is capable of aborting B cell receptor-mediating signaling and proliferation and producing pro-inflammatory cytokines *TNF-α (* 108, 109). In our study, the expressions of these four DEGs have strong associations with most of the core DDR genes. Therefore, *COL2A1*, *FCRL4*, *ZIC1*, and *MAGEA4* (discussed earlier) may play roles in the immune microenvironment between TR-DDR high- and low-score groups in our speculation (**Figure 6**). In conclusion, we systematically analyzed and speculated the possible regulatory mechanisms of DDR genes on the immune microenvironment. Nevertheless, these possible mechanisms still need further verification in the future.

Taken together, the combination of TR-DDR, TIL scores and G2M checkpoint score can optimize the accuracy of predicting chemotherapy or immunotherapy response in several cancer types. The possible regulatory mechanism of DDR genes on the immune microenvironment was speculated.

## Data availability statement

The original contributions presented in the study are included in the article/**Supplementary Material**. Further inquiries can be directed to the corresponding authors.

## Author contributions

All authors contributed significantly to the work and the preparation of the manuscript. HD, BX, and KD conceived the study. KD analyzed the data with assistance from YH, ZC, and HZ. KD wrote the manuscript. HD, JinW, SF, JiaW, DC, and YQ reviewed the manuscript. HD, JW, KL, XG, and LQ guided the proposed model. All authors contributed to the article and approved the submitted version.

## Funding

This work was supported by the National Key R&D Program of China (2018YFC0910201), the Key R&D Program of Guangdong Province (2019B020226001), the National Natural Science Foundation of China (11772133), China Postdoctoral Science Foundation (2021M701253) and funds from the Double First-Class University Plan (x2sw-k5183480).

## Conflict of interest

The authors declare that the research was conducted in the absence of any commercial or financial relationships that could be construed as a potential conflict of interest.

## Publisher’s note

All claims expressed in this article are solely those of the authors and do not necessarily represent those of their affiliated organizations, or those of the publisher, the editors and the reviewers. Any product that may be evaluated in this article, or claim that may be made by its manufacturer, is not guaranteed or endorsed by the publisher.

## Abbreviations

DDR, DNA damage repair; TR-DDR score, Tumor-related DNA damage repair score; BER, Base excision repair; NER, Nucleotide excision repair; MMR, Mismatch repair; FA, Fanconi anemia; HDR, Homology-dependent recombination; NHEJ, Non-homologous end-joining; DR, Direct repair; TLS, Translesion synthesis; DSB, The double-strand break; SSB, The single-strand break; TILs, Tumor-infiltrating lymphocytes; SNVs, Somatic single-nucleotide variants; CNAs, Copy number alterations; IC50, The half-maximal inhibitory concentration; TMB, Tumor mutation burden; DEGs, Differentially expressed genes; GSEA, Gene set enrichment analysis; GSVA, Gene set variation; ROC, The receiver operating characteristic; AUC, The area under the ROC curve; GO, Gene ontology; KEGG, Kyoto encyclopedia of genes and genomes; TCGA, The Cancer Genome Atlas; NCBI GEO, National Center for Biotechnology Information Gene Expression Omnibus; MSigDB, Molecular Signatures Database; CCLE, Cancer Cell Line Encyclopedia database; GDSC, Genomics of Drug Sensitivity in Cancer; BRCA, Breast invasive carcinoma; COAD, Colon adenocarcinoma; HNSC, Head and neck squamous cell carcinoma; KIRC, Kidney renal clear cell carcinoma; KIRP, Kidney renal papillary cell carcinoma; LIHC, Liver hepatocellular carcinoma; LUAD, Lung adenocarcinoma; LUSC, Lung squamous cell carcinoma; PRAD, Prostate adenocarcinoma; STAD, Stomach adenocarcinoma; THCA, Thyroid carcinoma
