# Supplementary material for: A score of DNA damage repair pathway with the predictive ability for chemotherapy and immunotherapy is strongly associated with immune signaling pathway in pan-cancer
Source: Front Immunol. 2022 Aug 23;13:943090. doi: 10.3389/fimmu.2022.943090 (PMC9445361; doi:10.3389/fimmu.2022.943090)
Supplement: Supplementary file 1 [file Image_1.pdf]

TCGA−BRCA

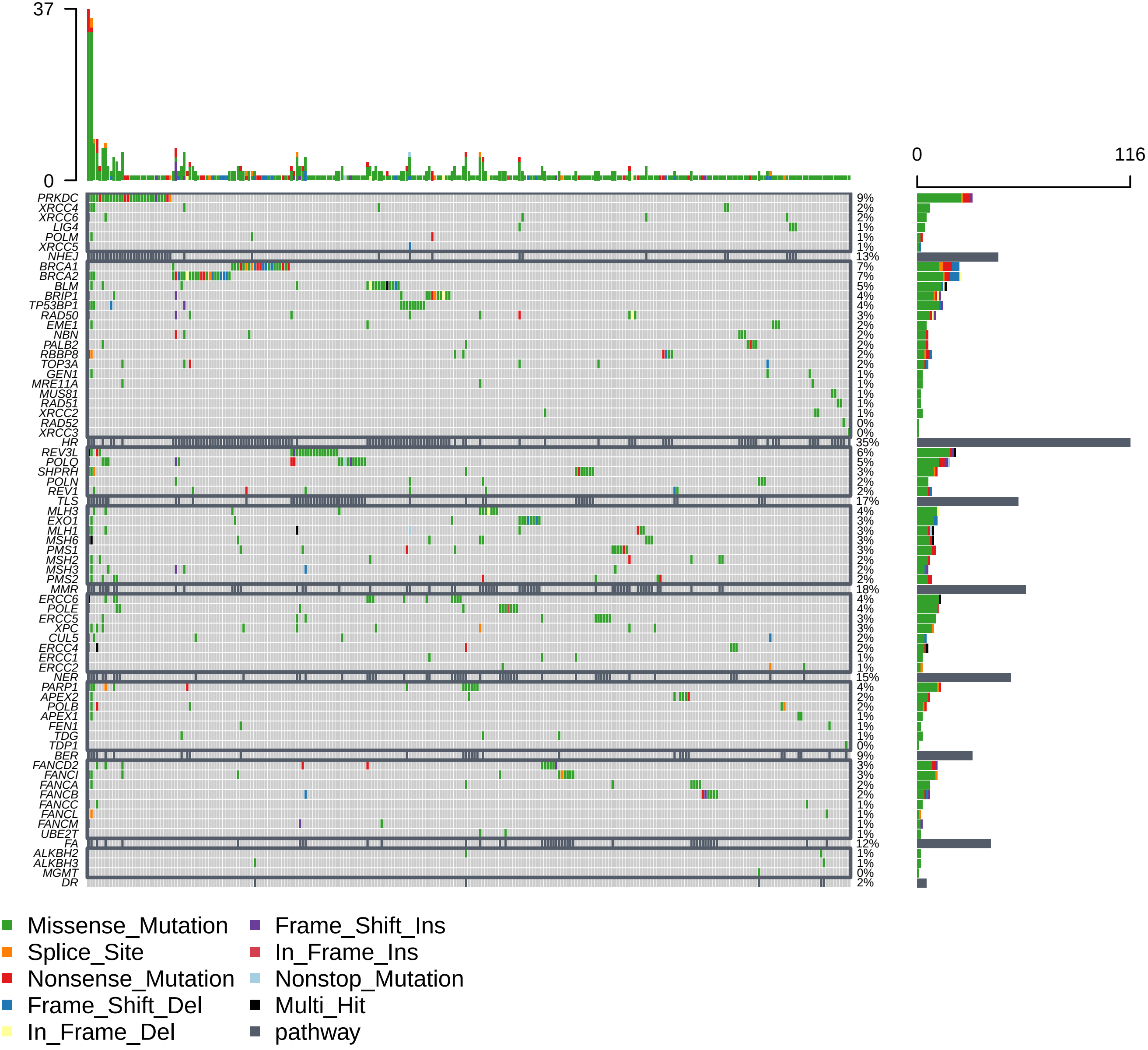

37
0
0
116
PRKDC 9%
XRCC4 2%
XRCC6 2%
LIG4 1%
POLM 1%
XRCC5 1%
NHEJ 13%
BRCA1 7%
BRCA2 7%
BLM 5%
BRIP1 4%
TP53BP1 4%
RAD50 3%
EME1 2%
NBN 2%
PALB2 2%
RBBP8 2%
TOP3A 2%
GEN1 1%
MRE11A 1%
MUS81 1%
RAD51 1%
XRCC2 1%
RAD52 0%
XRCC3 0%
HR 35%
REV3L 6%
POLQ 5%
SHPRH 3%
POLN 2%
REV1 2%
TLS 17%
MLH3 4%
EXO1 3%
MLH1 3%
MSH6 3%
PMS1 3%
MSH2 2%
MSH3 2%
PMS2 2%
MMR 18%
ERCC6 4%
POLE 4%
ERCC5 3%
XPC 3%
CUL5 2%
ERCC4 2%
ERCC1 1%
ERCC2 1%
NER 15%
PARP1 4%
APEX2 2%
POLB 2%
APEX1 1%
FEN1 1%
TDG 1%
TDP1 0%
BER 9%
FANCD2 3%
FANCI 3%
FANCA 2%
FANCB 2%
FANCC 1%
FANCL 1%
FANCM 1%
UBE2T 1%
FA 12%
ALKBH2 1%
ALKBH3 1%
MGMT 0%
DR 2%
Missense_Mutation
Splice_Site
Nonsense_Mutation
Frame_Shift_Del
In_Frame_Del
Frame_Shift_Ins
In_Frame_Ins
Nonstop_Mutation
Multi_Hit
pathway

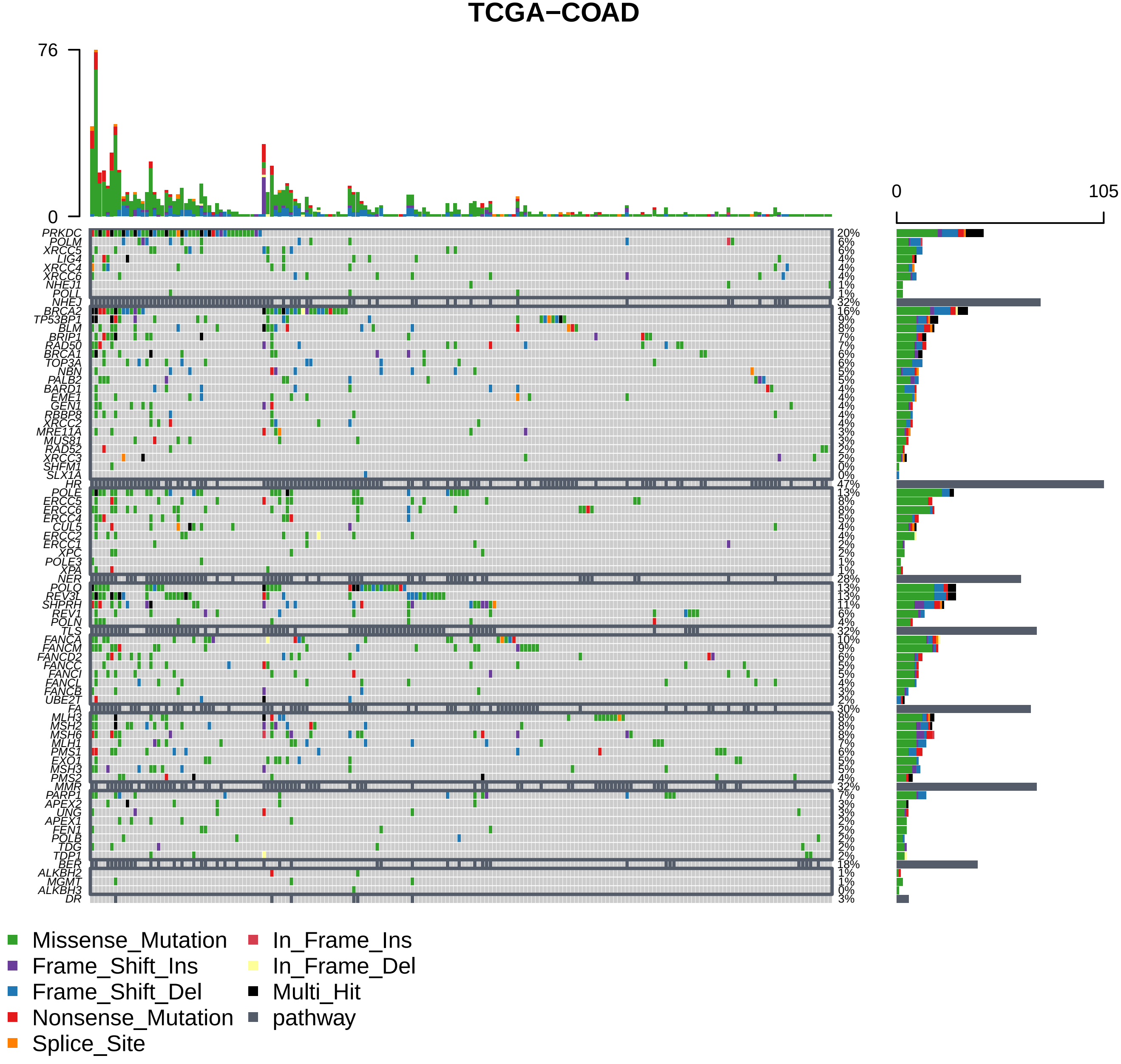
TCGA−COAD
76
0
0
105
PRKDC
POLM
XRCC5
LIG4
XRCC4
XRCC6
NHEJ1
POLL
NHEJ
BRCA2
TP53BP1
BLM
BRIP1
RAD50
BRCA1
TOP3A
NBN
PALB2
BARD1
EME1
GEN1
RBBP8
XRCC2
MRE11A
MUS81
RAD52
XRCC3
SHFM1
SLX1A
HR
POLE
ERCC5
ERCC6
ERCC4
CUL5
ERCC2
ERCC1
XPC
POLE3
XPA
NER
POLQ
REV3L
SHPRH
REV1
POLN
TLS
FANCA
FANCM
FANCD2
FANCC
FANCI
FANCL
FANCB
UBE2T
FA
MLH3
MSH2
MSH6
MLH1
PMS1
EXO1
MSH3
PMS2
MMR
PARP1
APEX2
UNG
APEX1
FEN1
POLB
TDG
TDP1
BER
ALKBH2
MGMT
ALKBH3
DR
20%
6%
6%
4%
4%
4%
1%
1%
32%
16%
9%
8%
7%
7%
6%
6%
5%
5%
4%
4%
4%
4%
4%
3%
3%
2%
2%
0%
0%
47%
13%
8%
8%
5%
4%
4%
2%
2%
1%
1%
28%
13%
13%
11%
6%
4%
32%
10%
9%
6%
5%
5%
4%
3%
2%
30%
8%
8%
8%
7%
6%
5%
5%
4%
32%
7%
3%
3%
2%
2%
2%
2%
2%
18%
1%
1%
0%
3%
Missense_Mutation
Frame_Shift_Ins
Frame_Shift_Del
Nonsense_Mutation
Splice_Site
In_Frame_Ins
In_Frame_Del
Multi_Hit
pathway

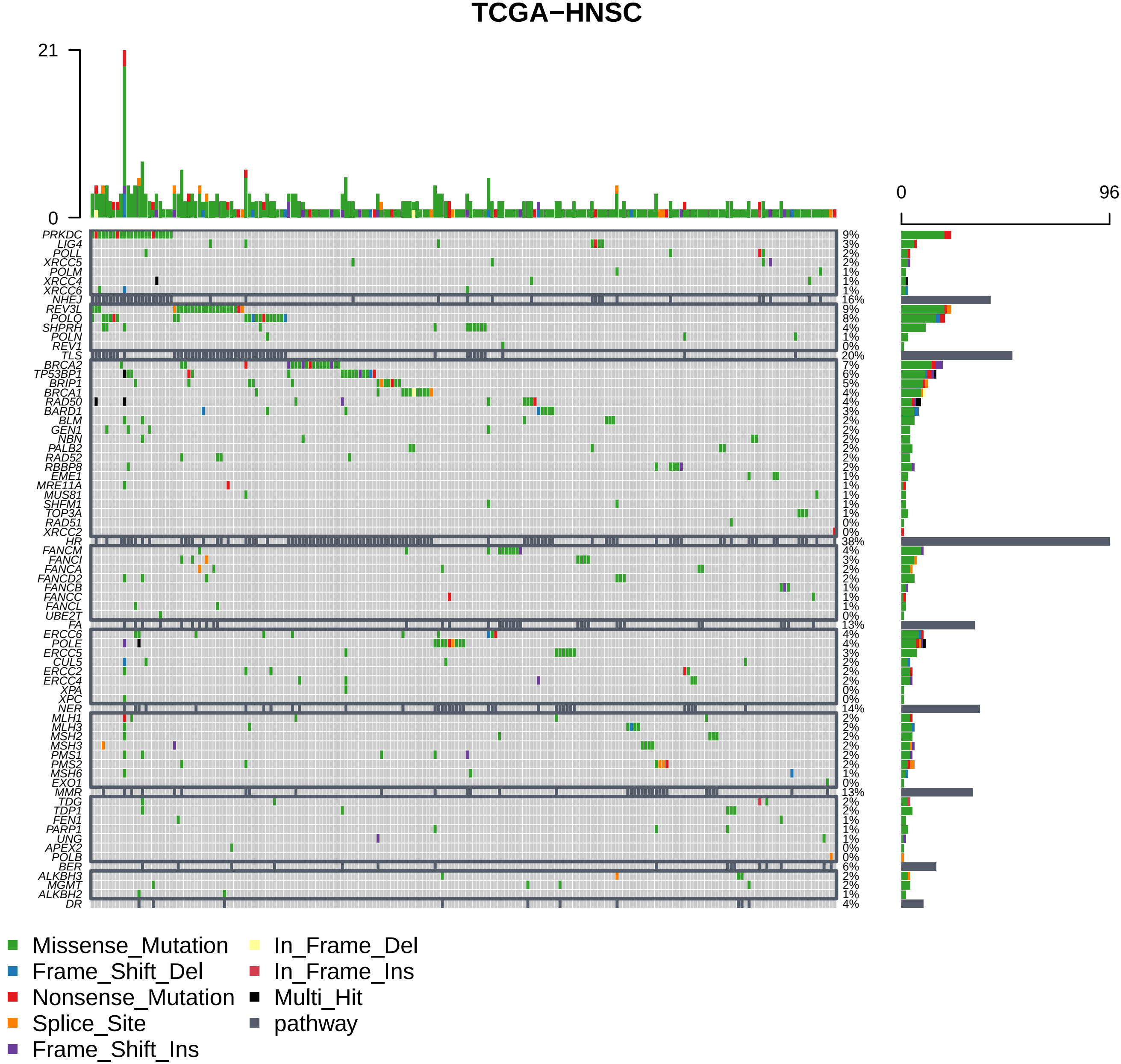
TCGA−HNSC
21
0
0
96
PRKDC 9%
LIG4 3%
POLL 2%
XRCC5 2%
POLM 1%
XRCC4 1%
XRCC6 1%
NHEJ 16%
REV3L 9%
POLQ 8%
SHPRH 4%
POLN 1%
REV1 0%
TLS 20%
BRCA2 7%
TP53BP1 6%
BRIP1 5%
BRCA1 4%
RAD50 4%
BARD1 3%
BLM 2%
GEN1 2%
NBN 2%
PALB2 2%
RAD52 2%
RBBP8 2%
EME1 1%
MRE11A 1%
MUS81 1%
SHFM1 1%
TOP3A 1%
RAD51 0%
XRCC2 0%
HR 38%
FANCM 4%
FANCI 3%
FANCA 2%
FANCD2 2%
FANCB 1%
FANCC 1%
FANCL 1%
UBE2T 0%
FA 13%
ERCC6 4%
POLE 4%
ERCC5 3%
CUL5 2%
ERCC2 2%
ERCC4 2%
XPA 0%
XPC 0%
NER 14%
MLH1 2%
MLH3 2%
MSH2 2%
MSH3 2%
PMS1 2%
PMS2 2%
MSH6 1%
EXO1 0%
MMR 13%
TDG 2%
TDP1 2%
FEN1 1%
PARP1 1%
UNG 1%
APEX2 0%
POLB 0%
BER 6%
ALKBH3 2%
MGMT 2%
ALKBH2 1%
DR 4%
Missense_Mutation
Frame_Shift_Del
Nonsense_Mutation
Splice_Site
Frame_Shift_Ins
In_Frame_Del
In_Frame_Ins
Multi_Hit
pathway

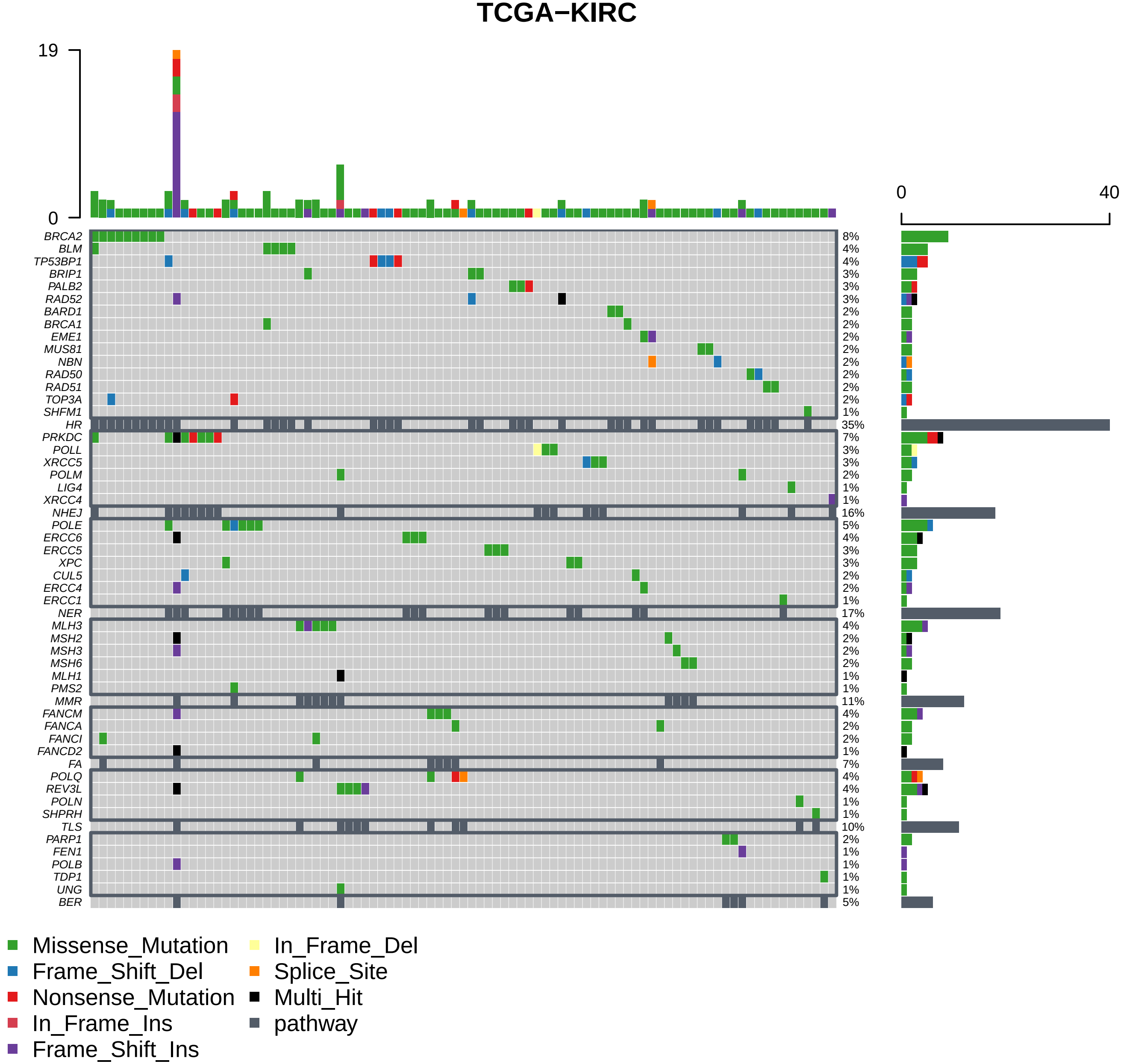

TCGA−KIRC
19
0
40
BRCA2 8%
BLM 4%
TP53BP1 4%
BRIP1 3%
PALB2 3%
RAD52 3%
BARD1 2%
BRCA1 2%
EME1 2%
MUS81 2%
NBN 2%
RAD50 2%
RAD51 2%
TOP3A 2%
SHFM1 1%
HR 35%
PRKDC 7%
POLL 3%
XRCC5 3%
POLM 2%
LIG4 1%
XRCC4 1%
NHEJ 16%
POLE 5%
ERCC6 4%
ERCC5 3%
XPC 3%
CUL5 2%
ERCC4 2%
ERCC1 1%
NER 17%
MLH3 4%
MSH2 2%
MSH3 2%
MSH6 2%
MLH1 1%
PMS2 1%
MMR 11%
FANCM 4%
FANCA 2%
FANCI 2%
FANCD2 1%
FA 7%
POLQ 4%
REV3L 4%
POLN 1%
SHPRH 1%
TLS 10%
PARP1 2%
FEN1 1%
POLB 1%
TDP1 1%
UNG 1%
BER 5%
Missense_Mutation
Frame_Shift_Del
Nonsense_Mutation
In_Frame_Ins
Frame_Shift_Ins
In_Frame_Del
Splice_Site
Multi_Hit
pathway

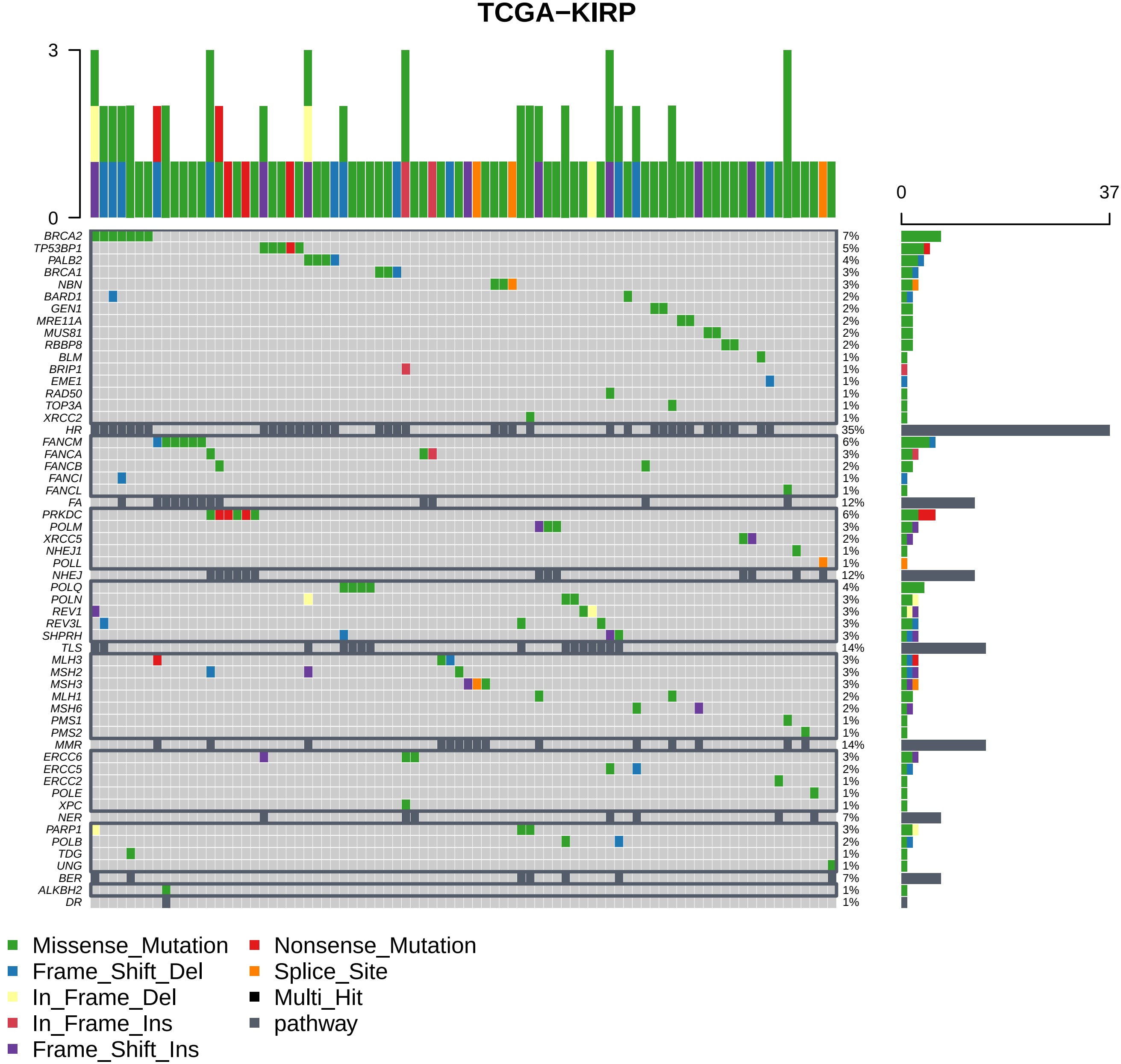
TCGA−KIRP
3
0
0
37
BRCA2 7%
TP53BP1 5%
PALB2 4%
BRCA1 3%
NBN 3%
BARD1 2%
GEN1 2%
MRE11A 2%
MUS81 2%
RBBP8 2%
BLM 1%
BRIP1 1%
EME1 1%
RAD50 1%
TOP3A 1%
XRCC2 1%
HR 35%
FANCM 6%
FANCA 3%
FANCB 2%
FANCI 1%
FANCL 1%
FA 12%
PRKDC 6%
POLM 3%
XRCC5 2%
NHEJ1 1%
POLL 1%
NHEJ 12%
POLQ 4%
POLN 3%
REV1 3%
REV3L 3%
SHPRH 3%
TLS 14%
MLH3 3%
MSH2 3%
MSH3 3%
MLH1 2%
MSH6 2%
PMS1 1%
PMS2 1%
MMR 14%
ERCC6 3%
ERCC5 2%
ERCC2 1%
POLE 1%
XPC 1%
NER 7%
PARP1 3%
POLB 2%
TDG 1%
UNG 1%
BER 7%
ALKBH2 1%
DR 1%
Missense_Mutation
Nonsense_Mutation
Frame_Shift_Del
Splice_Site
In_Frame_Del
Multi_Hit
In_Frame_Ins
pathway
Frame_Shift_Ins

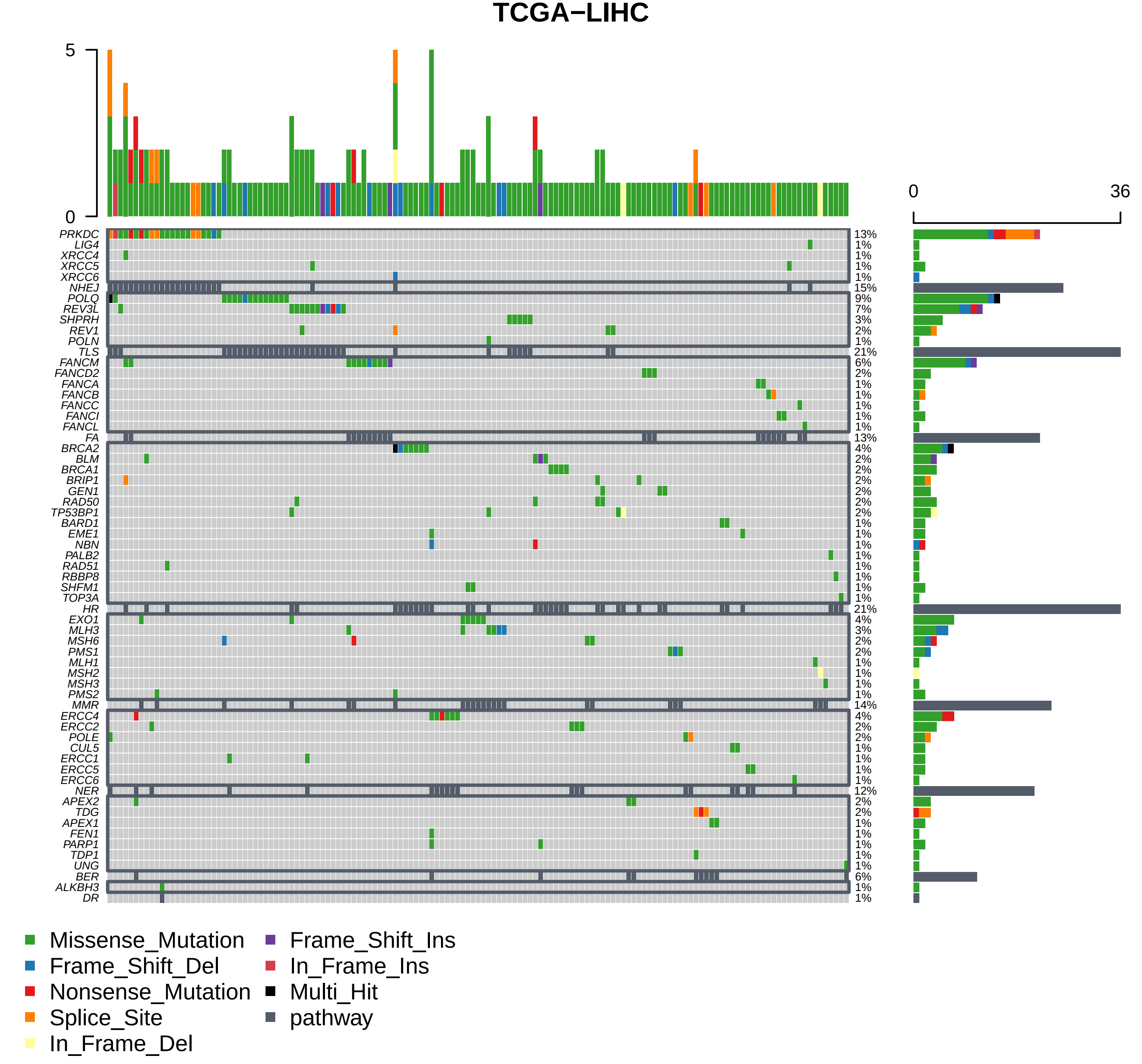

TCGA−LIHC
5
0
0
36
PRKDC 13%
LIG4 1%
XRCC4 1%
XRCC5 1%
XRCC6 1%
NHEJ 15%
POLQ 9%
REV3L 7%
SHPRH 3%
REV1 2%
POLN 1%
TLS 21%
FANCM 6%
FANCD2 2%
FANCA 1%
FANCB 1%
FANCC 1%
FANCI 1%
FANCL 1%
FA 13%
BRCA2 4%
BLM 2%
BRCA1 2%
BRIP1 2%
GEN1 2%
RAD50 2%
TP53BP1 2%
BARD1 1%
EME1 1%
NBN 1%
PALB2 1%
RAD51 1%
RBBP8 1%
SHFM1 1%
TOP3A 1%
HR 21%
EXO1 4%
MLH3 3%
MSH6 2%
PMS1 2%
MLH1 1%
MSH2 1%
MSH3 1%
PMS2 1%
MMR 14%
ERCC4 4%
ERCC2 2%
POLE 2%
CUL5 1%
ERCC1 1%
ERCC5 1%
ERCC6 1%
NER 12%
APEX2 2%
TDG 2%
APEX1 1%
FEN1 1%
PARP1 1%
TDP1 1%
UNG 1%
BER 6%
ALKBH3 1%
DR 1%
Missense_Mutation
Frame_Shift_Del
Nonsense_Mutation
Splice_Site
In_Frame_Del
Frame_Shift_Ins
In_Frame_Ins
Multi_Hit
pathway

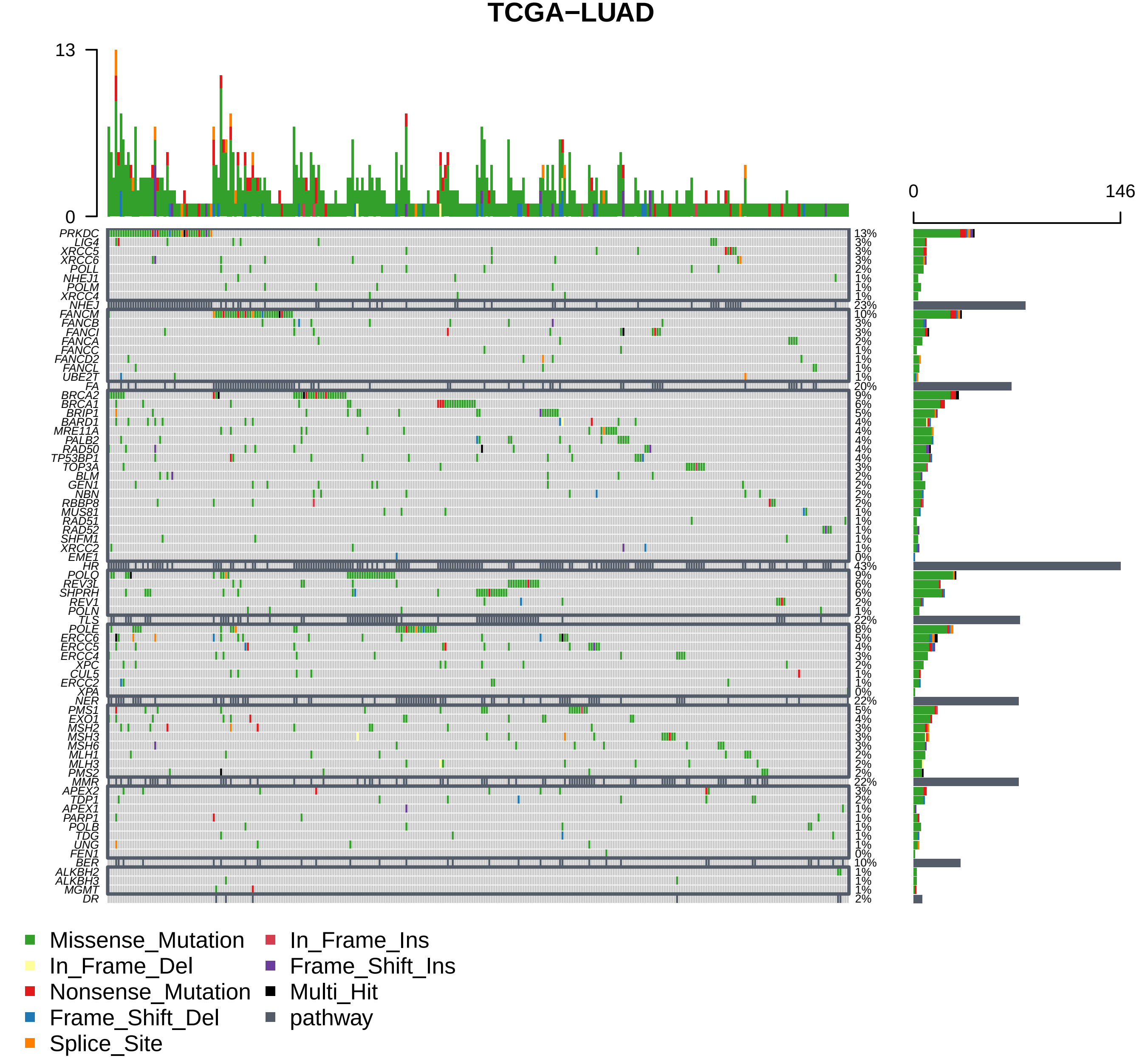
TCGA−LUAD
13
0
0
146
PRKDC 13%
LIG4 3%
XRCC5 3%
XRCC6 3%
POLL 2%
NHEJ1 1%
POLM 1%
XRCC4 1%
NHEJ 23%
FANCM 10%
FANCB 3%
FANCI 3%
FANCA 2%
FANCC 1%
FANCD2 1%
FANCL 1%
UBE2T 1%
FA 20%
BRCA2 9%
BRCA1 6%
BRIP1 5%
BARD1 4%
MRE11A 4%
PALB2 4%
RAD50 4%
TP53BP1 4%
TOP3A 3%
BLM 2%
GEN1 2%
NBN 2%
RBBP8 2%
MUS81 1%
RAD51 1%
RAD52 1%
SHFM1 1%
XRCC2 1%
EME1 0%
HR 43%
POLQ 9%
REV3L 6%
SHPRH 6%
REV1 2%
POLN 1%
TLS 22%
POLE 8%
ERCC6 5%
ERCC5 4%
ERCC4 3%
XPC 2%
CUL5 1%
ERCC2 1%
XPA 0%
NER 22%
PMS1 5%
EXO1 4%
MSH2 3%
MSH3 3%
MSH6 3%
MLH1 2%
MLH3 2%
PMS2 2%
MMR 22%
APEX2 3%
TDP1 2%
APEX1 1%
PARP1 1%
POLB 1%
TDG 1%
UNG 1%
FEN1 0%
BER 10%
ALKBH2 1%
ALKBH3 1%
MGMT 1%
DR 2%
Missense_Mutation
In_Frame_Del
Nonsense_Mutation
Frame_Shift_Del
Splice_Site
In_Frame_Ins
Frame_Shift_Ins
Multi_Hit
pathway

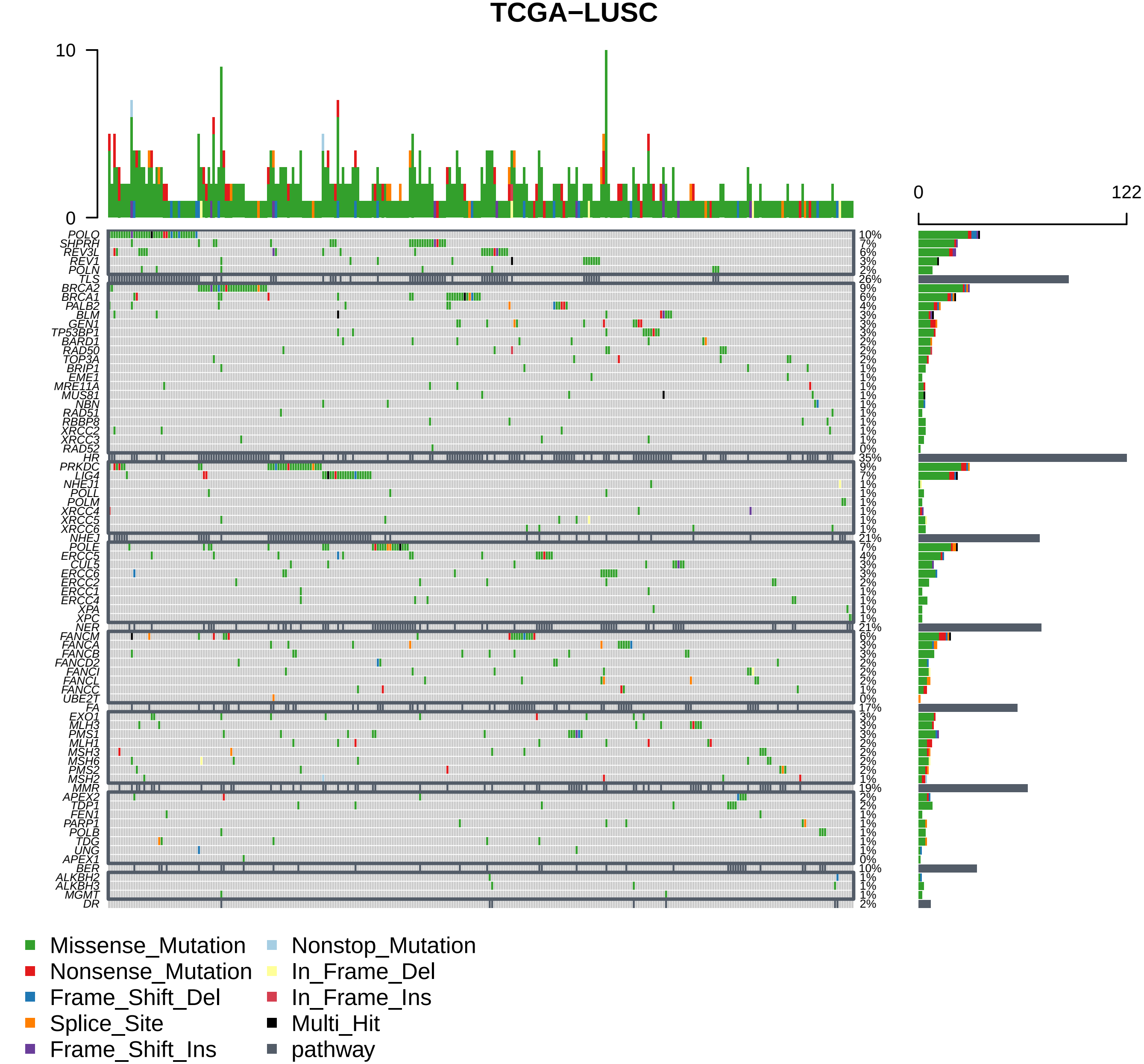
TCGA−LUSC
10
0
0
122
POLQ 10%
SHPRH 7%
REV3L 6%
REV1 3%
POLN 2%
TLS 26%
BRCA2 9%
BRCA1 6%
PALB2 4%
BLM 3%
GEN1 3%
TP53BP1 3%
BARD1 2%
RAD50 2%
TOP3A 2%
BRIP1 1%
EME1 1%
MRE11A 1%
MUS81 1%
NBN 1%
RAD51 1%
RBBP8 1%
XRCC2 1%
XRCC3 1%
RAD52 0%
HR 35%
PRKDC 9%
LIG4 7%
NHEJ1 1%
POLL 1%
POLM 1%
XRCC4 1%
XRCC5 1%
XRCC6 1%
NHEJ 21%
POLE 7%
ERCC5 4%
CUL5 3%
ERCC6 3%
ERCC2 2%
ERCC1 1%
ERCC4 1%
XPA 1%
XPC 1%
NER 21%
FANCM 6%
FANCA 3%
FANCB 3%
FANCD2 2%
FANCI 2%
FANCL 2%
FANCC 1%
UBE2T 0%
FA 17%
EXO1 3%
MLH3 3%
PMS1 3%
MLH1 2%
MSH3 2%
MSH6 2%
PMS2 2%
MSH2 1%
MMR 19%
APEX2 2%
TDP1 2%
FEN1 1%
PARP1 1%
POLB 1%
TDG 1%
UNG 1%
APEX1 0%
BER 10%
ALKBH2 1%
ALKBH3 1%
MGMT 1%
DR 2%
Missense_Mutation
Nonsense_Mutation
Frame_Shift_Del
Splice_Site
Frame_Shift_Ins
Nonstop_Mutation
In_Frame_Del
In_Frame_Ins
Multi_Hit
pathway

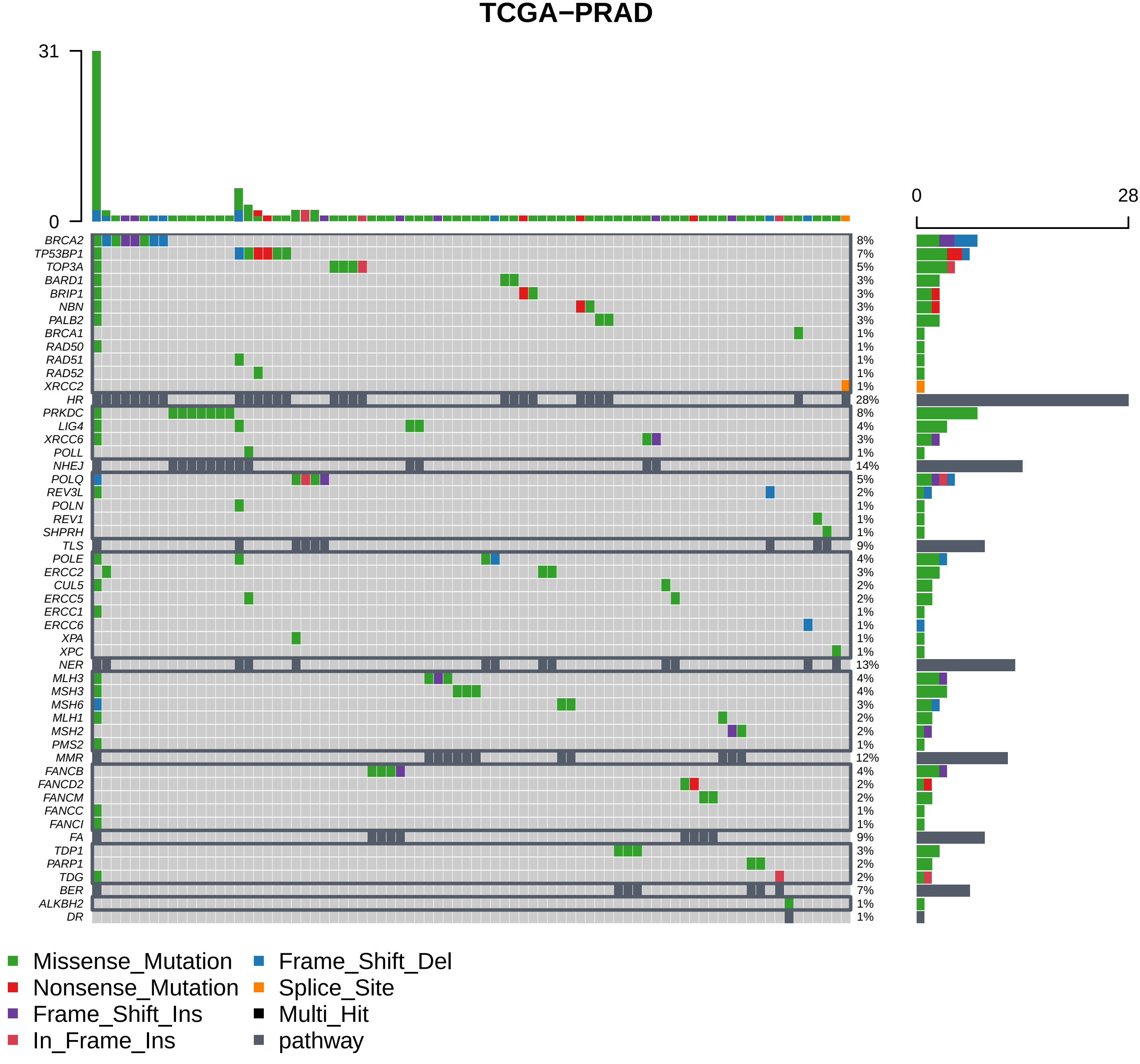
TCGA−PRAD
31
0
28
BRCA2 8%
TP53BP1 7%
TOP3A 5%
BARD1 3%
BRIP1 3%
NBN 3%
PALB2 3%
BRCA1 1%
RAD50 1%
RAD51 1%
RAD52 1%
XRCC2 1%
HR 28%
PRKDC 8%
LIG4 4%
XRCC6 3%
POLL 1%
NHEJ 14%
POLQ 5%
REV3L 2%
POLN 1%
REV1 1%
SHPRH 1%
TLS 9%
POLE 4%
ERCC2 3%
CUL5 2%
ERCC5 2%
ERCC1 1%
ERCC6 1%
XPA 1%
XPC 1%
NER 13%
MLH3 4%
MSH3 4%
MSH6 3%
MLH1 2%
MSH2 2%
PMS2 1%
MMR 12%
FANCB 4%
FANCD2 2%
FANCM 2%
FANCC 1%
FANCI 1%
FA 9%
TDP1 3%
PARP1 2%
TDG 2%
BER 7%
ALKBH2 1%
DR 1%
Missense_Mutation
Frame_Shift_Del
Nonsense_Mutation
Splice_Site
Frame_Shift_Ins
Multi_Hit
In_Frame_Ins
pathway

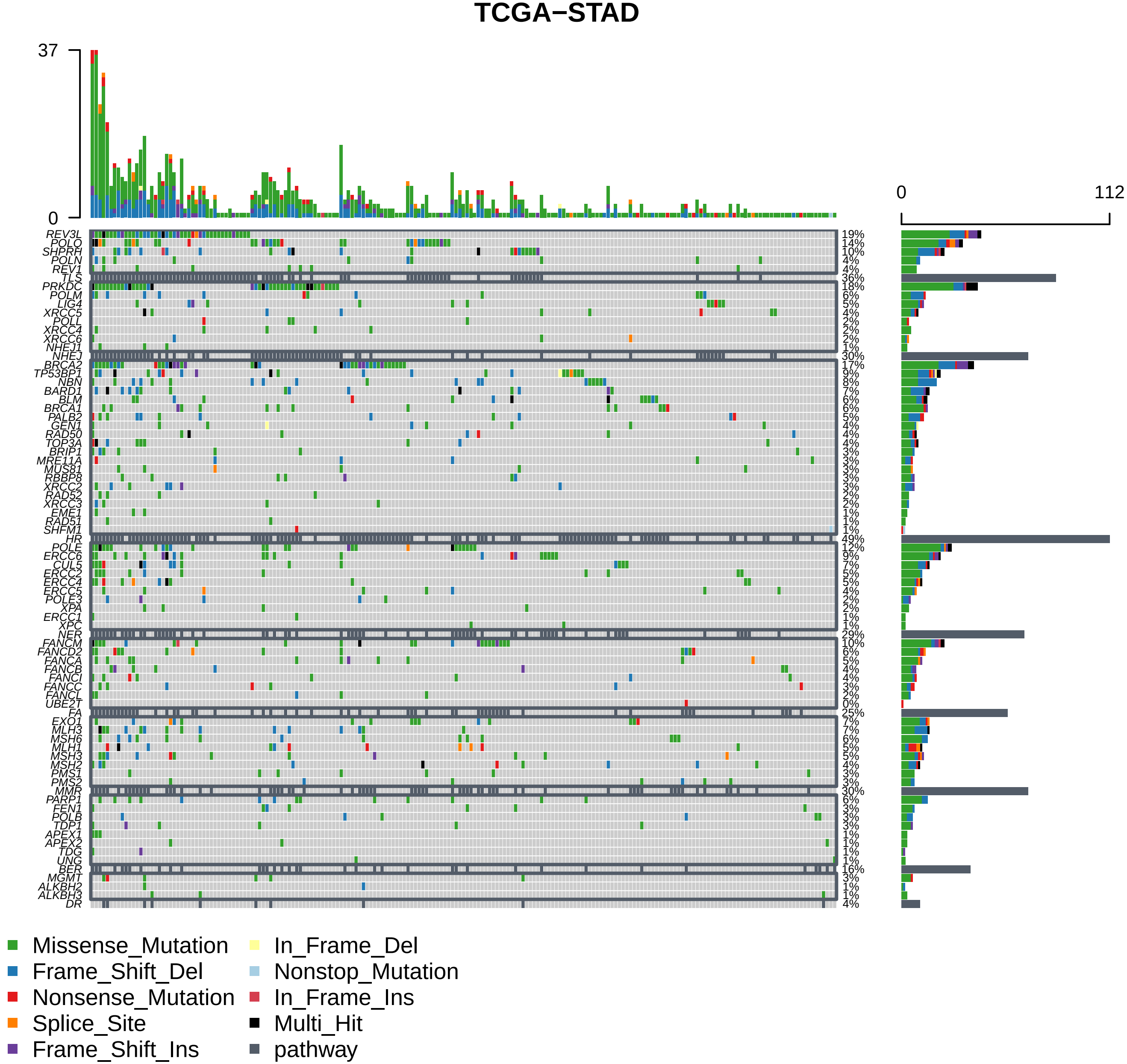

TCGA−STAD
37
0
0
112
REV3L 19%
POLQ 14%
SHPRH 10%
POLN 4%
REV1 4%
TLS 36%
PRKDC 18%
POLM 6%
LIG4 5%
XRCC5 4%
POLL 2%
XRCC4 2%
XRCC6 2%
NHEJ1 1%
NHEJ 30%
BRCA2 17%
TP53BP1 9%
NBN 8%
BARD1 7%
BLM 6%
BRCA1 6%
PALB2 5%
GEN1 4%
RAD50 4%
TOP3A 4%
BRIP1 3%
MRE11A 3%
MUS81 3%
RBBP8 3%
XRCC2 3%
RAD52 2%
XRCC3 2%
EME1 1%
RAD51 1%
SHFM1 1%
HR 49%
POLE 12%
ERCC6 9%
CUL5 7%
ERCC2 5%
ERCC4 5%
ERCC5 4%
POLE3 2%
XPA 2%
ERCC1 1%
XPC 1%
NER 29%
FANCM 10%
FANCD2 6%
FANCA 5%
FANCB 4%
FANCI 4%
FANCC 3%
FANCL 2%
UBE2T 0%
FA 25%
EXO1 7%
MLH3 7%
MSH6 6%
MLH1 5%
MSH3 5%
MSH2 4%
PMS1 3%
PMS2 3%
MMR 30%
PARP1 6%
FEN1 3%
POLB 3%
TDP1 3%
APEX1 1%
APEX2 1%
TDG 1%
UNG 1%
BER 16%
MGMT 3%
ALKBH2 1%
ALKBH3 1%
DR 4%
Missense_Mutation
Frame_Shift_Del
Nonsense_Mutation
Splice_Site
Frame_Shift_Ins
In_Frame_Del
Nonstop_Mutation
In_Frame_Ins
Multi_Hit
pathway

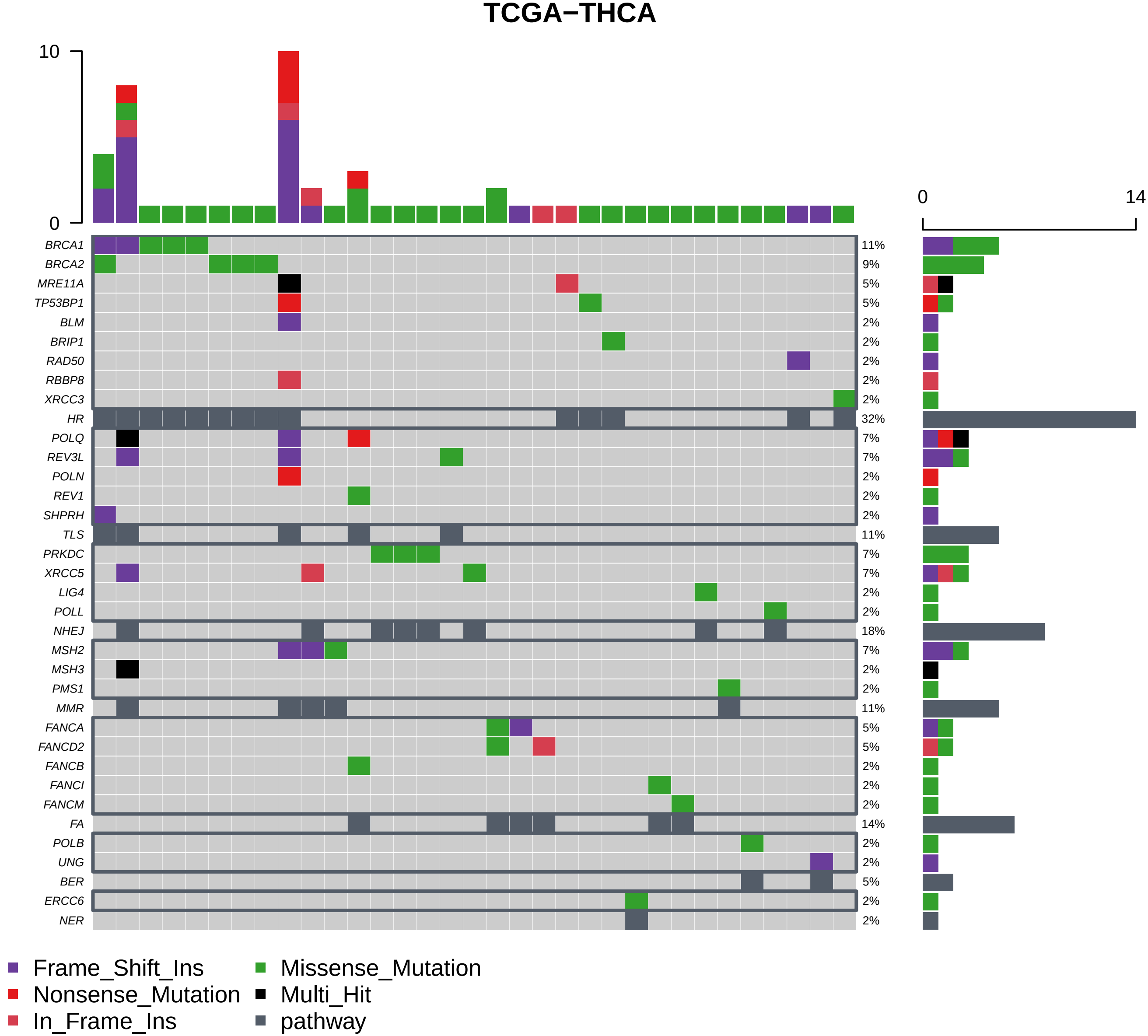
TCGA−THCA
10
0
0
14
BRCA1
BRCA2
MRE11A
TP53BP1
BLM
BRIP1
RAD50
RBBP8
XRCC3
HR
POLQ
REV3L
POLN
REV1
SHPRH
TLS
PRKDC
XRCC5
LIG4
POLL
NHEJ
MSH2
MSH3
PMS1
MMR
FANCA
FANCD2
FANCB
FANCI
FANCM
FA
POLB
UNG
BER
ERCC6
NER
11%
9%
5%
5%
2%
2%
2%
2%
2%
32%
7%
7%
2%
2%
2%
11%
7%
7%
2%
2%
18%
7%
2%
2%
11%
5%
5%
2%
2%
2%
14%
2%
2%
5%
2%
2%
Frame_Shift_Ins
Missense_Mutation
Nonsense_Mutation
Multi_Hit
In_Frame_Ins
pathway
