# Supplementary figures and images for: A score of DNA damage repair pathway with the predictive ability for chemotherapy and immunotherapy is strongly associated with immune signaling pathway in pan-cancer
Source: Front Immunol. 2022 Aug 23;13:943090. doi: 10.3389/fimmu.2022.943090 (PMC9445361; doi:10.3389/fimmu.2022.943090)

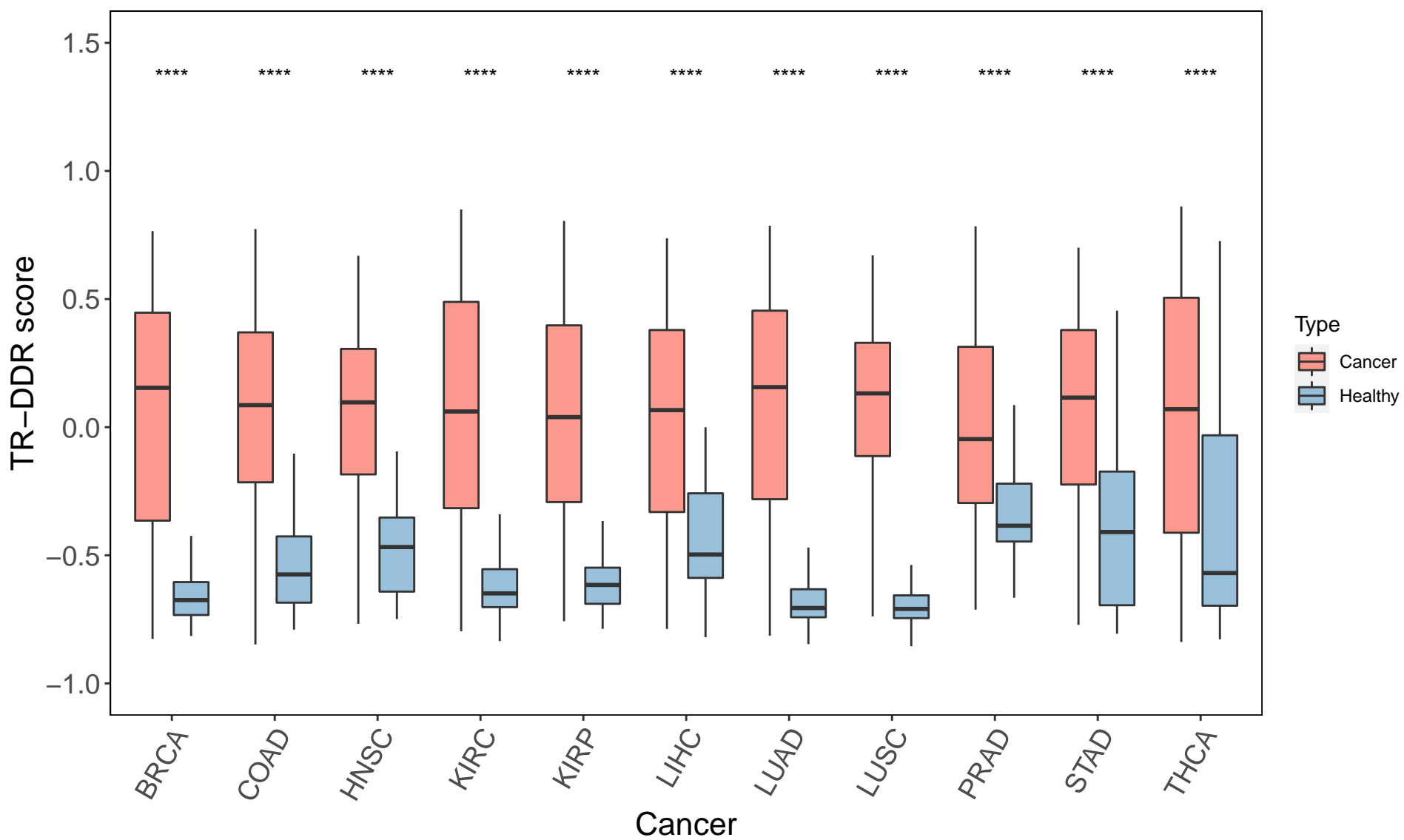

Supplement: Supplementary file 2 [file Image_2.pdf]

BRCA

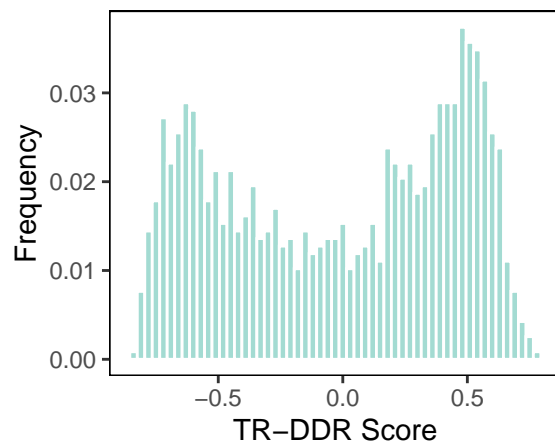

COAD

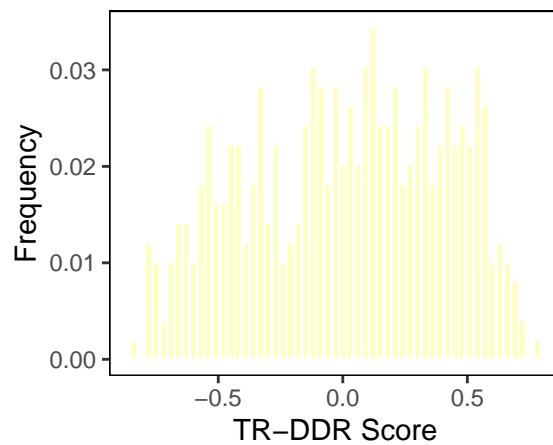

HNSC

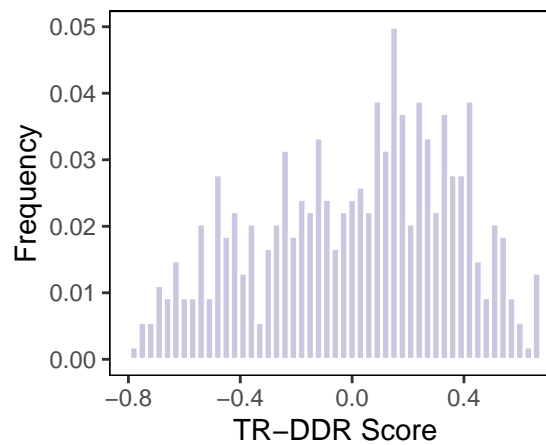

KIRC

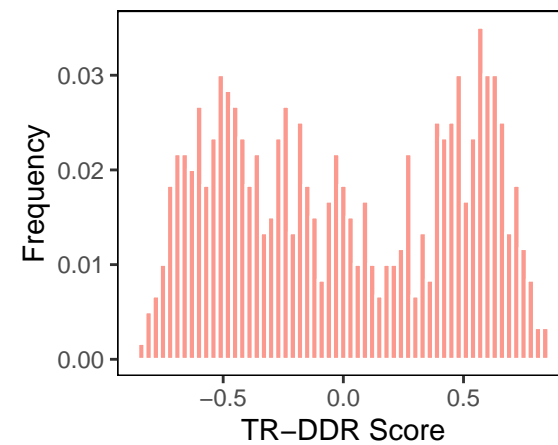

KIRP

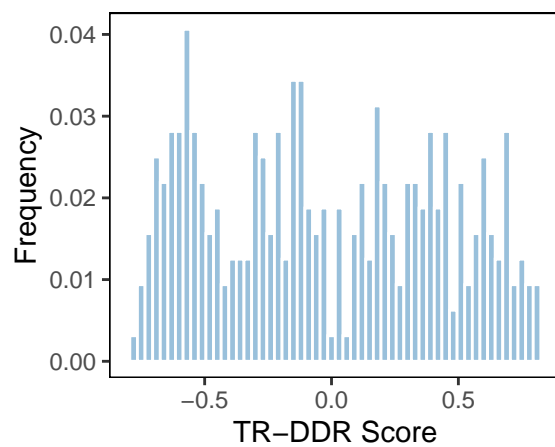

LIHC

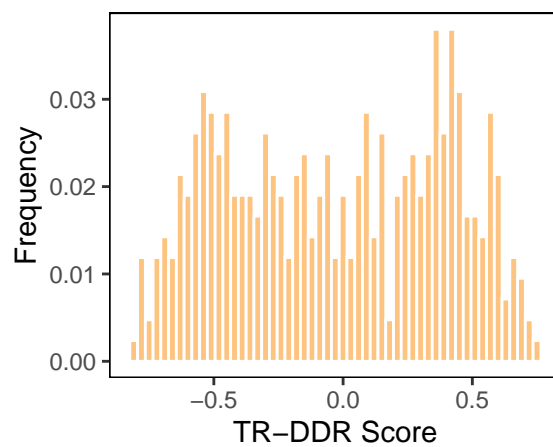

LUAD

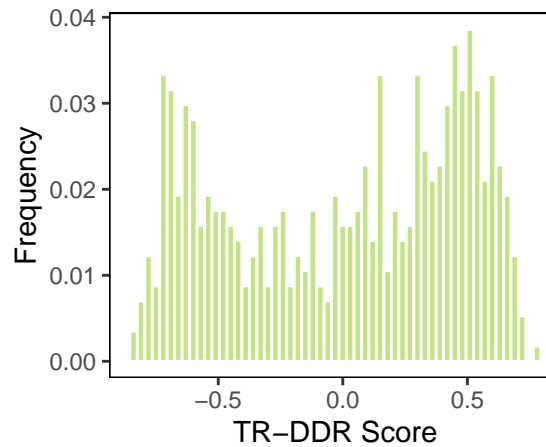

LUSC

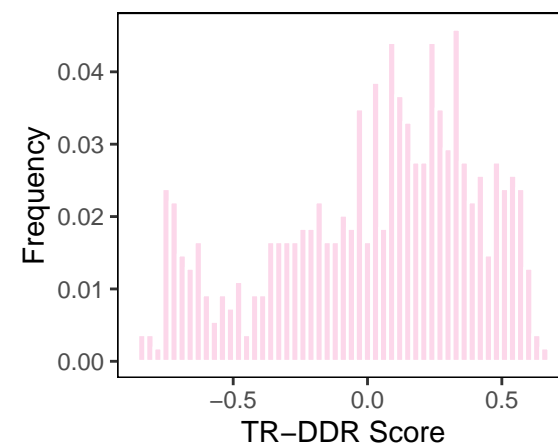

PRAD

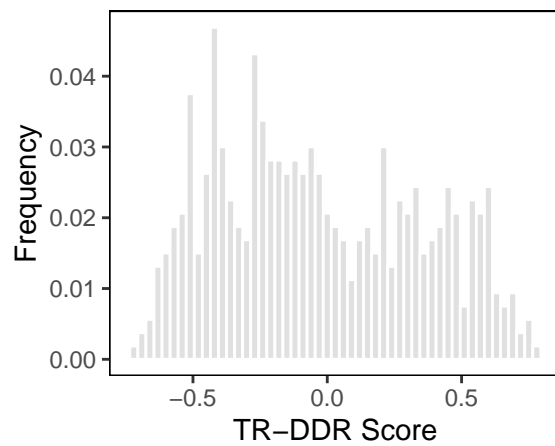

STAD

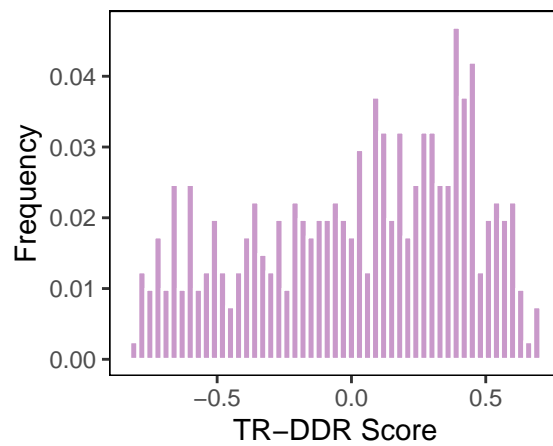

THCA

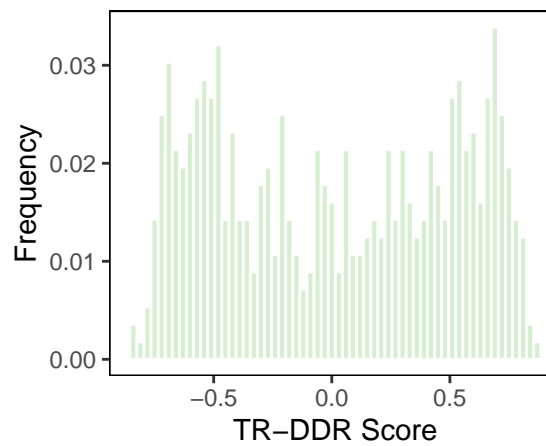

Supplement: Supplementary file 4 [file Image_4.pdf]

Group High Low

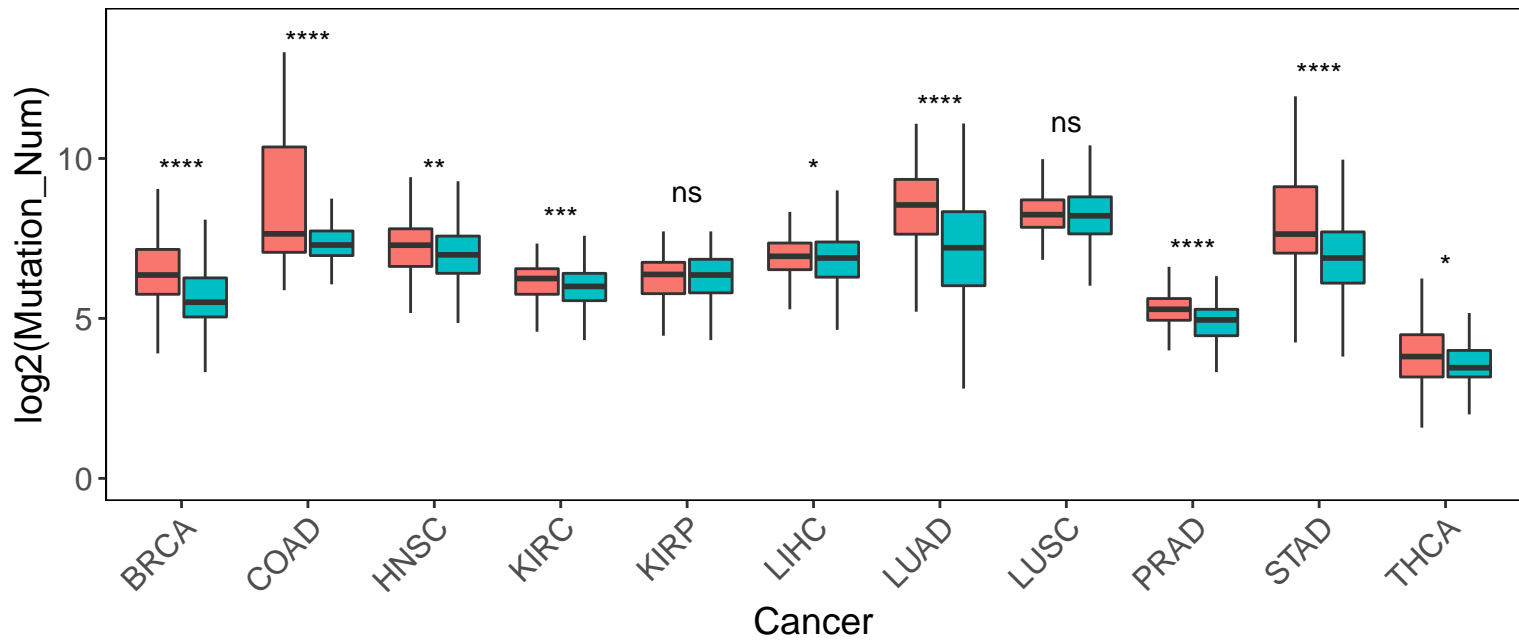

Supplement: Supplementary file 6 [file Image_6.pdf]

COL2A1.txt

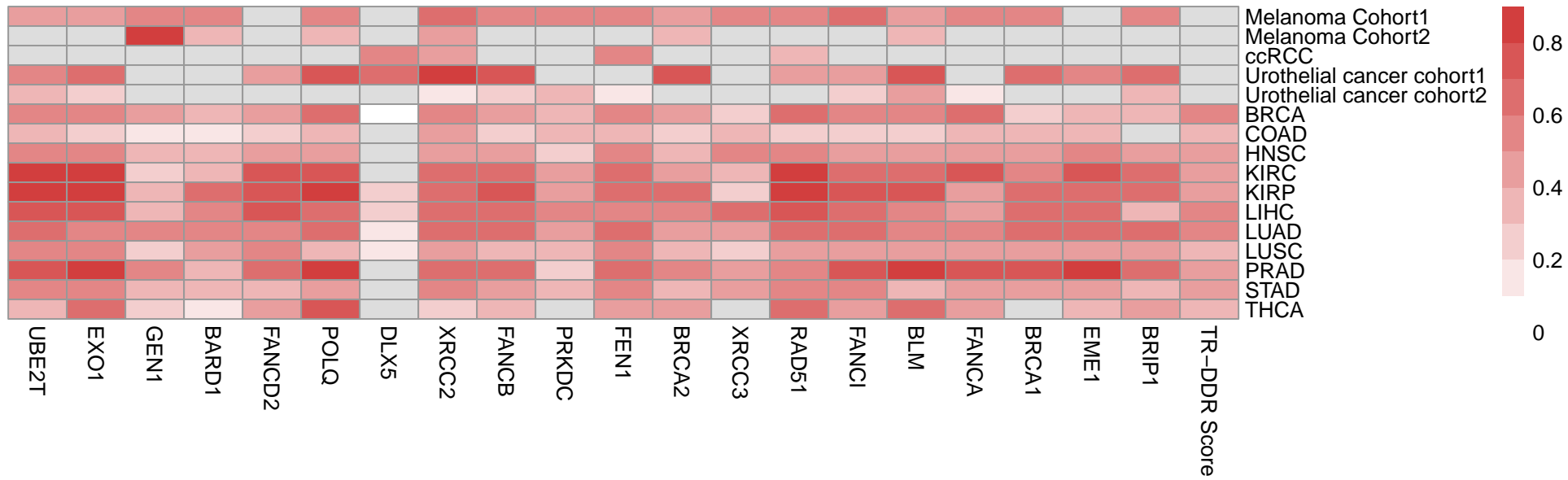

FCRL4.txt

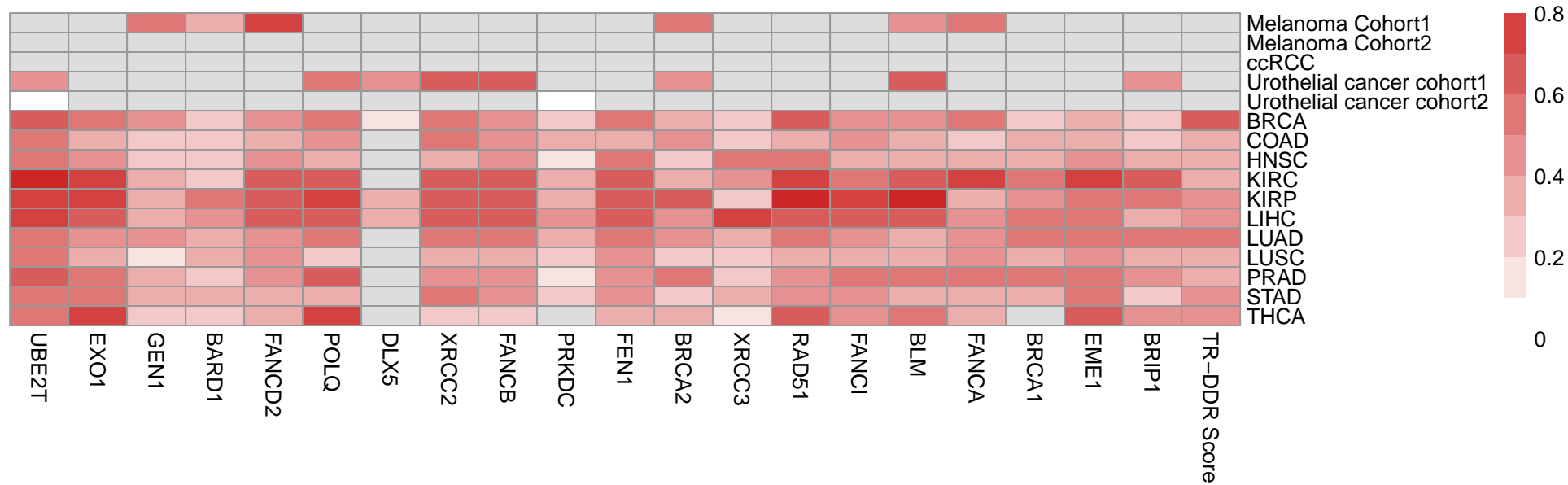

MAGEA4.txt

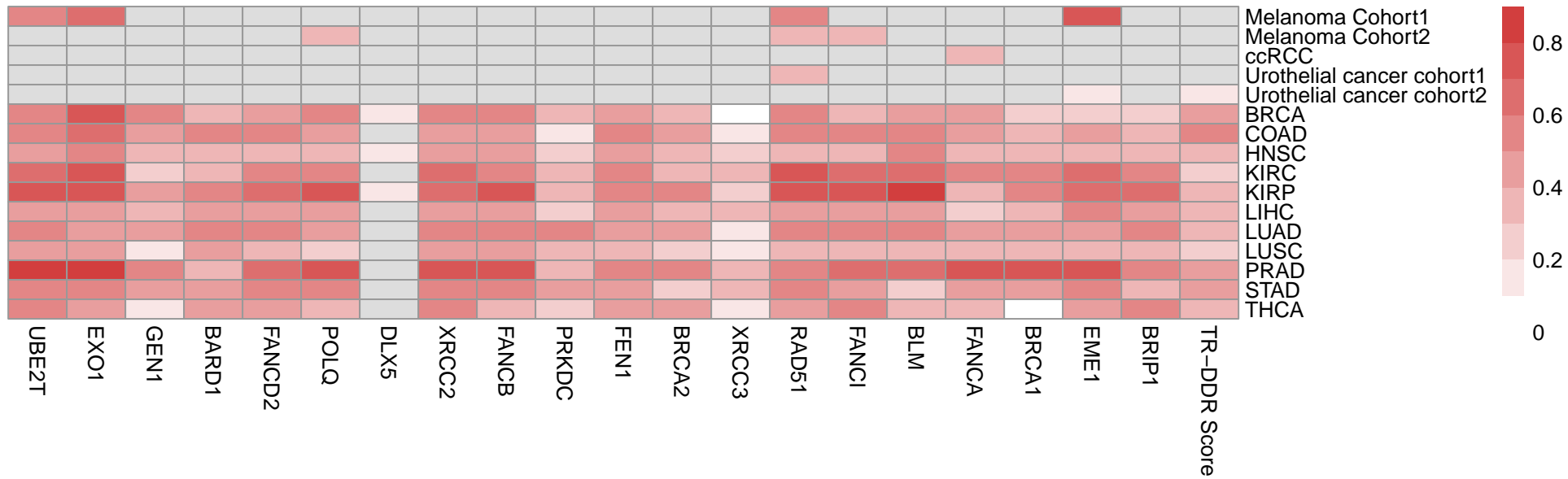

ZIC1.txt

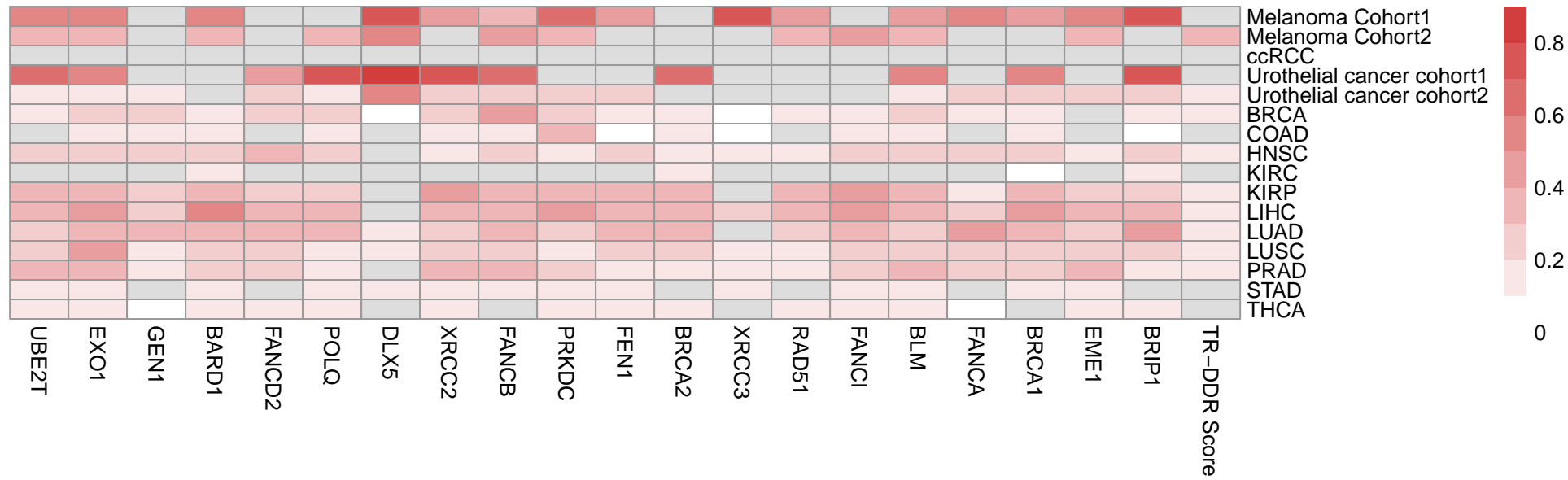

Supplement: Supplementary file 8 [file Image_8.pdf]
