# Supplementary material for: A score of DNA damage repair pathway with the predictive ability for chemotherapy and immunotherapy is strongly associated with immune signaling pathway in pan-cancer
Source: Front Immunol. 2022 Aug 23;13:943090. doi: 10.3389/fimmu.2022.943090 (PMC9445361; doi:10.3389/fimmu.2022.943090)
Supplement: Supplementary file 3 [file Image_3.pdf]

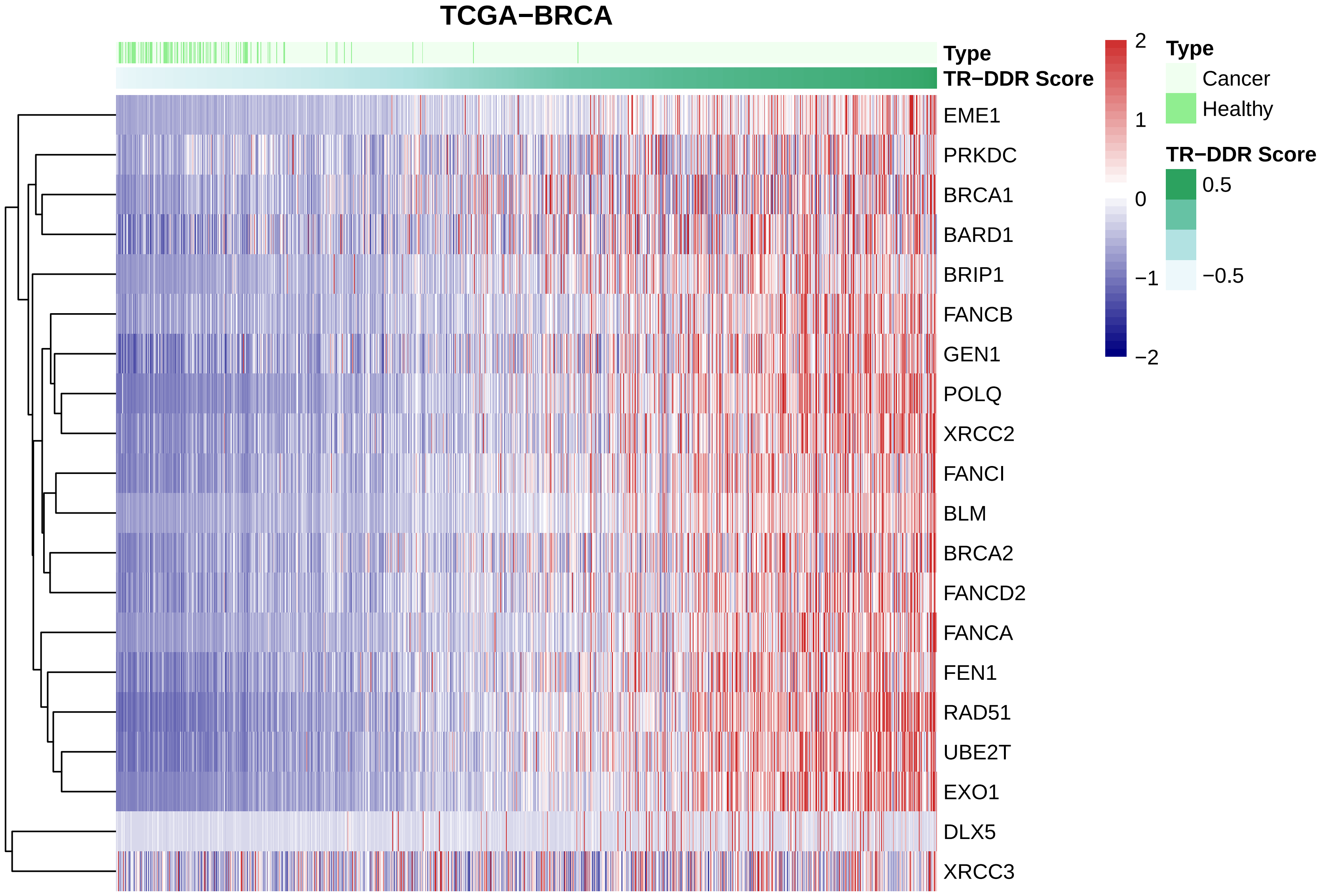

TCGA−BRCA
Type
TR−DDR Score
EME1
PRKDC
BRCA1
BARD1
BRIP1
FANCB
GEN1
POLQ
XRCC2
FANCI
BLM
BRCA2
FANCD2
FANCA
FEN1
RAD51
UBE2T
EXO1
DLX5
XRCC3
2
1
0
−1
−2
Type
Cancer
Healthy
TR−DDR Score
0.5
−0.5

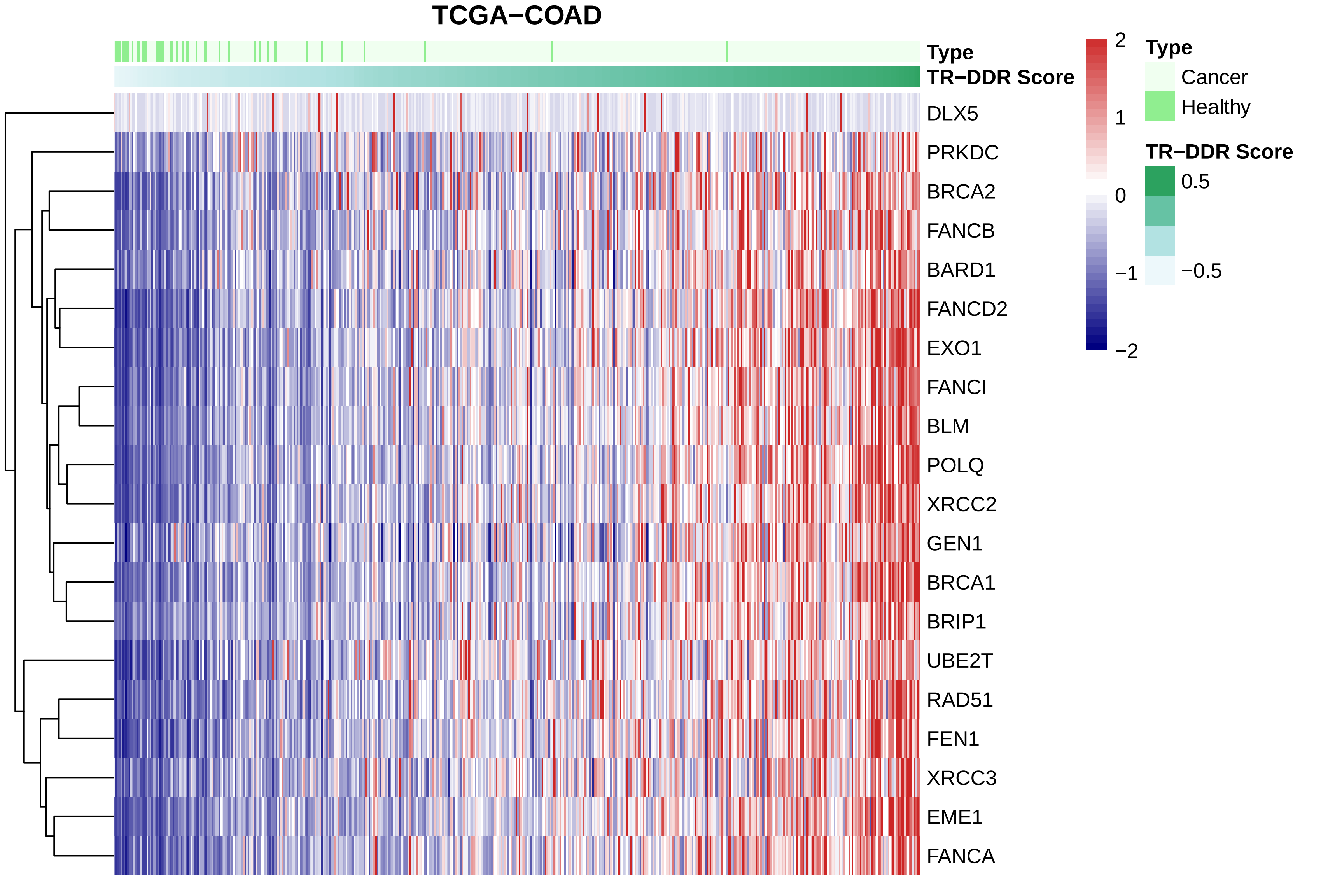

TCGA−COAD
Type
TR−DDR Score
DLX5
PRKDC
BRCA2
FANCB
BARD1
FANCD2
EXO1
FANCI
BLM
POLQ
XRCC2
GEN1
BRCA1
BRIP1
UBE2T
RAD51
FEN1
XRCC3
EME1
FANCA
2
1
0
−1
−2
Type
Cancer
Healthy
TR−DDR Score
0.5
−0.5

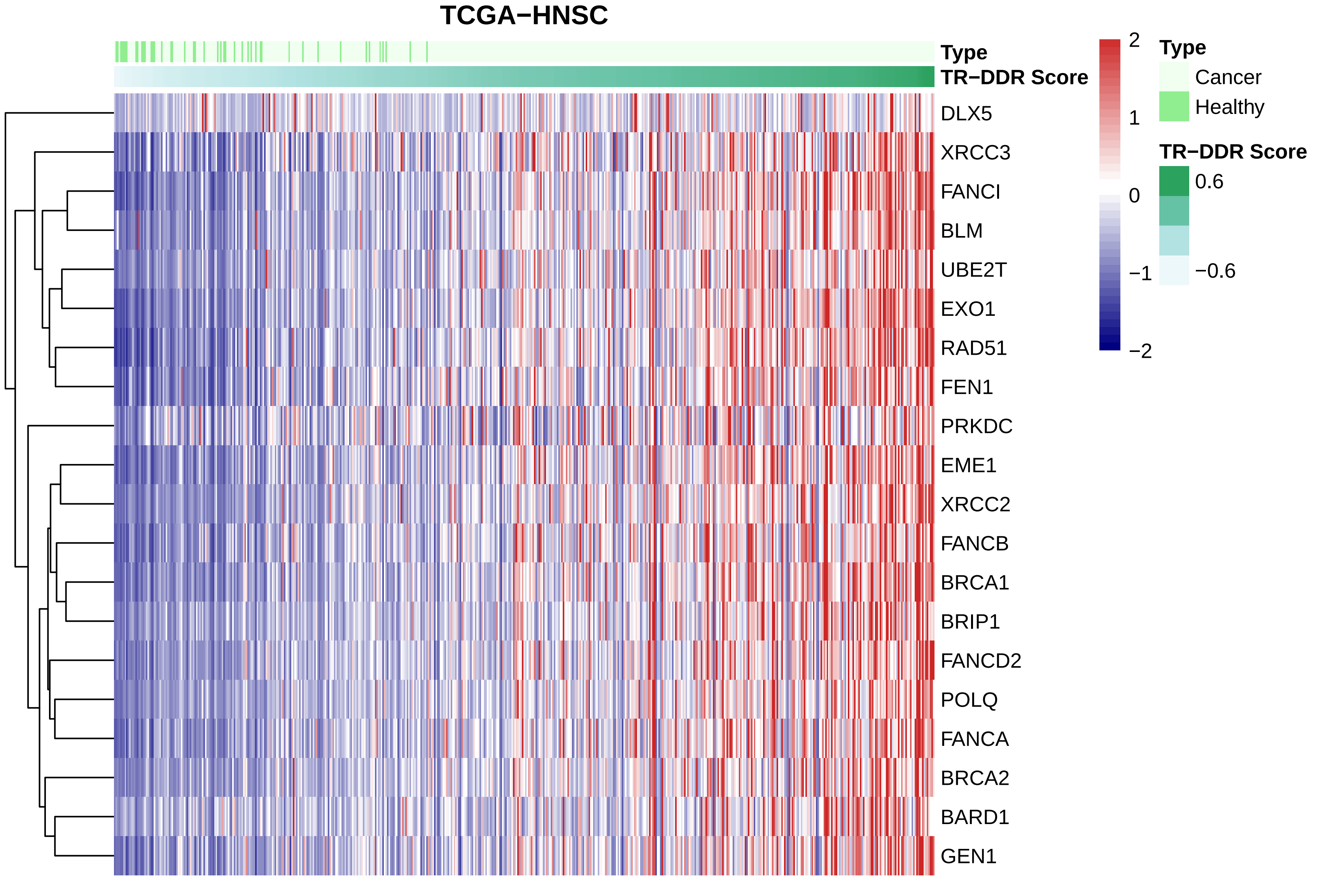

TCGA−HNSC
Type
TR−DDR Score
DLX5
XRCC3
FANCI
BLM
UBE2T
EXO1
RAD51
FEN1
PRKDC
EME1
XRCC2
FANCB
BRCA1
BRIP1
FANCD2
POLQ
FANCA
BRCA2
BARD1
GEN1
2
1
0
−1
−2
Type
Cancer
Healthy
TR−DDR Score
0.6
−0.6

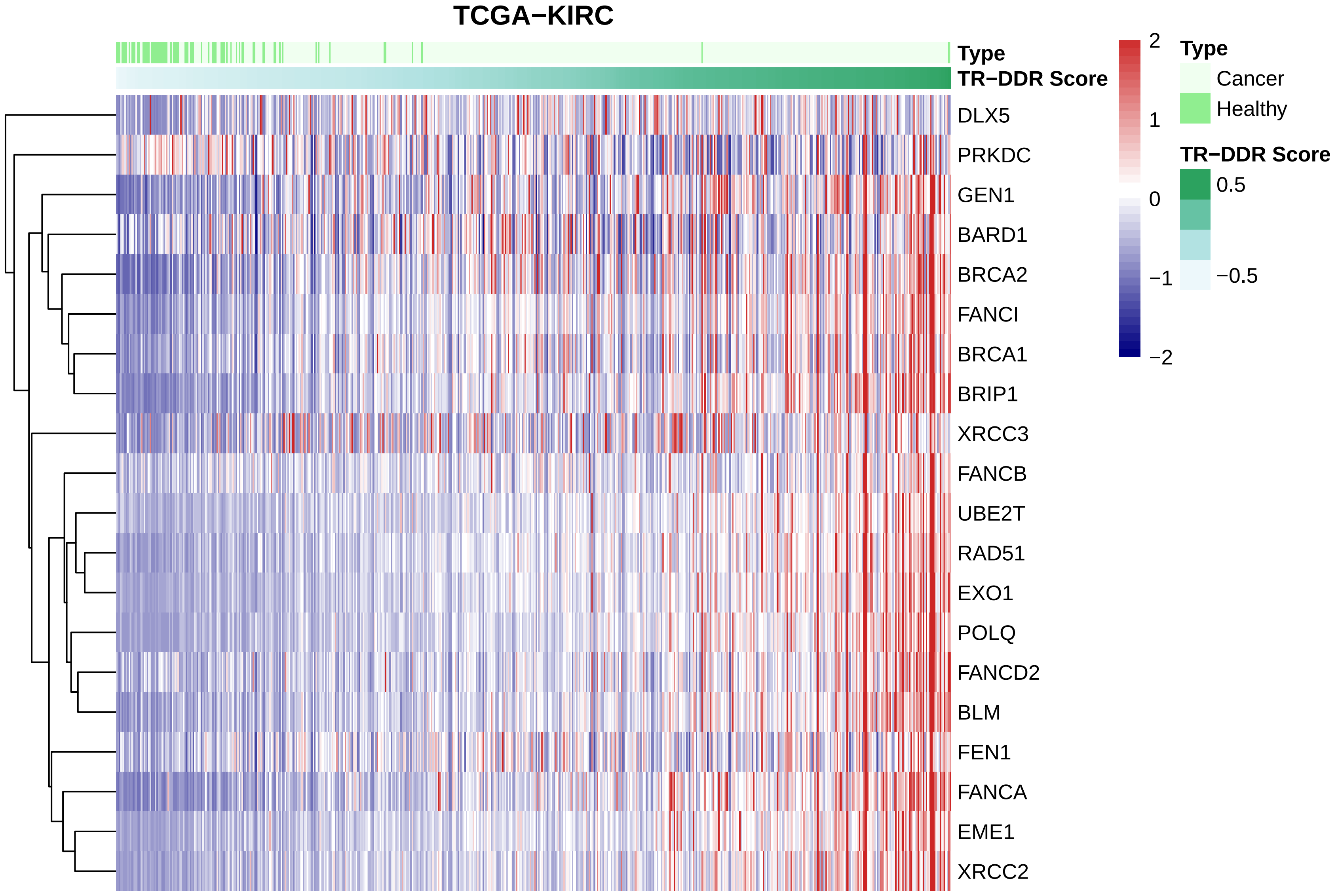

TCGA−KIRC
Type
TR−DDR Score
DLX5
PRKDC
GEN1
BARD1
BRCA2
FANCI
BRCA1
BRIP1
XRCC3
FANCB
UBE2T
RAD51
EXO1
POLQ
FANCD2
BLM
FEN1
FANCA
EME1
XRCC2
2
1
0
−1
−2
Type
Cancer
Healthy
TR−DDR Score
0.5
−0.5

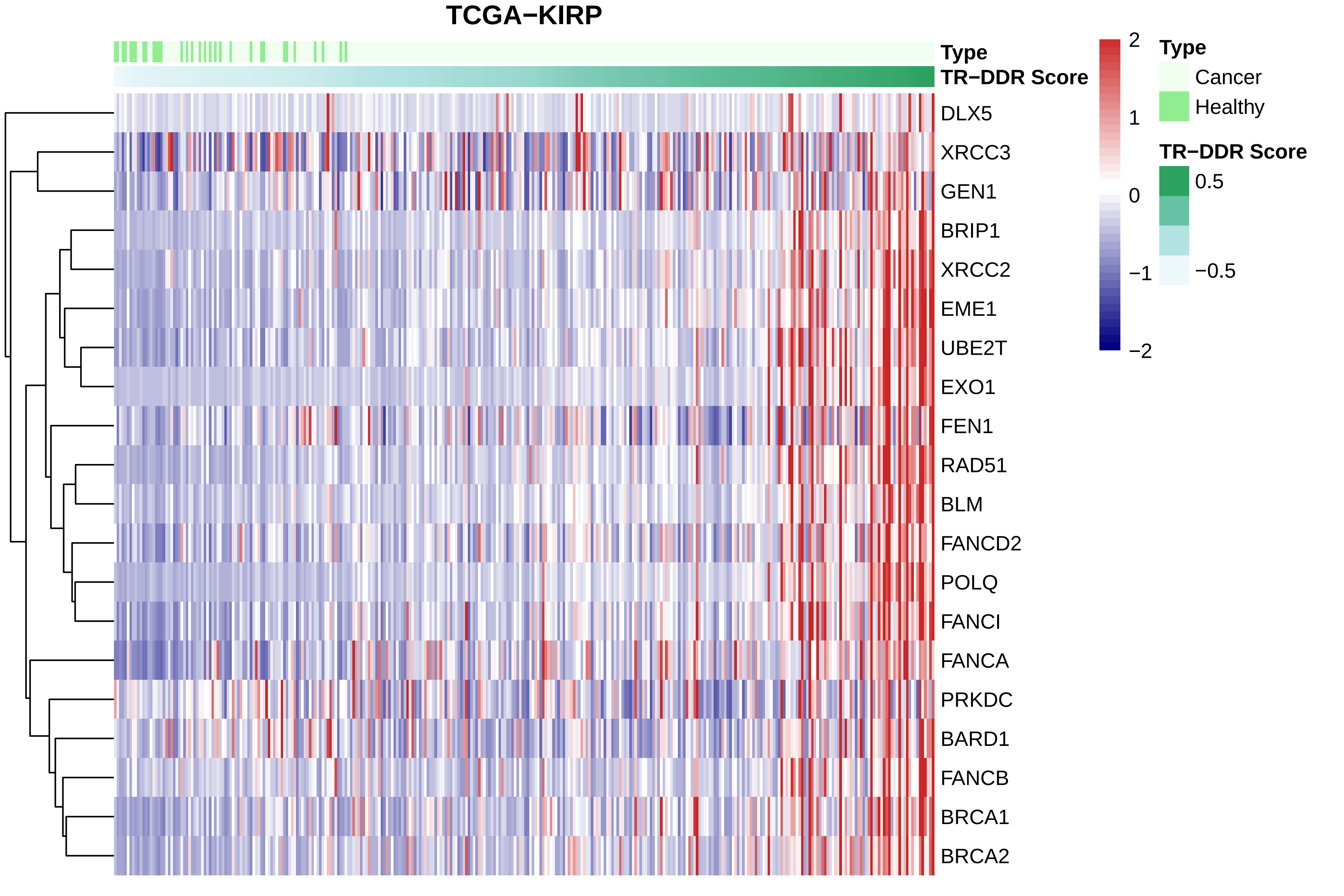
TCGA−KIRP
Type
TR−DDR Score
DLX5
XRCC3
GEN1
BRIP1
XRCC2
EME1
UBE2T
EXO1
FEN1
RAD51
BLM
FANCD2
POLQ
FANCI
FANCA
PRKDC
BARD1
FANCB
BRCA1
BRCA2
2
1
0
−1
−2
Type
Cancer
Healthy
TR−DDR Score
0.5
−0.5

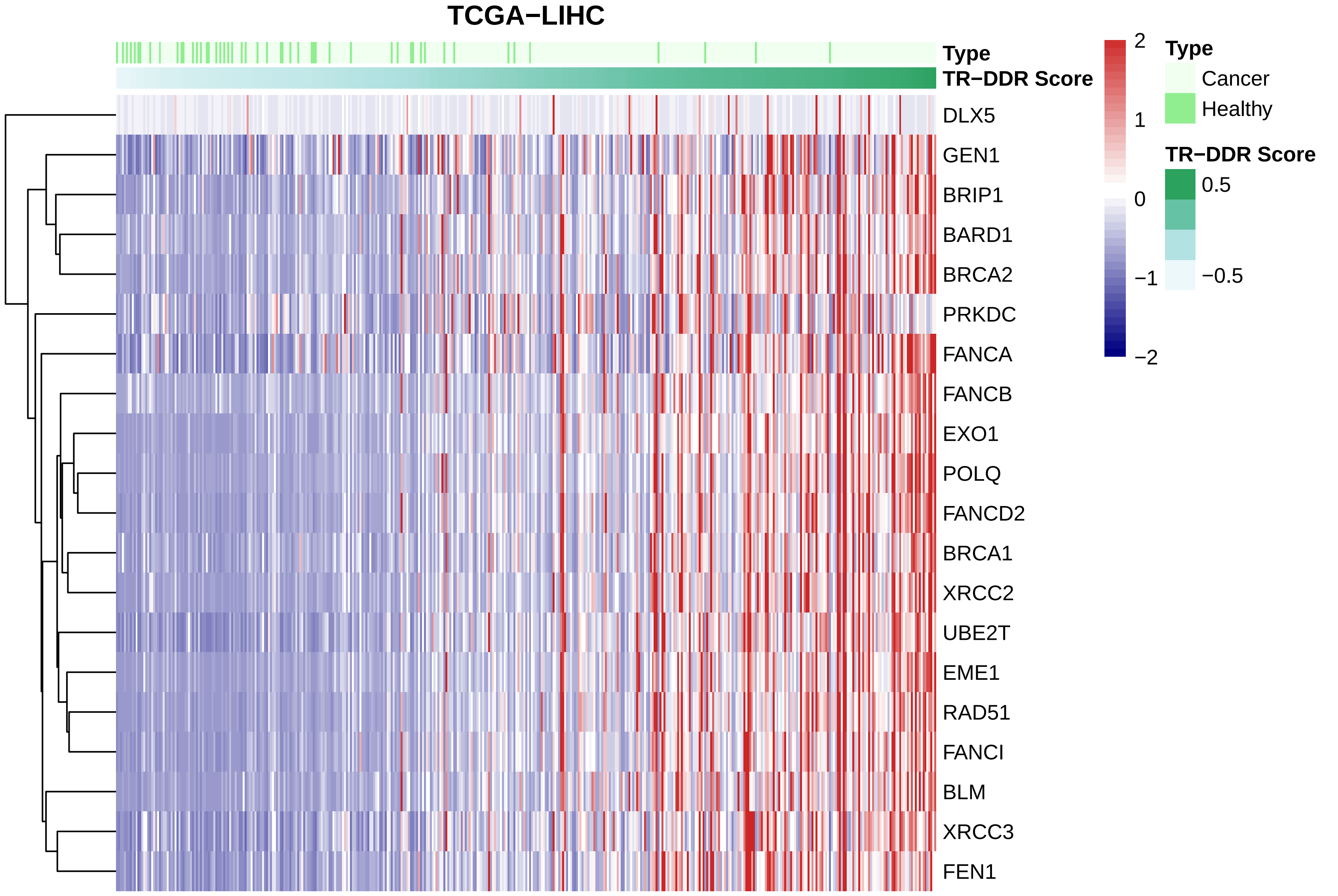
TCGA−LIHC
Type
TR−DDR Score
DLX5
GEN1
BRIP1
BARD1
BRCA2
PRKDC
FANCA
FANCB
EXO1
POLQ
FANCD2
BRCA1
XRCC2
UBE2T
EME1
RAD51
FANCI
BLM
XRCC3
FEN1
2
1
0
−1
−2
Type
Cancer
Healthy
TR−DDR Score
0.5
−0.5

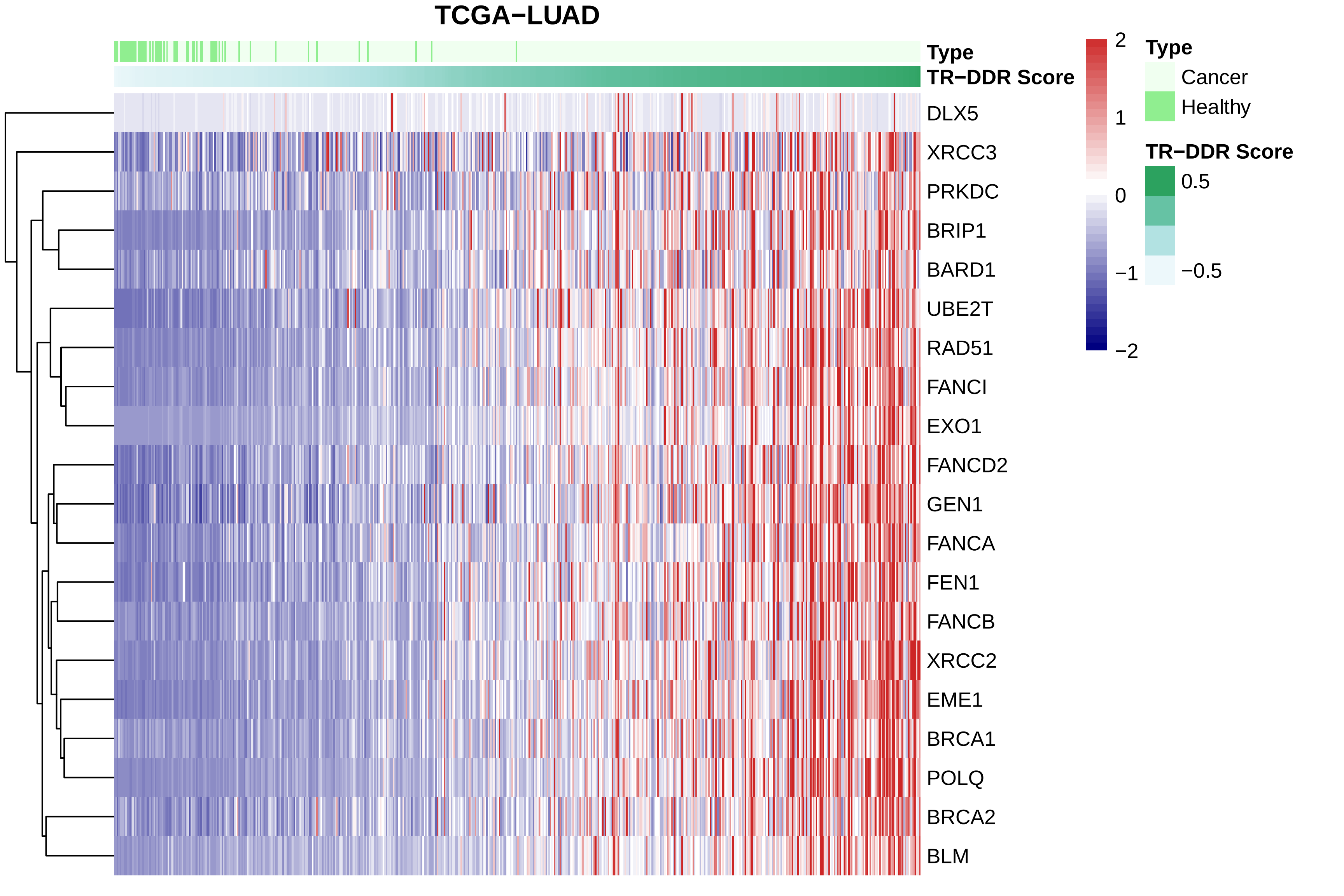
TCGA−LUAD
Type
TR−DDR Score
DLX5
XRCC3
PRKDC
BRIP1
BARD1
UBE2T
RAD51
FANCI
EXO1
FANCD2
GEN1
FANCA
FEN1
FANCB
XRCC2
EME1
BRCA1
POLQ
BRCA2
BLM
2
1
0
−1
−2
Type
Cancer
Healthy
TR−DDR Score
0.5
−0.5

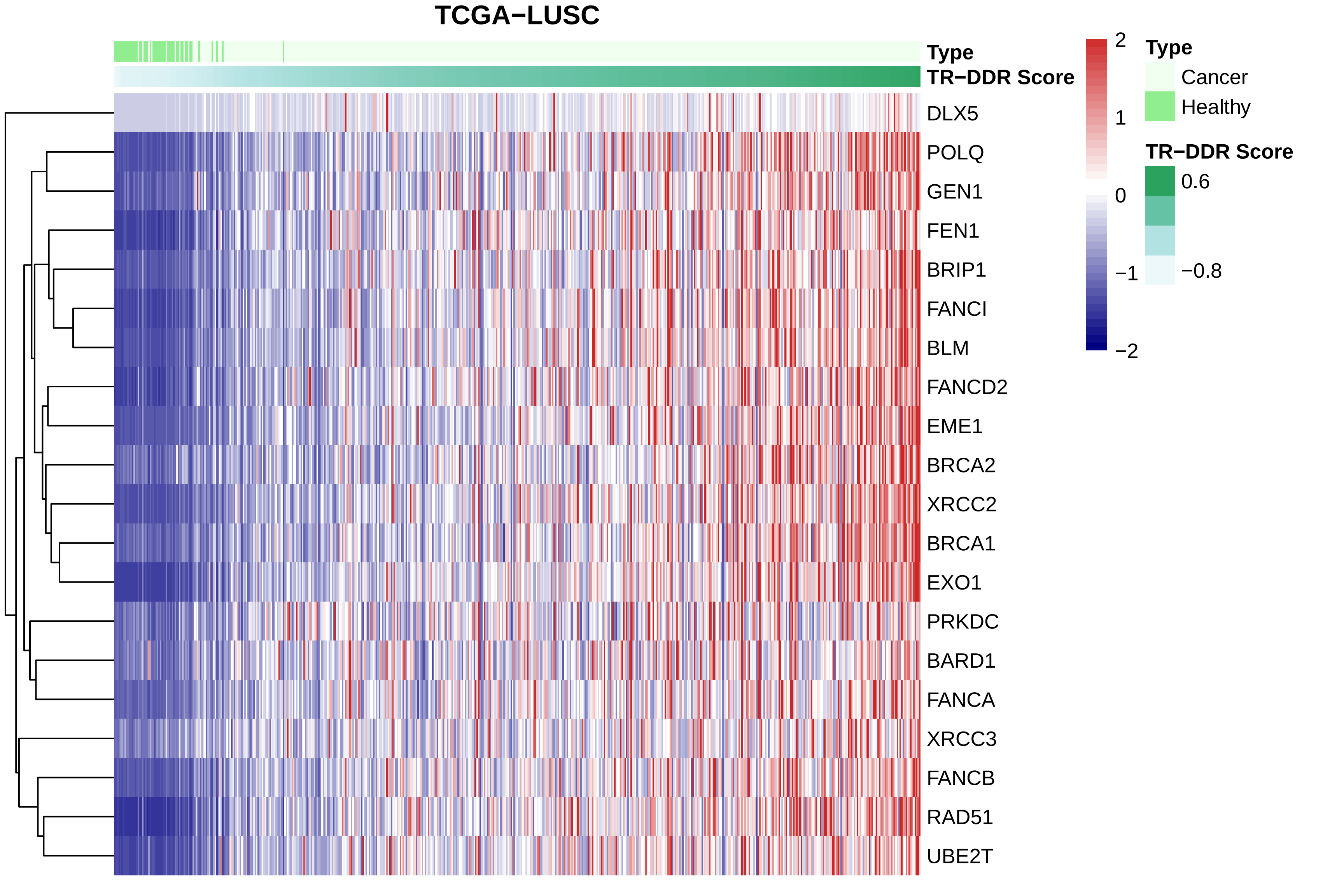
TCGA−LUSC
Type
TR−DDR Score
DLX5
POLQ
GEN1
FEN1
BRIP1
FANCI
BLM
FANCD2
EME1
BRCA2
XRCC2
BRCA1
EXO1
PRKDC
BARD1
FANCA
XRCC3
FANCB
RAD51
UBE2T
2
1
0
−1
−2
Type
Cancer
Healthy
TR−DDR Score
0.6
−0.8

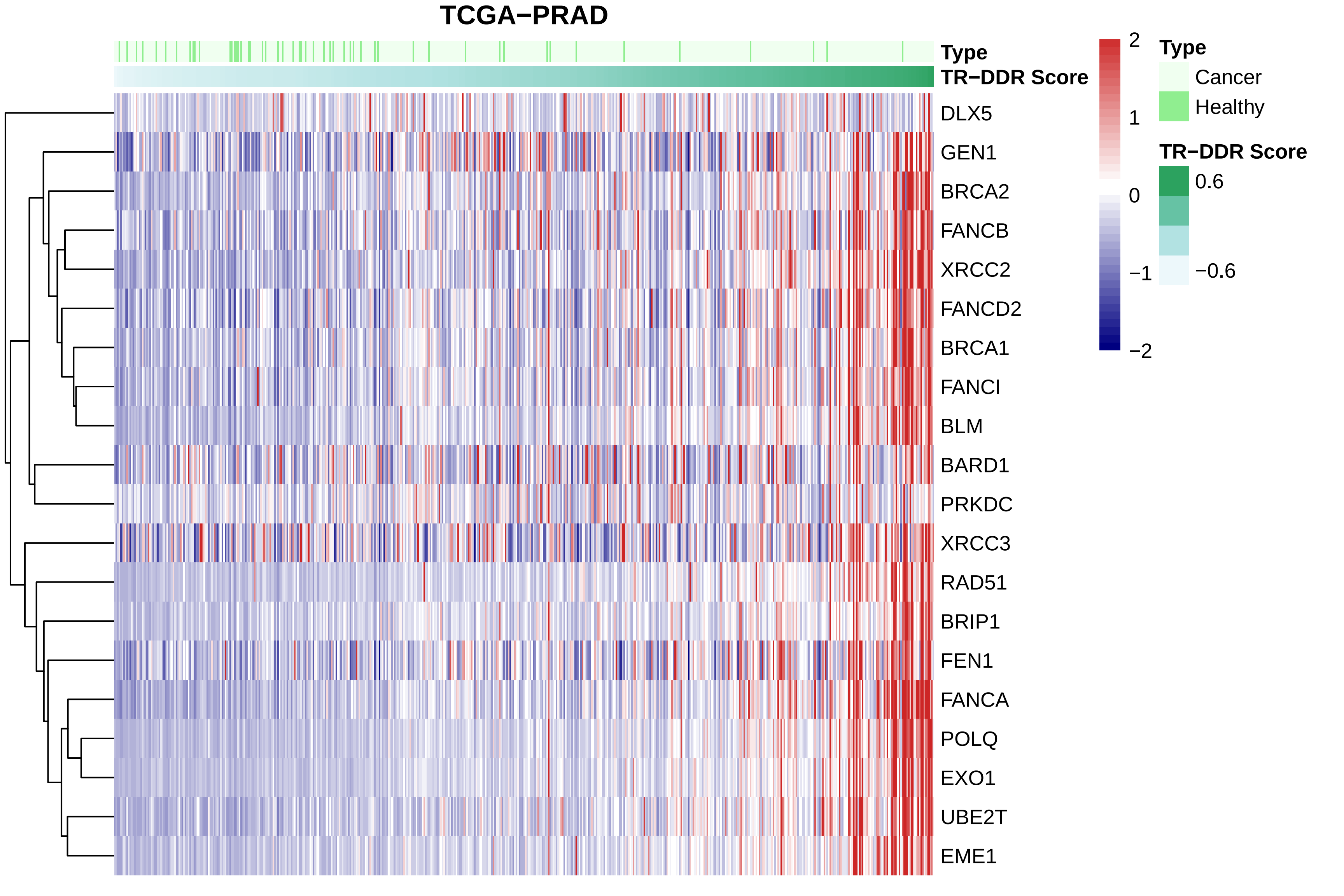

TCGA−PRAD
Type
TR−DDR Score
DLX5
GEN1
BRCA2
FANCB
XRCC2
FANCD2
BRCA1
FANCI
BLM
BARD1
PRKDC
XRCC3
RAD51
BRIP1
FEN1
FANCA
POLQ
EXO1
UBE2T
EME1
2
1
0
−1
−2
Type
Cancer
Healthy
TR−DDR Score
0.6
−0.6

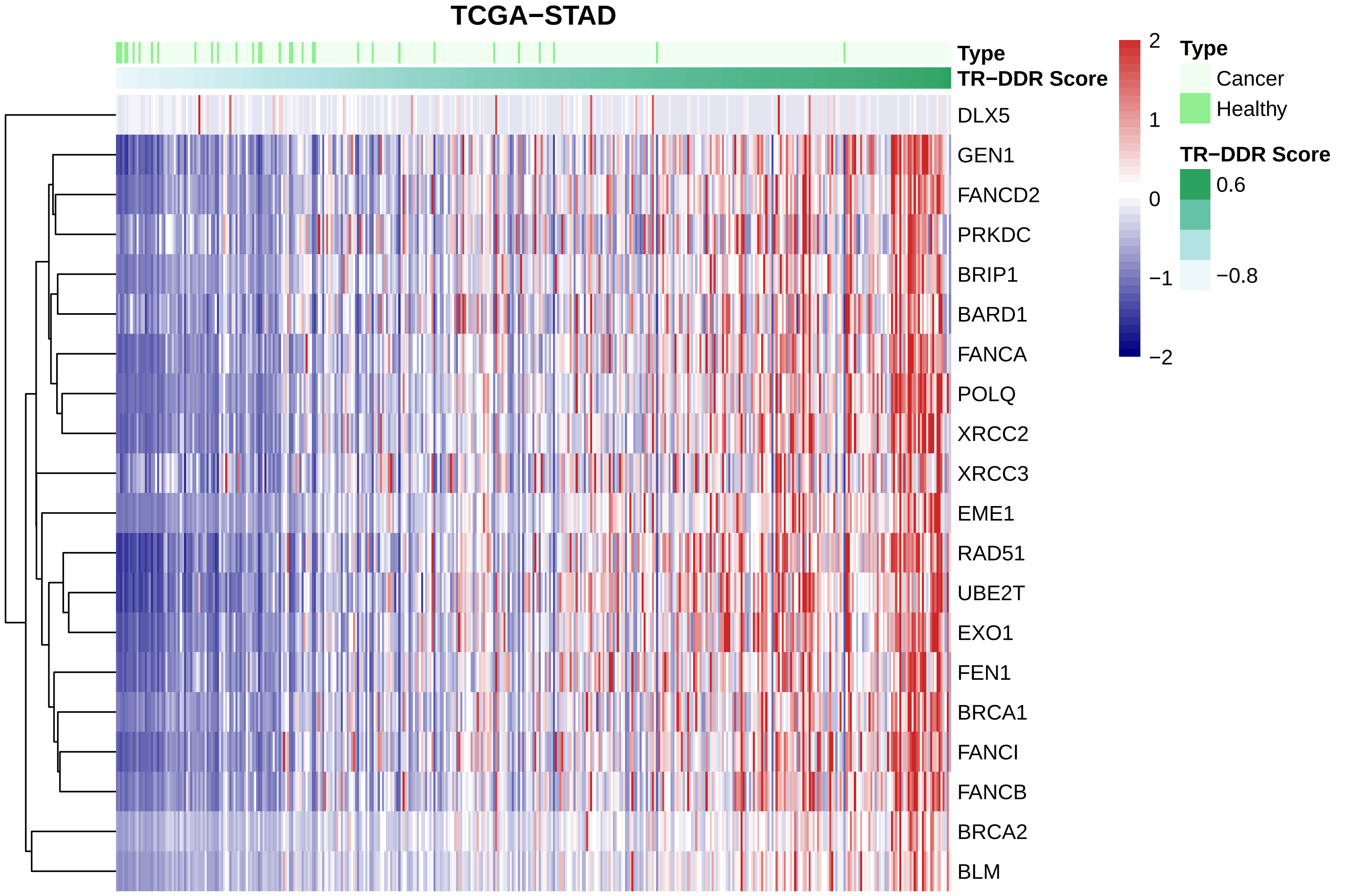
TCGA−STAD
Type
TR−DDR Score
DLX5
GEN1
FANCD2
PRKDC
BRIP1
BARD1
FANCA
POLQ
XRCC2
XRCC3
EME1
RAD51
UBE2T
EXO1
FEN1
BRCA1
FANCI
FANCB
BRCA2
BLM
2
1
0
−1
−2
Type
Cancer
Healthy
TR−DDR Score
0.6
−0.8

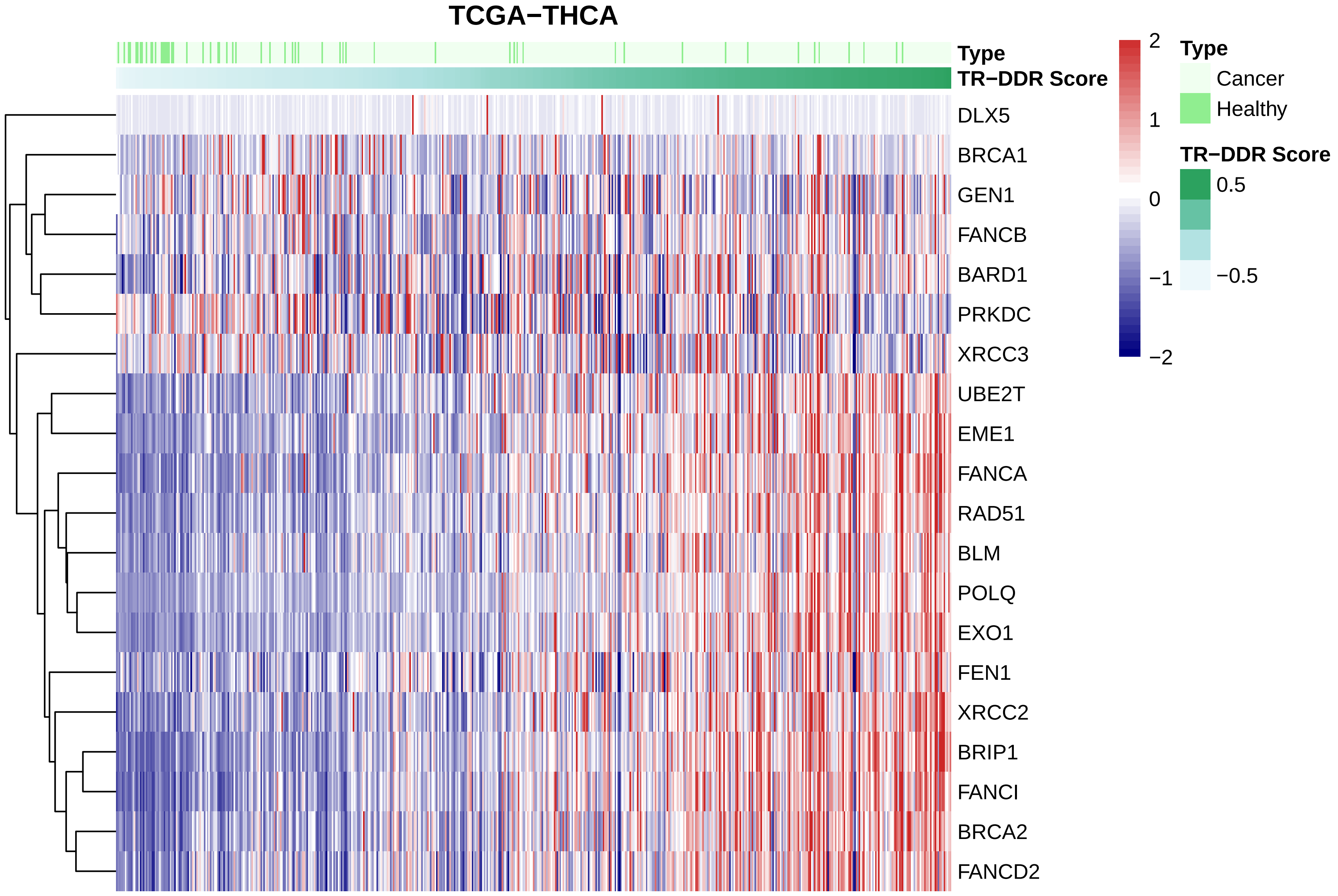
TCGA−THCA
Type
TR−DDR Score
DLX5
BRCA1
GEN1
FANCB
BARD1
PRKDC
XRCC3
UBE2T
EME1
FANCA
RAD51
BLM
POLQ
EXO1
FEN1
XRCC2
BRIP1
FANCI
BRCA2
FANCD2
2
1
0
−1
−2
Type
Cancer
Healthy
TR−DDR Score
0.5
−0.5
