# Supplementary material for: A score of DNA damage repair pathway with the predictive ability for chemotherapy and immunotherapy is strongly associated with immune signaling pathway in pan-cancer
Source: Front Immunol. 2022 Aug 23;13:943090. doi: 10.3389/fimmu.2022.943090 (PMC9445361; doi:10.3389/fimmu.2022.943090)
Supplement: Supplementary file 5 [file Image_5.pdf]

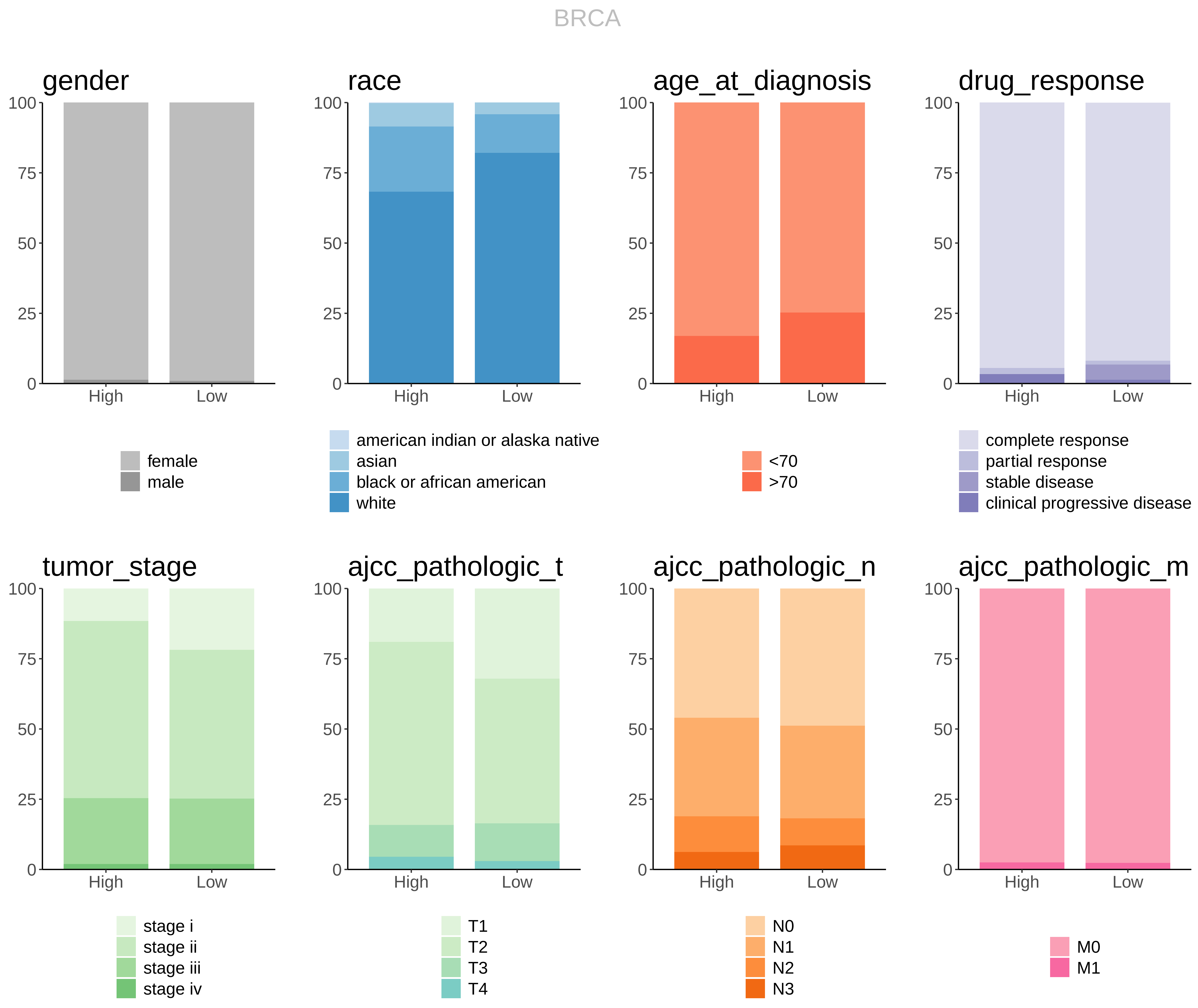
BRCA
gender
female
male
race
american indian or alaska native
asian
black or african american
white
age_at_diagnosis
<70
>70
drug_response
complete response
partial response
stable disease
clinical progressive disease
tumor_stage
stage i
stage ii
stage iii
stage iv
ajcc_pathologic_t
T1
T2
T3
T4
ajcc_pathologic_n
N0
N1
N2
N3
ajcc_pathologic_m
M0
M1
High
Low
0
25
50
75
100

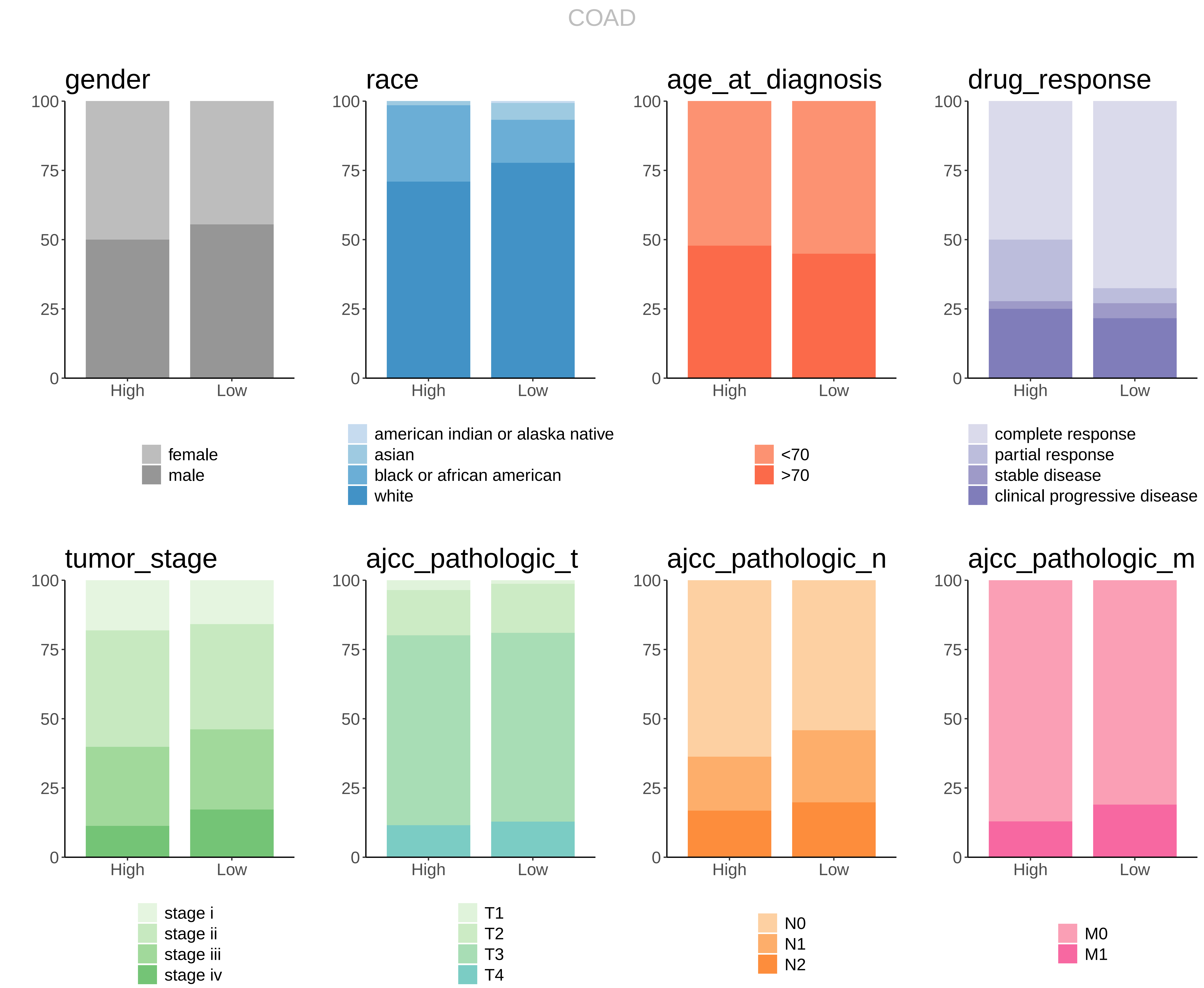
COAD
gender
100
75
50
25
0
High
Low
female
male
race
american indian or alaska native
asian
black or african american
white
age_at_diagnosis
<70
>70
drug_response
complete response
partial response
stable disease
clinical progressive disease
tumor_stage
stage i
stage ii
stage iii
stage iv
ajcc_pathologic_t
T1
T2
T3
T4
ajcc_pathologic_n
N0
N1
N2
ajcc_pathologic_m
M0
M1

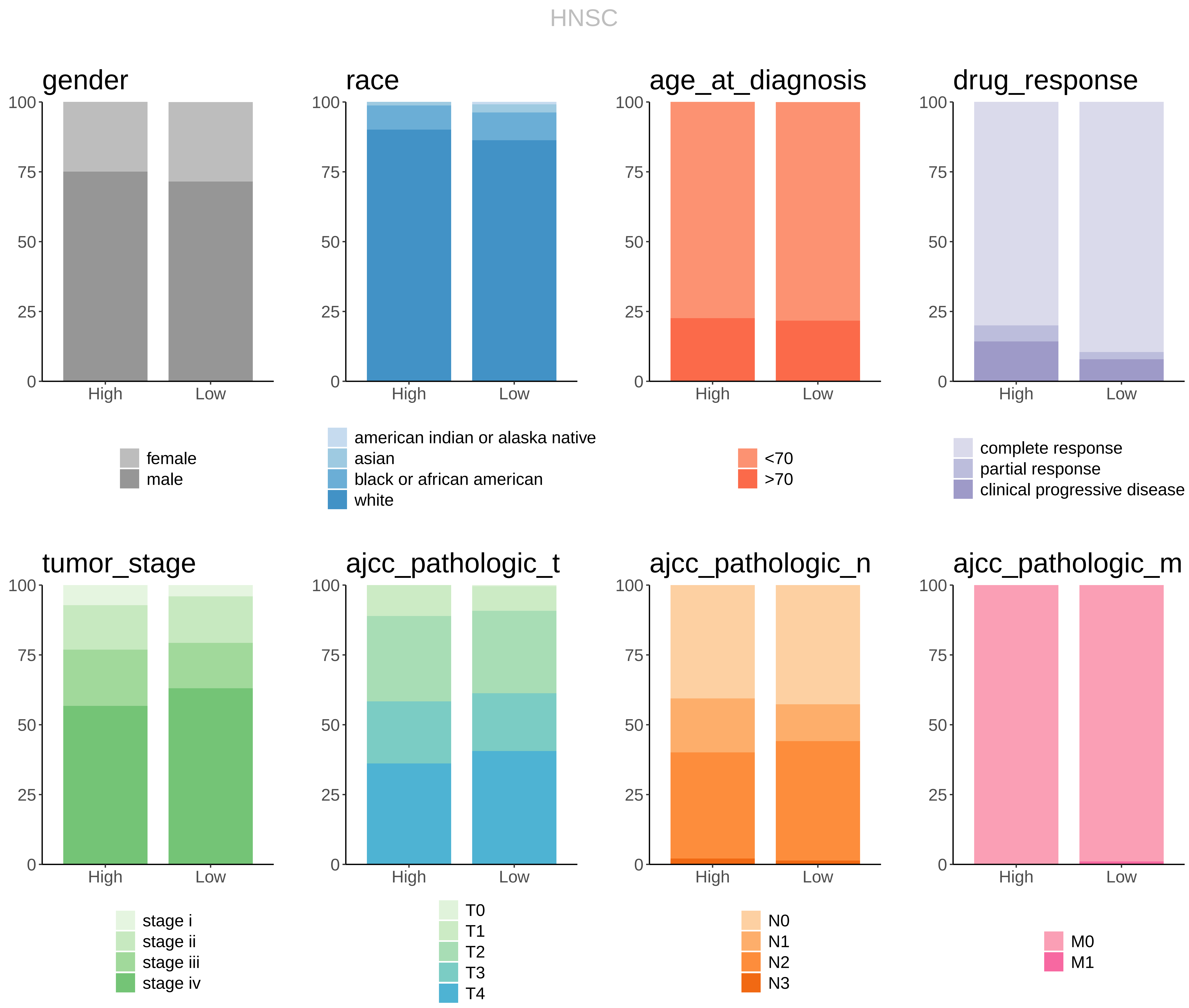
HNSC
gender
race
age_at_diagnosis
drug_response
100
75
50
25
0
High
Low
female
male
american indian or alaska native
asian
black or african american
white
<70
>70
complete response
partial response
clinical progressive disease
tumor_stage
ajcc_pathologic_t
ajcc_pathologic_n
ajcc_pathologic_m
stage i
stage ii
stage iii
stage iv
T0
T1
T2
T3
T4
N0
N1
N2
N3
M0
M1

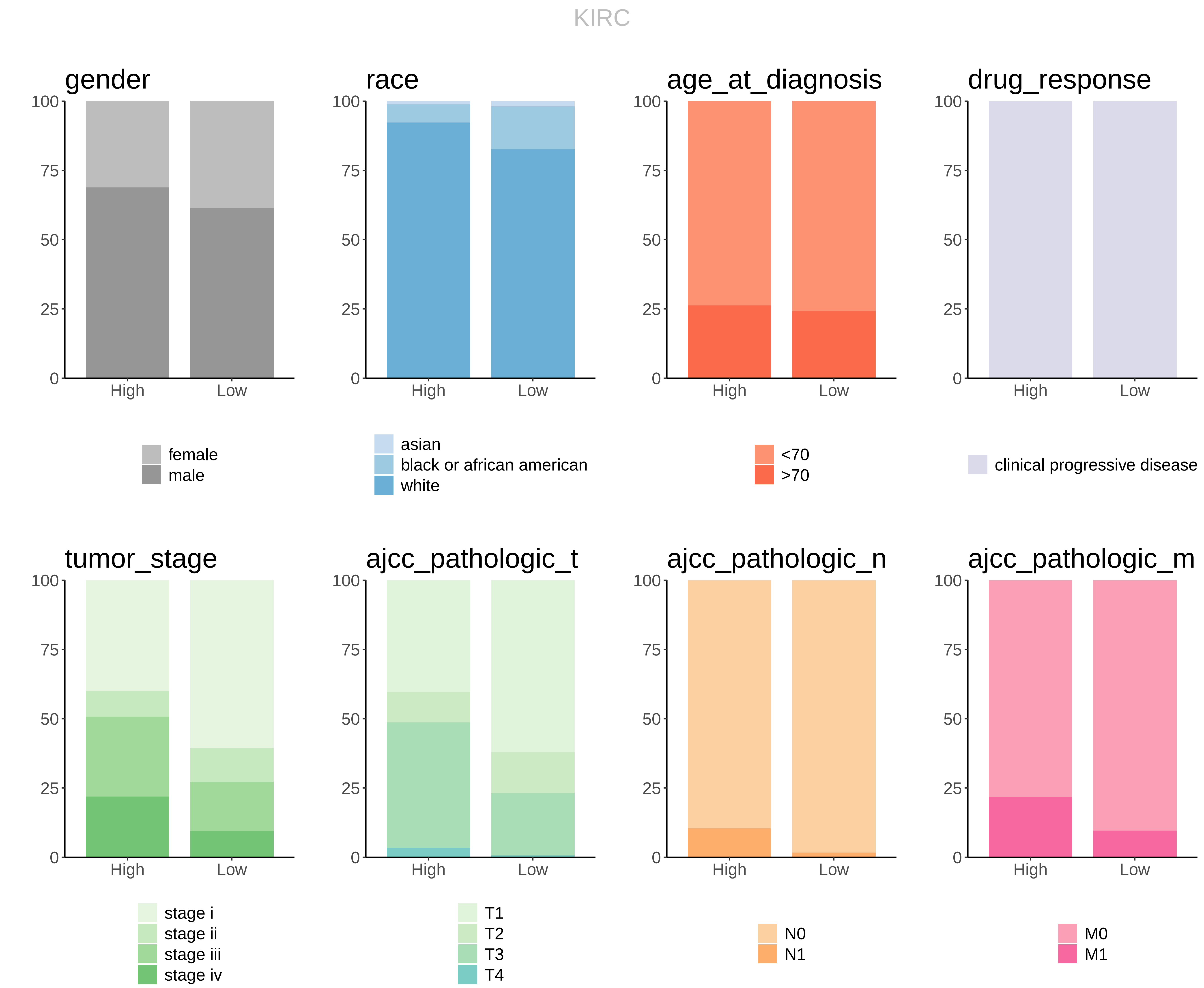

KIRC
gender
100
75
50
25
0
High
Low
female
male
race
asian
black or african american
white
age_at_diagnosis
<70
>70
drug_response
clinical progressive disease
tumor_stage
stage i
stage ii
stage iii
stage iv
ajcc_pathologic_t
T1
T2
T3
T4
ajcc_pathologic_n
N0
N1
ajcc_pathologic_m
M0
M1

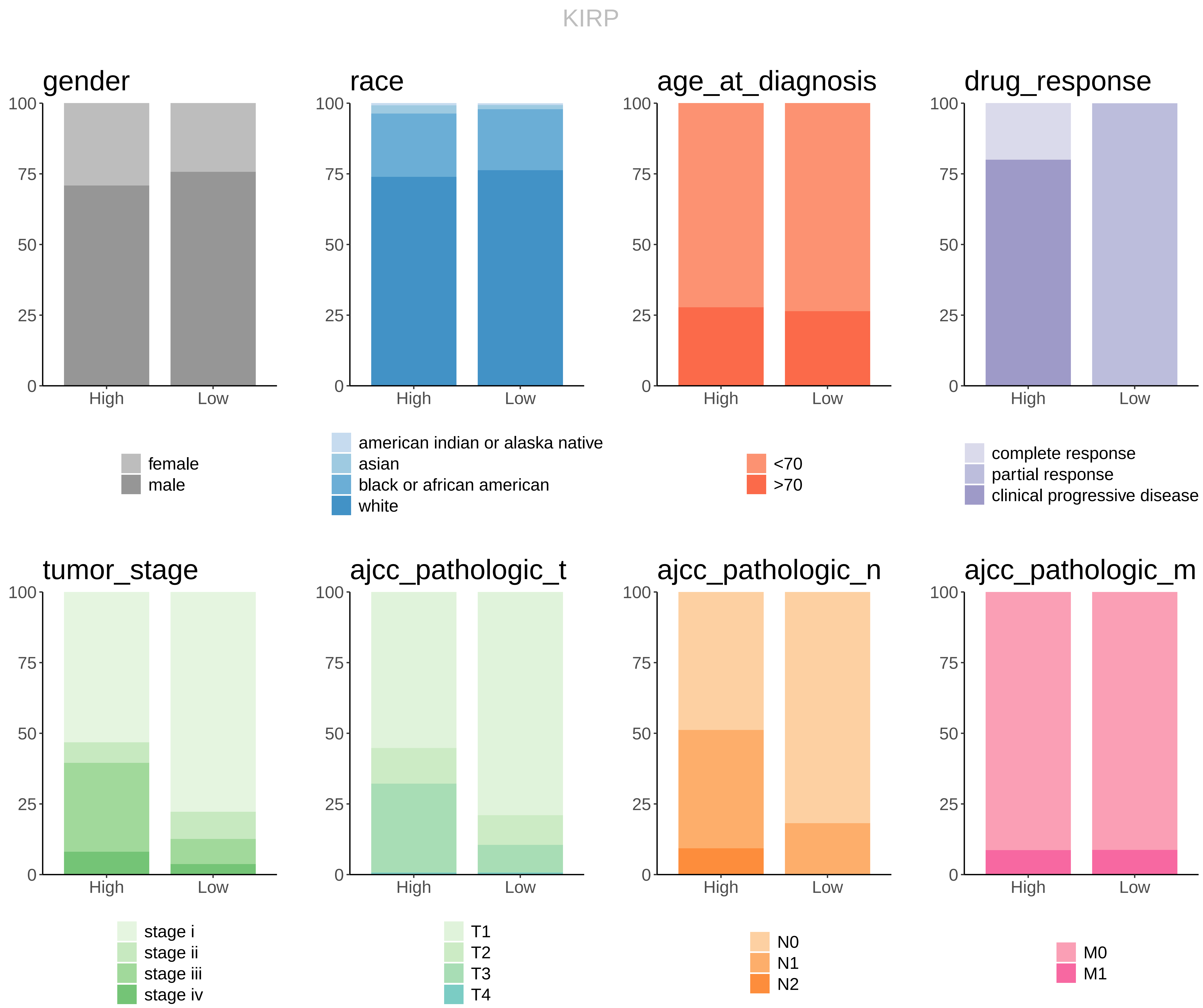
KIRP
gender
female
male
race
american indian or alaska native
asian
black or african american
white
age_at_diagnosis
<70
>70
drug_response
complete response
partial response
clinical progressive disease
tumor_stage
stage i
stage ii
stage iii
stage iv
ajcc_pathologic_t
T1
T2
T3
T4
ajcc_pathologic_n
N0
N1
N2
ajcc_pathologic_m
M0
M1
High
Low
0
25
50
75
100

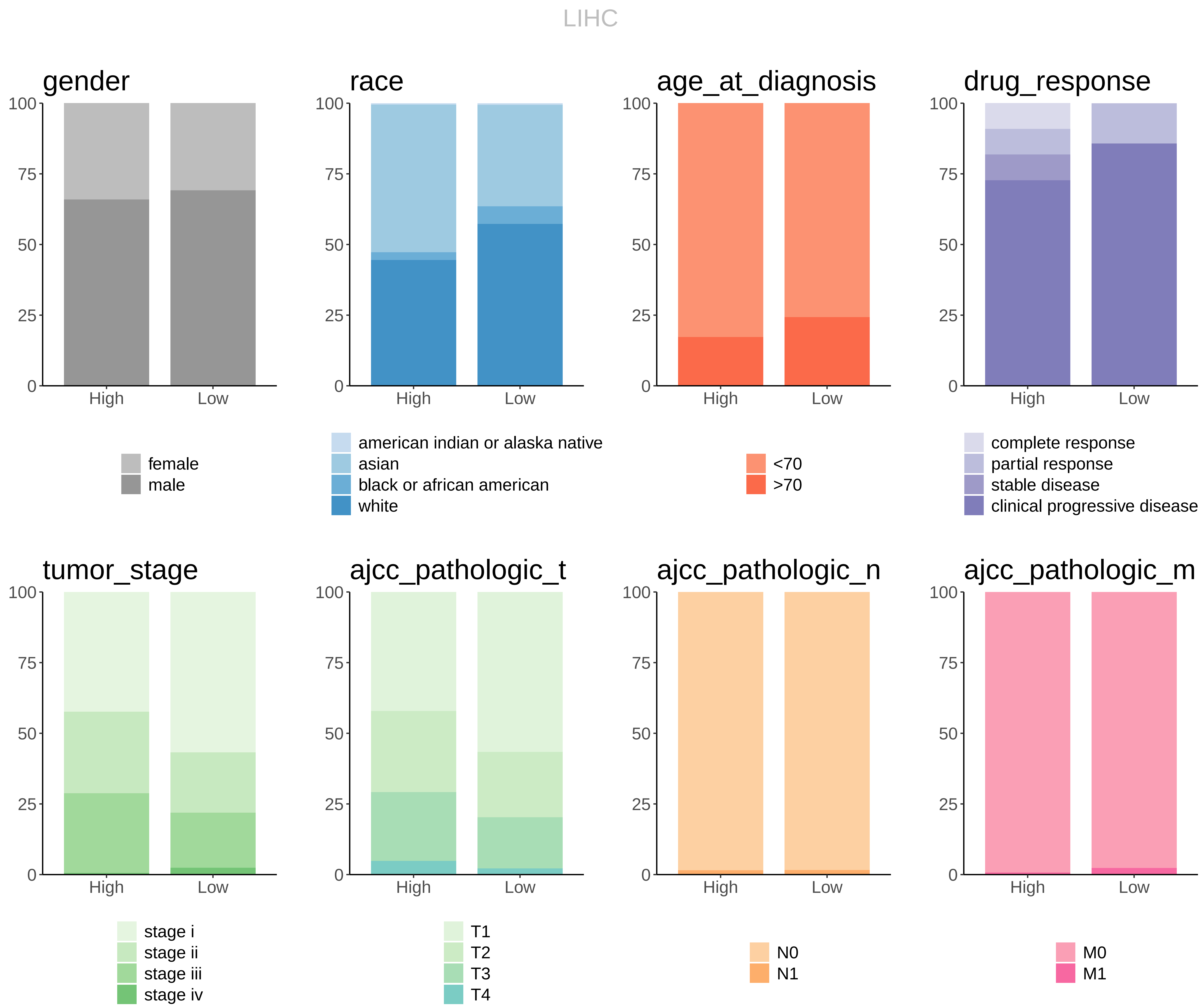
LIHC
gender
race
age_at_diagnosis
drug_response
100
75
50
25
0
High
Low
female
male
american indian or alaska native
asian
black or african american
white
<70
>70
complete response
partial response
stable disease
clinical progressive disease
tumor_stage
ajcc_pathologic_t
ajcc_pathologic_n
ajcc_pathologic_m
stage i
stage ii
stage iii
stage iv
T1
T2
T3
T4
N0
N1
M0
M1

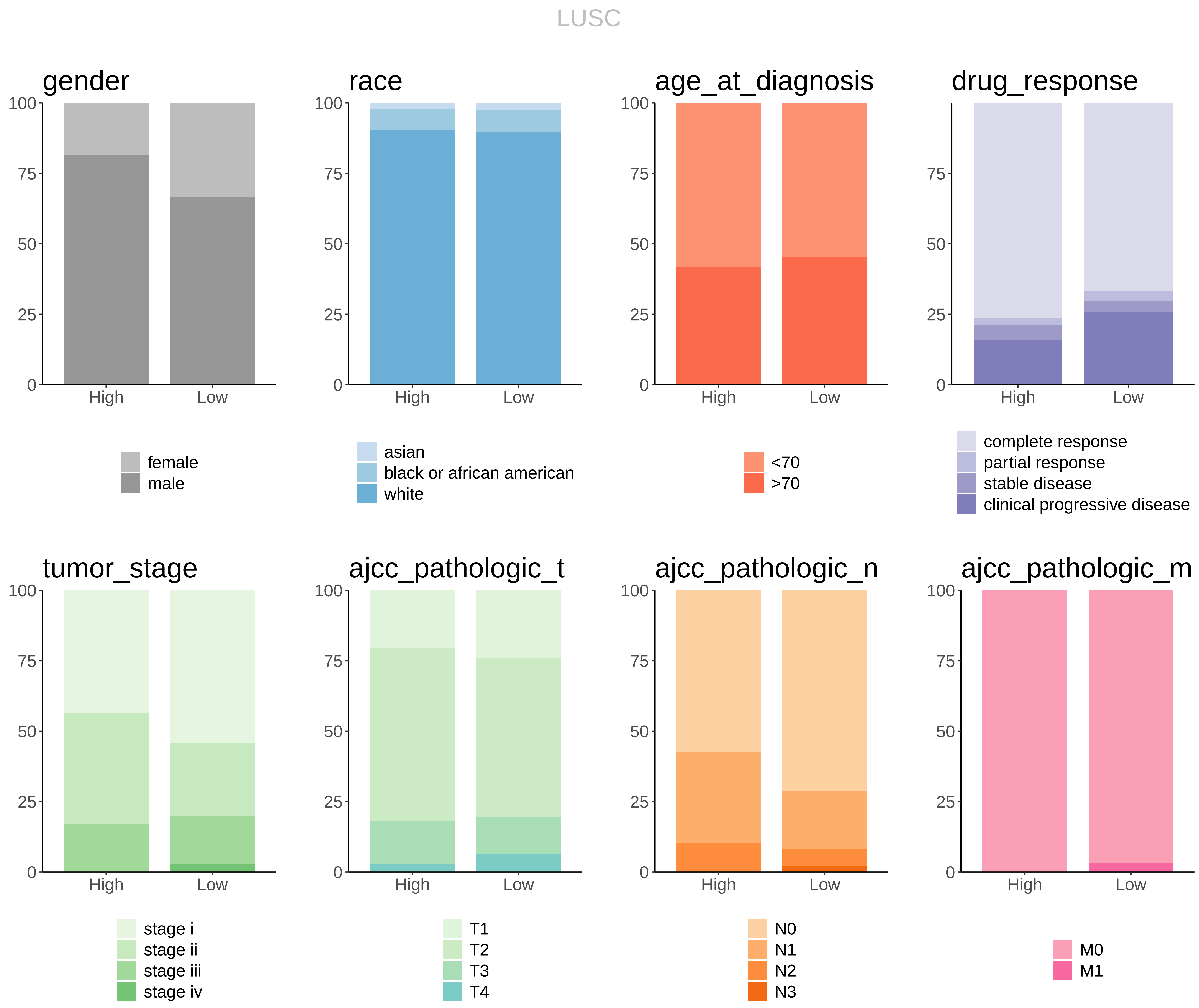
LUSC
gender
race
age_at_diagnosis
drug_response
tumor_stage
ajcc_pathologic_t
ajcc_pathologic_n
ajcc_pathologic_m
100
75
50
25
0
High
Low
female
male
asian
black or african american
white
<70
>70
complete response
partial response
stable disease
clinical progressive disease
stage i
stage ii
stage iii
stage iv
T1
T2
T3
T4
N0
N1
N2
N3
M0
M1

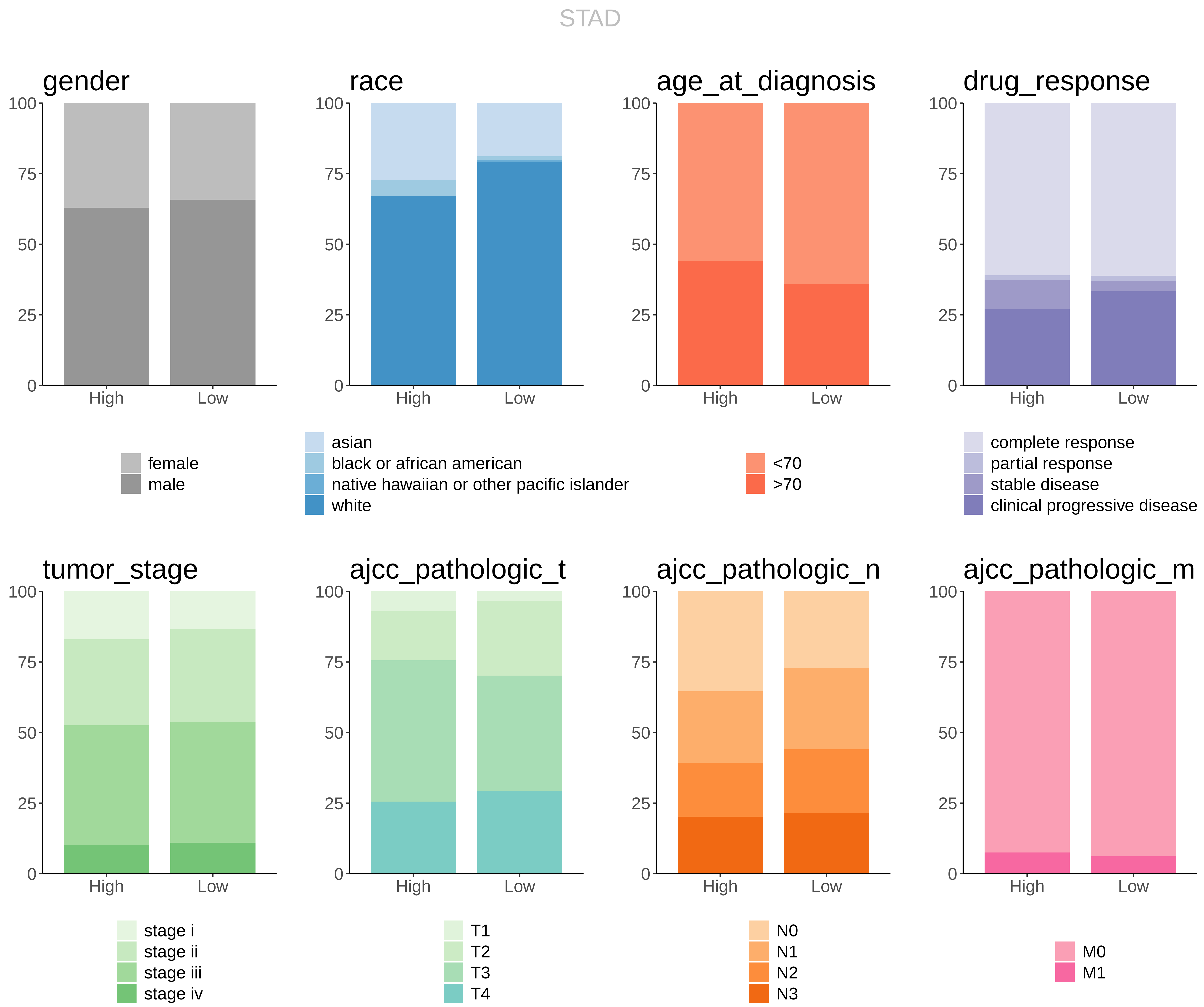
STAD
gender
race
age_at_diagnosis
drug_response
100
75
50
25
0
High
Low
female
male
asian
black or african american
native hawaiian or other pacific islander
white
<70
>70
complete response
partial response
stable disease
clinical progressive disease
tumor_stage
ajcc_pathologic_t
ajcc_pathologic_n
ajcc_pathologic_m
stage i
stage ii
stage iii
stage iv
T1
T2
T3
T4
N0
N1
N2
N3
M0
M1

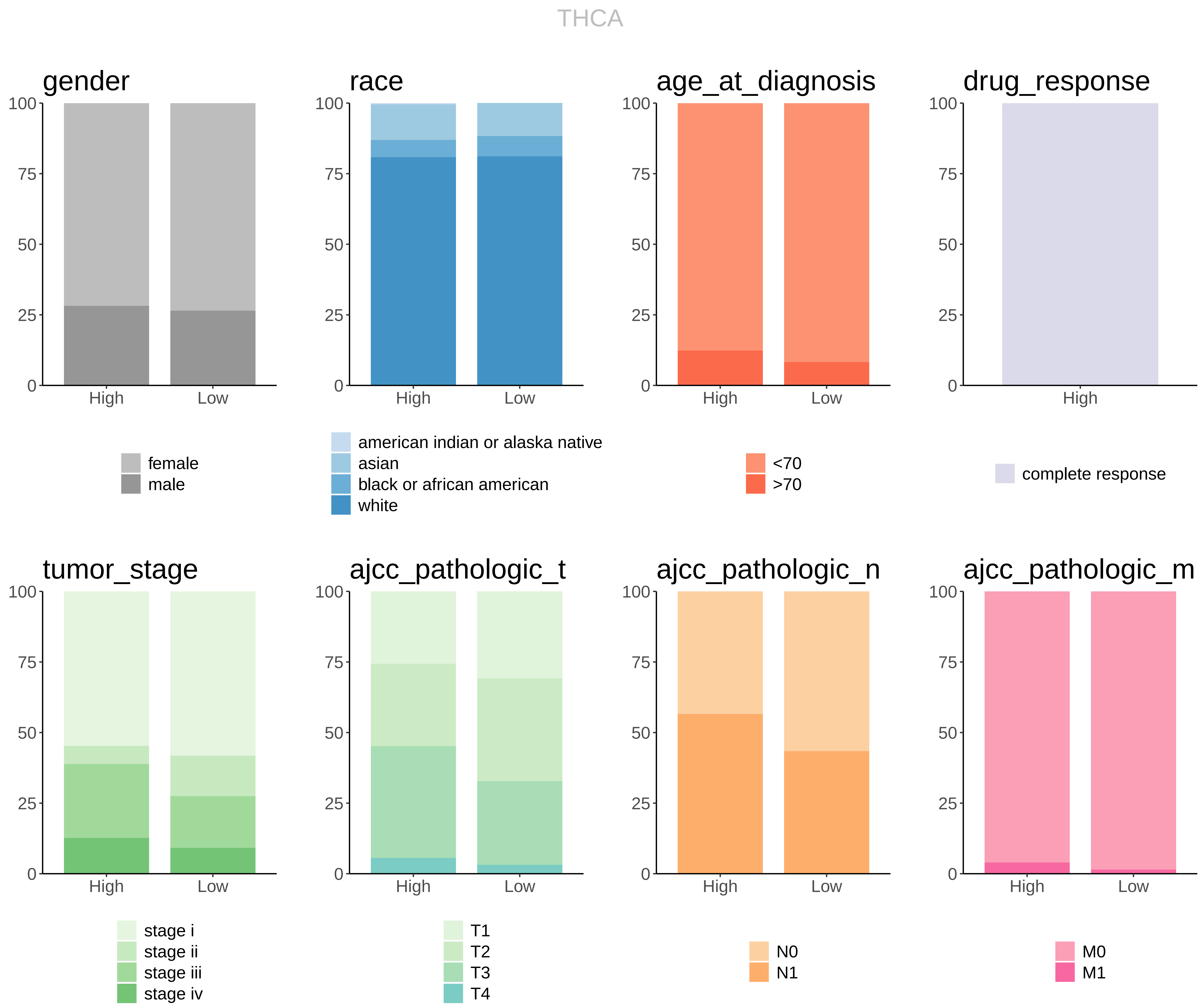
THCA
gender
race
age_at_diagnosis
drug_response
High
Low
female
male
american indian or alaska native
asian
black or african american
white
<70
>70
complete response
tumor_stage
ajcc_pathologic_t
ajcc_pathologic_n
ajcc_pathologic_m
stage i
stage ii
stage iii
stage iv
T1
T2
T3
T4
N0
N1
M0
M1
0
25
50
75
100
