# Supplementary material for: A score of DNA damage repair pathway with the predictive ability for chemotherapy and immunotherapy is strongly associated with immune signaling pathway in pan-cancer
Source: Front Immunol. 2022 Aug 23;13:943090. doi: 10.3389/fimmu.2022.943090 (PMC9445361; doi:10.3389/fimmu.2022.943090)
Supplement: Supplementary file 7 [file Image_7.pdf]

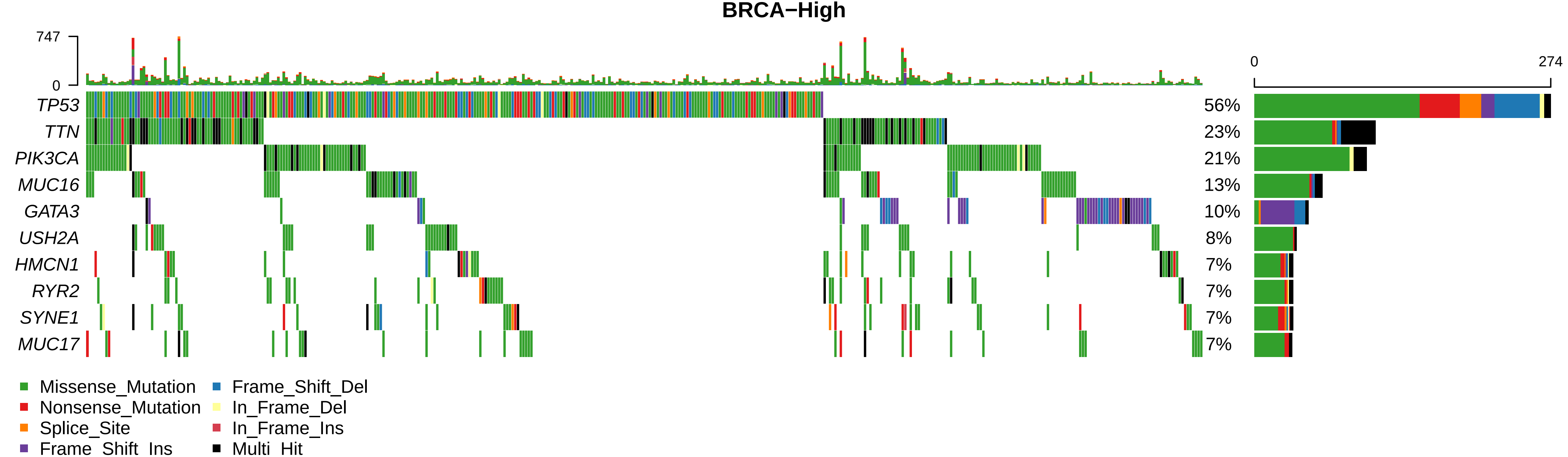
BRCA−High
747
0
0
274
TP53
TTN
PIK3CA
MUC16
GATA3
USH2A
HMCN1
RYR2
SYNE1
MUC17
56%
23%
21%
13%
10%
8%
7%
7%
7%
7%
Missense_Mutation
Nonsense_Mutation
Splice_Site
Frame_Shift_Ins
Frame_Shift_Del
In_Frame_Del
In_Frame_Ins
Multi_Hit

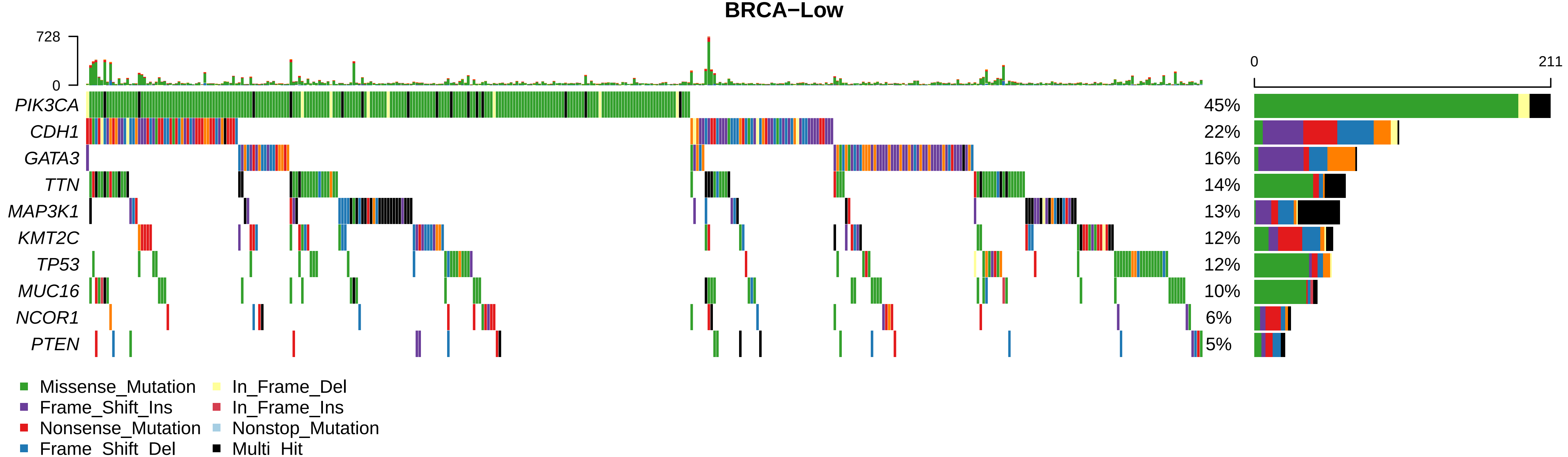
BRCA−Low
728
0
0
211
PIK3CA
CDH1
GATA3
TTN
MAP3K1
KMT2C
TP53
MUC16
NCOR1
PTEN
45%
22%
16%
14%
13%
12%
12%
10%
6%
5%
Missense_Mutation
Frame_Shift_Ins
Nonsense_Mutation
Frame_Shift_Del
In_Frame_Del
In_Frame_Ins
Nonstop_Mutation
Multi_Hit

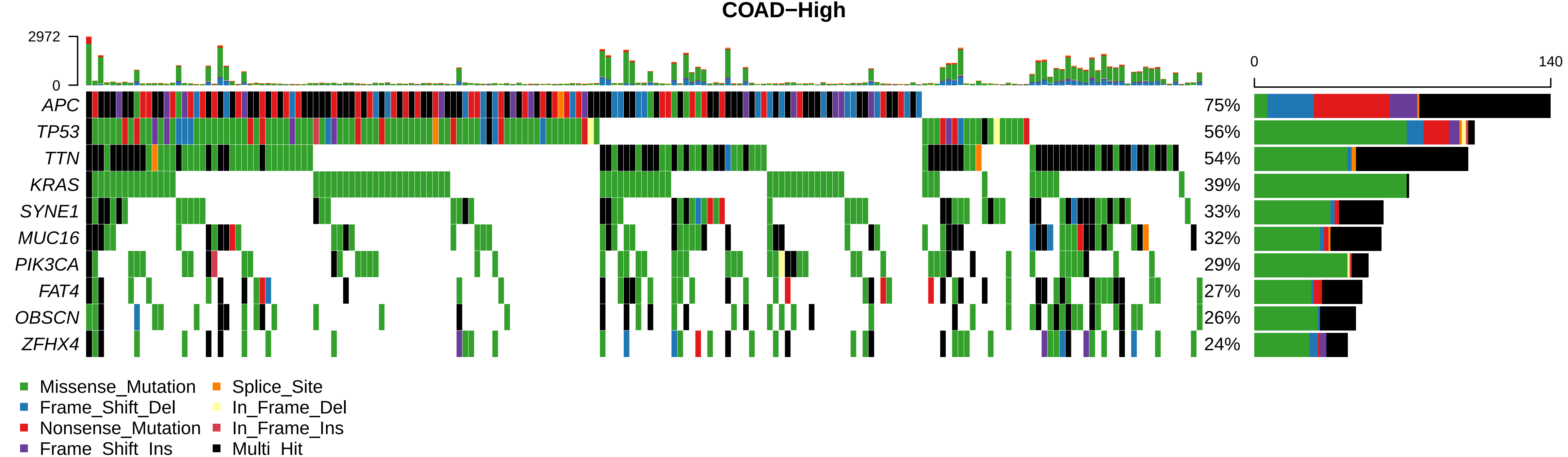
COAD−High
2972
0
0
140
APC
TP53
TTN
KRAS
SYNE1
MUC16
PIK3CA
FAT4
OBSCN
ZFHX4
75%
56%
54%
39%
33%
32%
29%
27%
26%
24%
Missense_Mutation
Frame_Shift_Del
Nonsense_Mutation
Frame_Shift_Ins
Splice_Site
In_Frame_Del
In_Frame_Ins
Multi_Hit

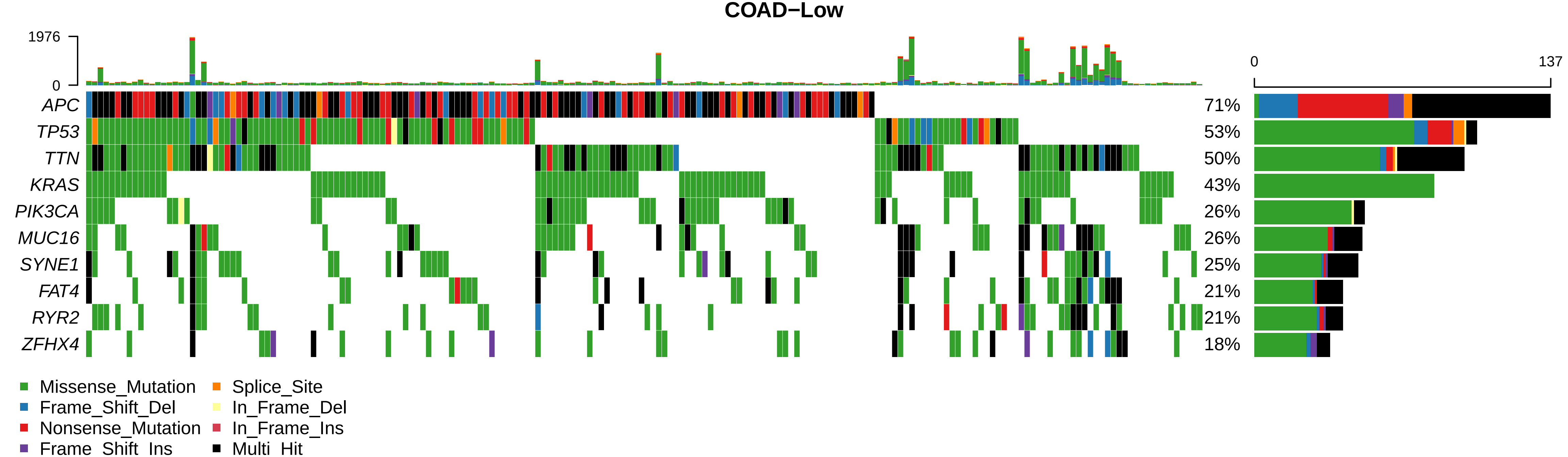
COAD−Low
1976
0
0
137
APC
TP53
TTN
KRAS
PIK3CA
MUC16
SYNE1
FAT4
RYR2
ZFHX4
71%
53%
50%
43%
26%
26%
25%
21%
21%
18%
Missense_Mutation
Frame_Shift_Del
Nonsense_Mutation
Frame_Shift_Ins
Splice_Site
In_Frame_Del
In_Frame_Ins
Multi_Hit

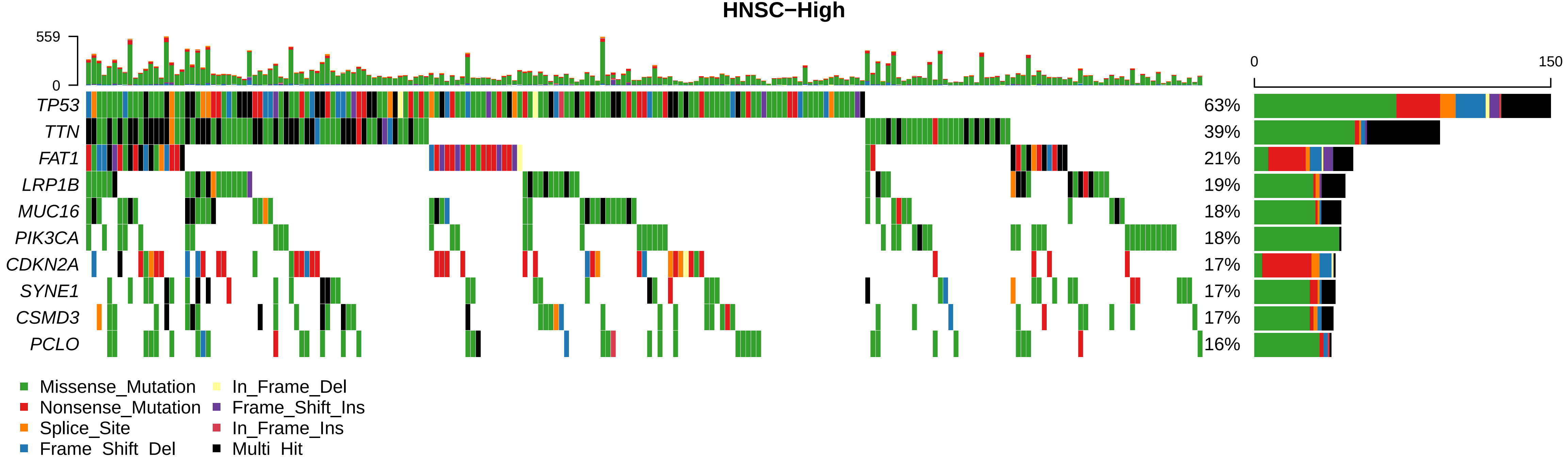
HNSC−High
559
0
TP53
TTN
FAT1
LRP1B
MUC16
PIK3CA
CDKN2A
SYNE1
CSMD3
PCLO
63%
39%
21%
19%
18%
18%
17%
17%
17%
16%
0
150
Missense_Mutation
Nonsense_Mutation
Splice_Site
Frame_Shift_Del
In_Frame_Del
Frame_Shift_Ins
In_Frame_Ins
Multi_Hit

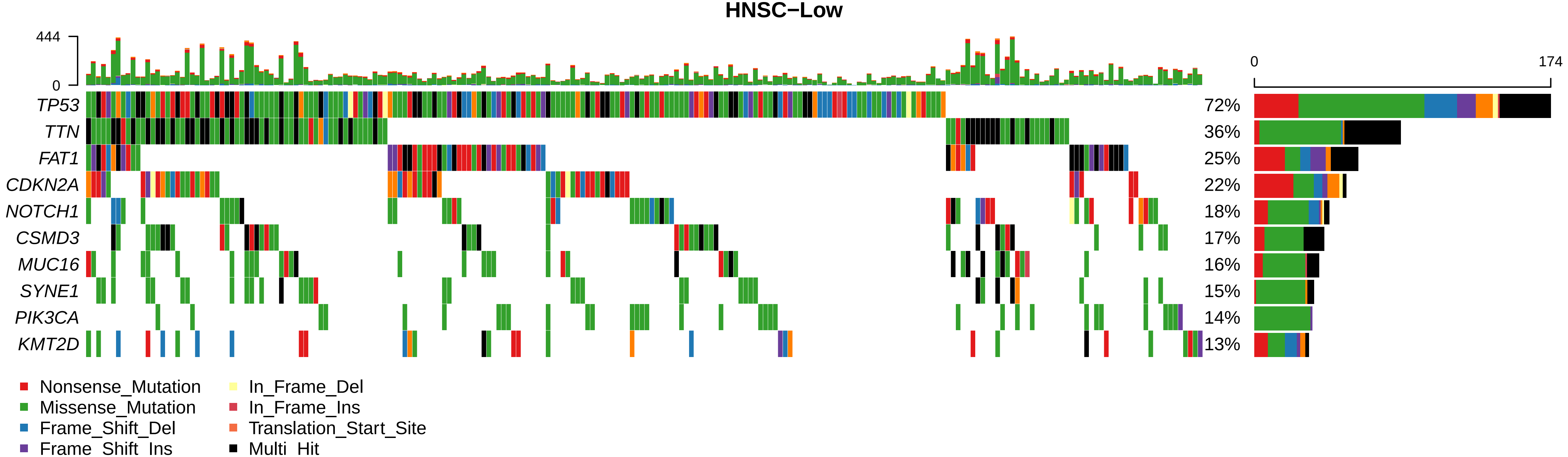

HNSC−Low
444
0
0
174
TP53
TTN
FAT1
CDKN2A
NOTCH1
CSMD3
MUC16
SYNE1
PIK3CA
KMT2D
72%
36%
25%
22%
18%
17%
16%
15%
14%
13%
Nonsense_Mutation
Missense_Mutation
Frame_Shift_Del
Frame_Shift_Ins
In_Frame_Del
In_Frame_Ins
Translation_Start_Site
Multi_Hit

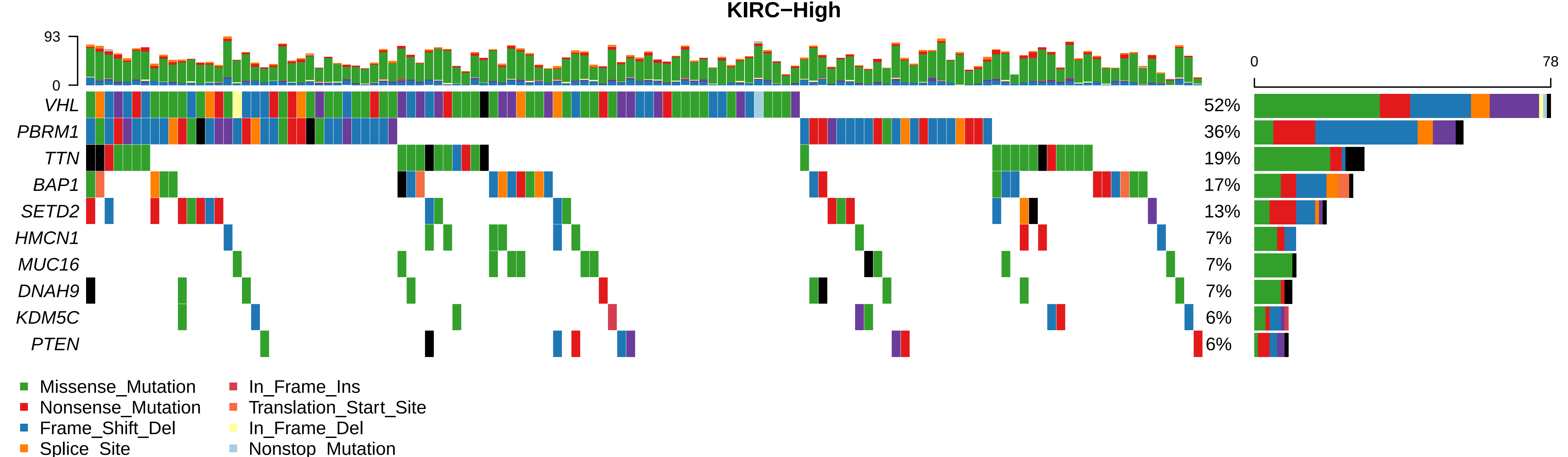
KIRC−High
93
0
VHL
PBRM1
TTN
BAP1
SETD2
HMCN1
MUC16
DNAH9
KDM5C
PTEN
52%
36%
19%
17%
13%
7%
7%
7%
6%
6%
0
78
Missense_Mutation
Nonsense_Mutation
Frame_Shift_Del
Splice_Site
In_Frame_Ins
Translation_Start_Site
In_Frame_Del
Nonstop_Mutation

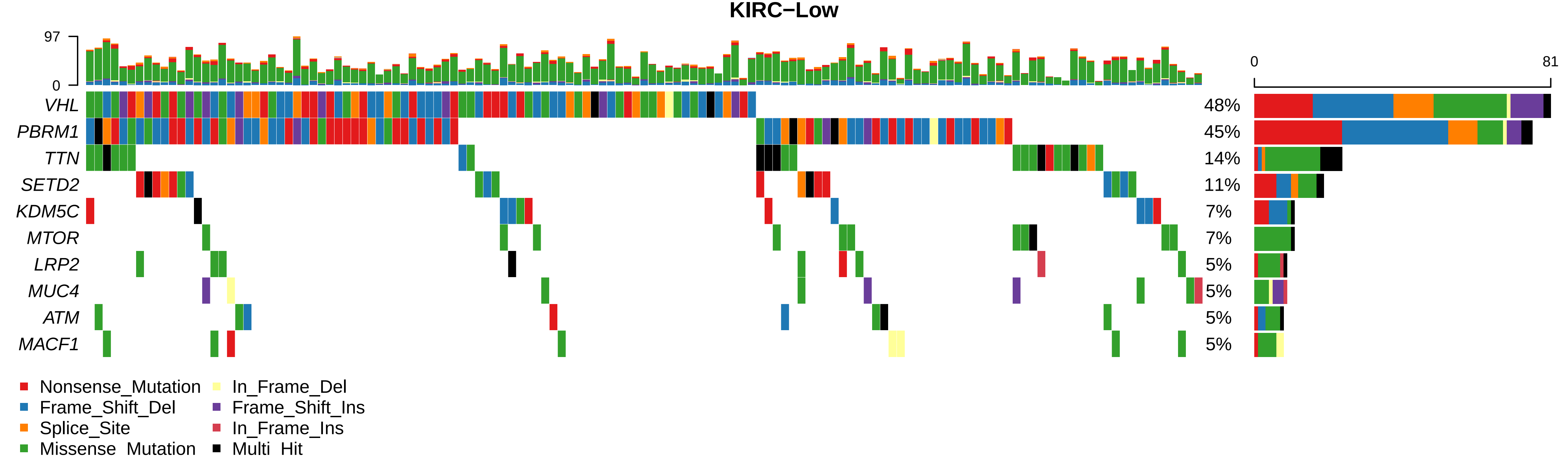
KIRC−Low
97
0
0
81
VHL
PBRM1
TTN
SETD2
KDM5C
MTOR
LRP2
MUC4
ATM
MACF1
48%
45%
14%
11%
7%
7%
5%
5%
5%
5%
Nonsense_Mutation
Frame_Shift_Del
Splice_Site
Missense_Mutation
In_Frame_Del
Frame_Shift_Ins
In_Frame_Ins
Multi_Hit

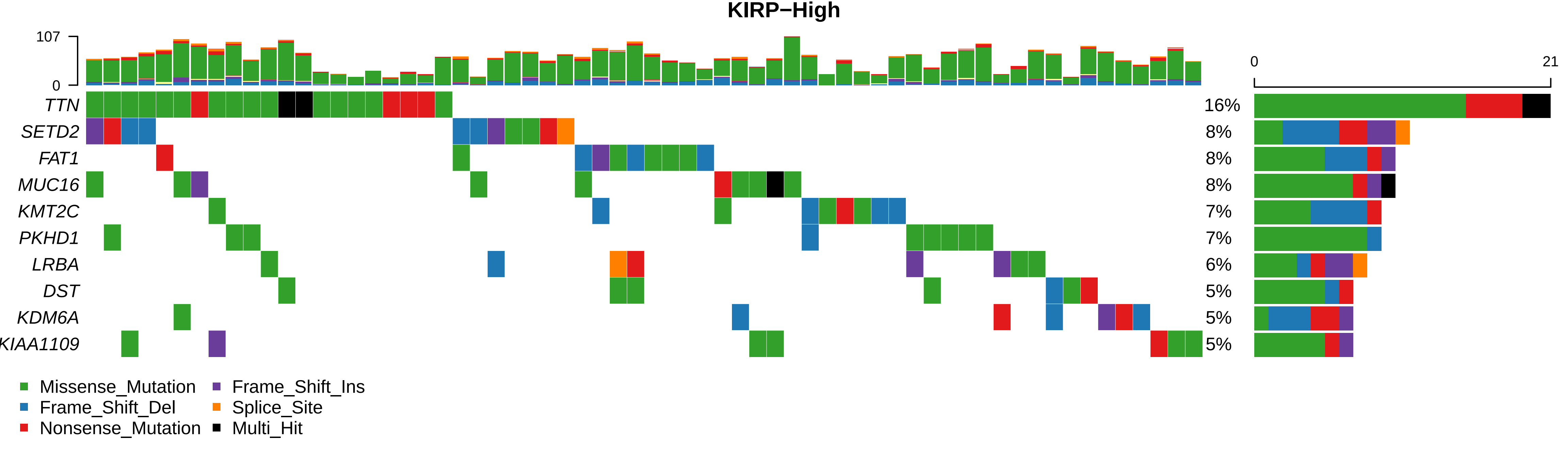
KIRP−High
107
0
0
21
TTN
SETD2
FAT1
MUC16
KMT2C
PKHD1
LRBA
DST
KDM6A
KIAA1109
16%
8%
8%
8%
7%
7%
6%
5%
5%
5%
Missense_Mutation
Frame_Shift_Del
Nonsense_Mutation
Frame_Shift_Ins
Splice_Site
Multi_Hit

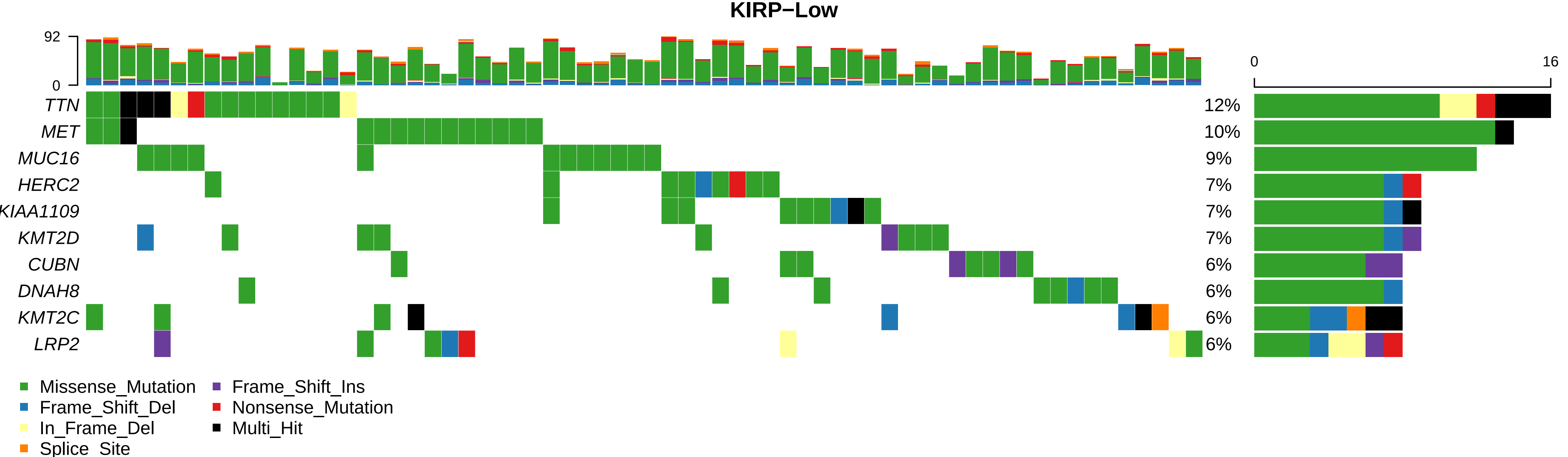
KIRP−Low
92
0
0
16
TTN
MET
MUC16
HERC2
KIAA1109
KMT2D
CUBN
DNAH8
KMT2C
LRP2
12%
10%
9%
7%
7%
7%
6%
6%
6%
6%
Missense_Mutation
Frame_Shift_Del
In_Frame_Del
Splice_Site
Frame_Shift_Ins
Nonsense_Mutation
Multi_Hit

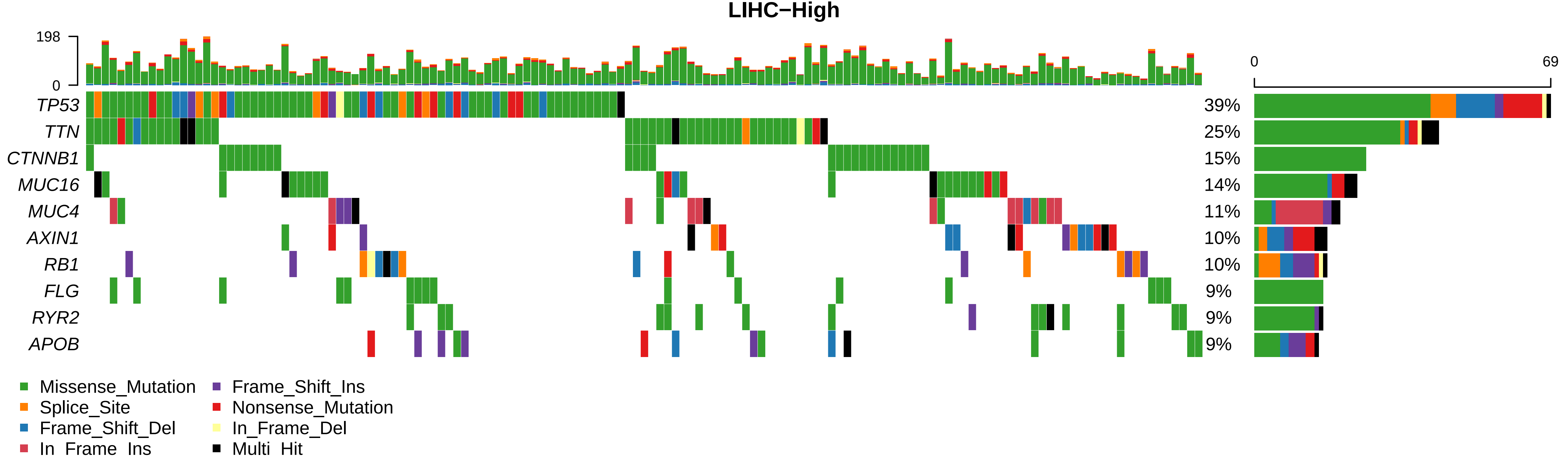
LIHC−High
198
0
TP53
TTN
CTNNB1
MUC16
MUC4
AXIN1
RB1
FLG
RYR2
APOB
39%
25%
15%
14%
11%
10%
10%
9%
9%
9%
0
69
Missense_Mutation
Splice_Site
Frame_Shift_Del
In_Frame_Ins
Frame_Shift_Ins
Nonsense_Mutation
In_Frame_Del
Multi_Hit

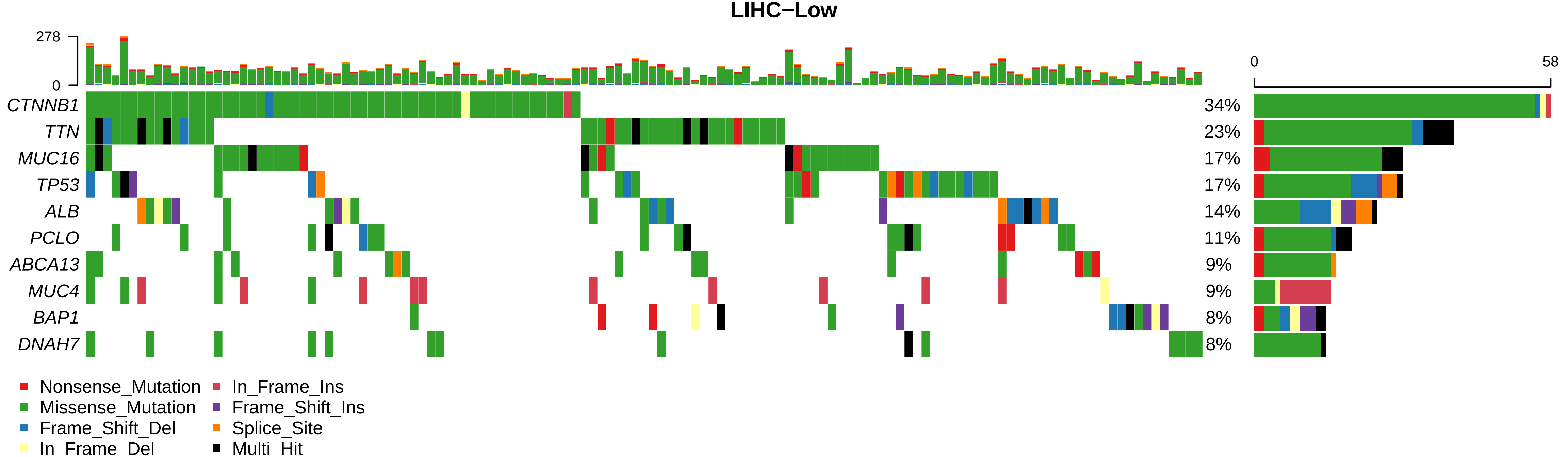
LIHC−Low
278
0
58
CTNNB1 34%
TTN 23%
MUC16 17%
TP53 17%
ALB 14%
PCLO 11%
ABCA13 9%
MUC4 9%
BAP1 8%
DNAH7 8%
Nonsense_Mutation
Missense_Mutation
Frame_Shift_Del
In_Frame_Del
In_Frame_Ins
Frame_Shift_Ins
Splice_Site
Multi_Hit

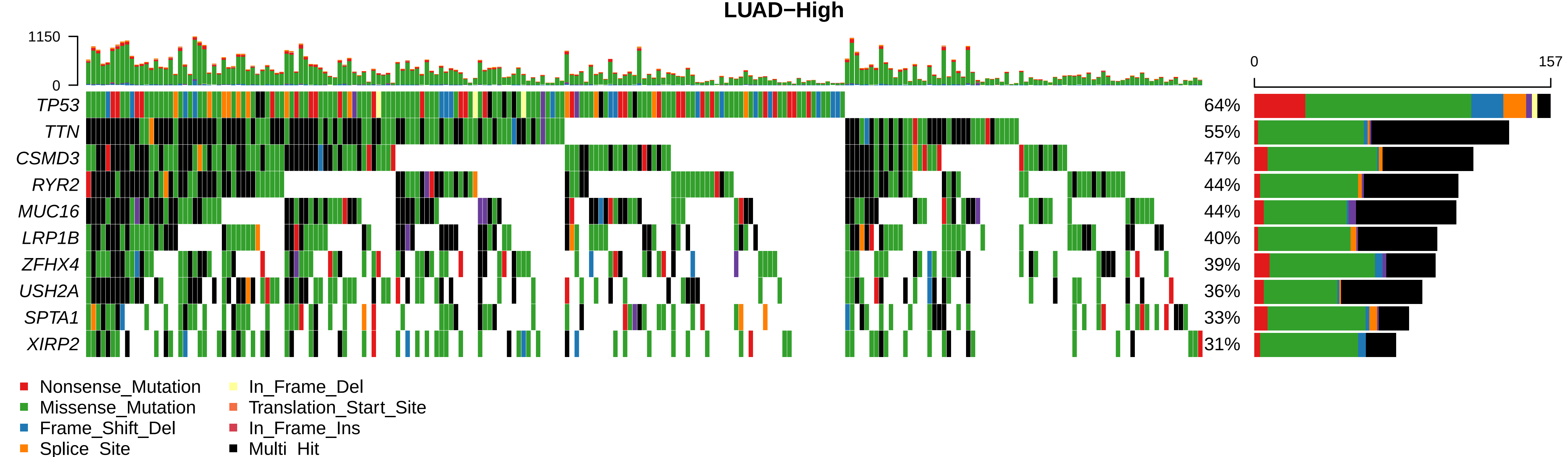
LUAD−High
1150
0
TP53
TTN
CSMD3
RYR2
MUC16
LRP1B
ZFHX4
USH2A
SPTA1
XIRP2
64%
55%
47%
44%
44%
40%
39%
36%
33%
31%
0
157
Nonsense_Mutation
Missense_Mutation
Frame_Shift_Del
Splice_Site
In_Frame_Del
Translation_Start_Site
In_Frame_Ins
Multi_Hit

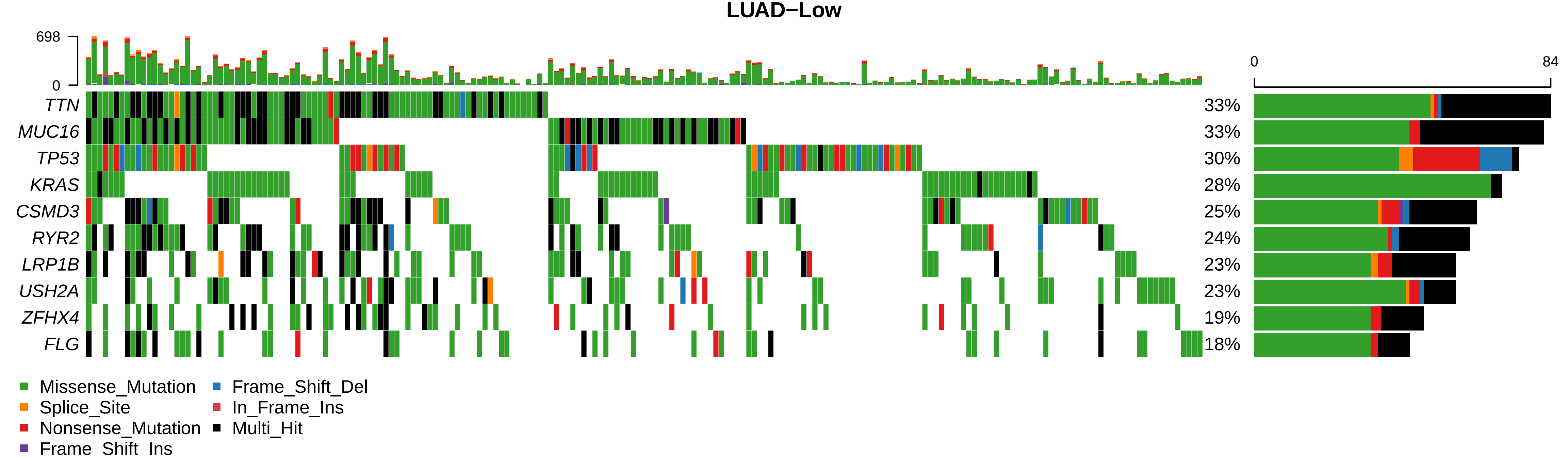

LUAD−Low
698
0
TTN 33%
MUC16 33%
TP53 30%
KRAS 28%
CSMD3 25%
RYR2 24%
LRP1B 23%
USH2A 23%
ZFHX4 19%
FLG 18%
0
84
Missense_Mutation
Splice_Site
Nonsense_Mutation
Frame_Shift_Ins
Frame_Shift_Del
In_Frame_Ins
Multi_Hit

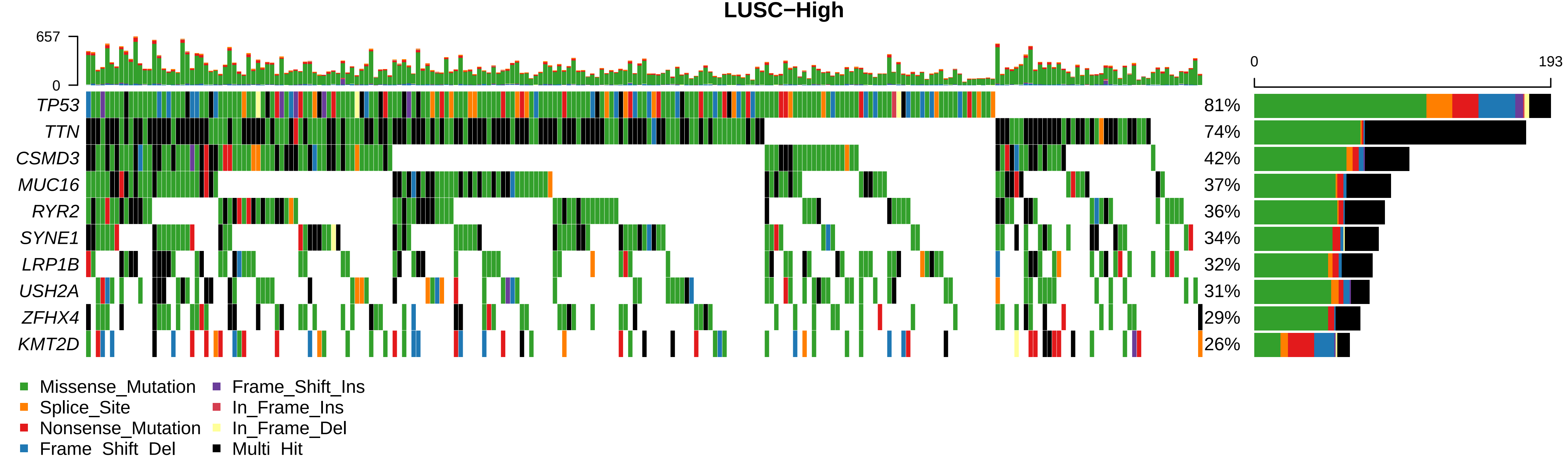
LUSC−High
657
0
TP53
TTN
CSMD3
MUC16
RYR2
SYNE1
LRP1B
USH2A
ZFHX4
KMT2D
81%
74%
42%
37%
36%
34%
32%
31%
29%
26%
0
193
Missense_Mutation
Splice_Site
Nonsense_Mutation
Frame_Shift_Del
Frame_Shift_Ins
In_Frame_Ins
In_Frame_Del
Multi_Hit

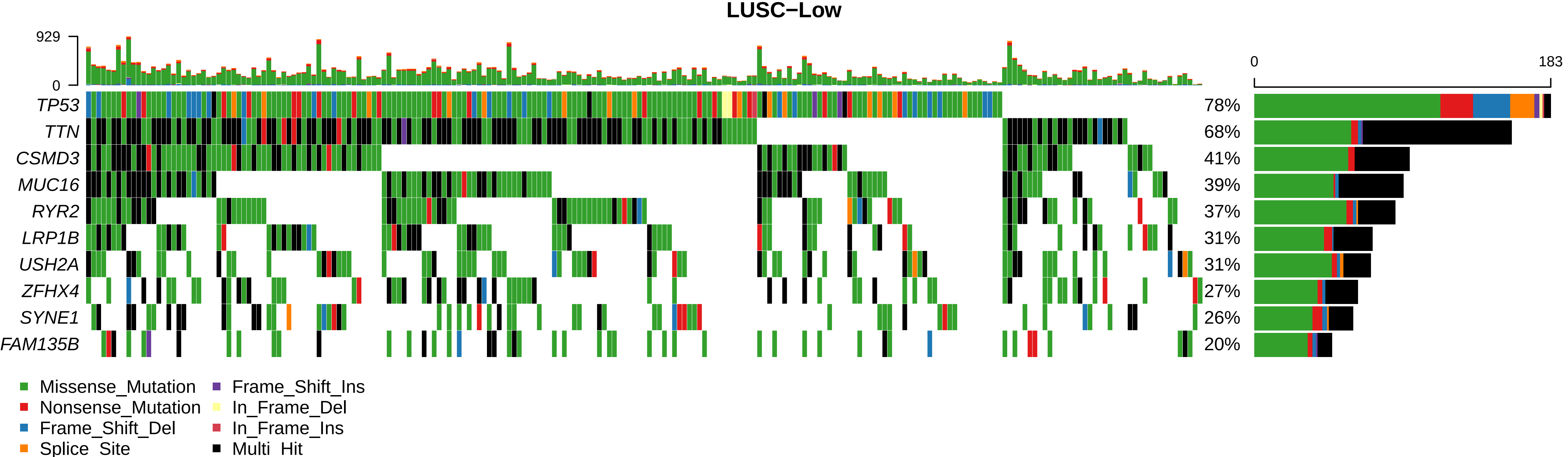
LUSC−Low
929
0
TP53
TTN
CSMD3
MUC16
RYR2
LRP1B
USH2A
ZFHX4
SYNE1
FAM135B
78%
68%
41%
39%
37%
31%
31%
27%
26%
20%
0
183
Missense_Mutation
Nonsense_Mutation
Frame_Shift_Del
Splice_Site
Frame_Shift_Ins
In_Frame_Del
In_Frame_Ins
Multi_Hit

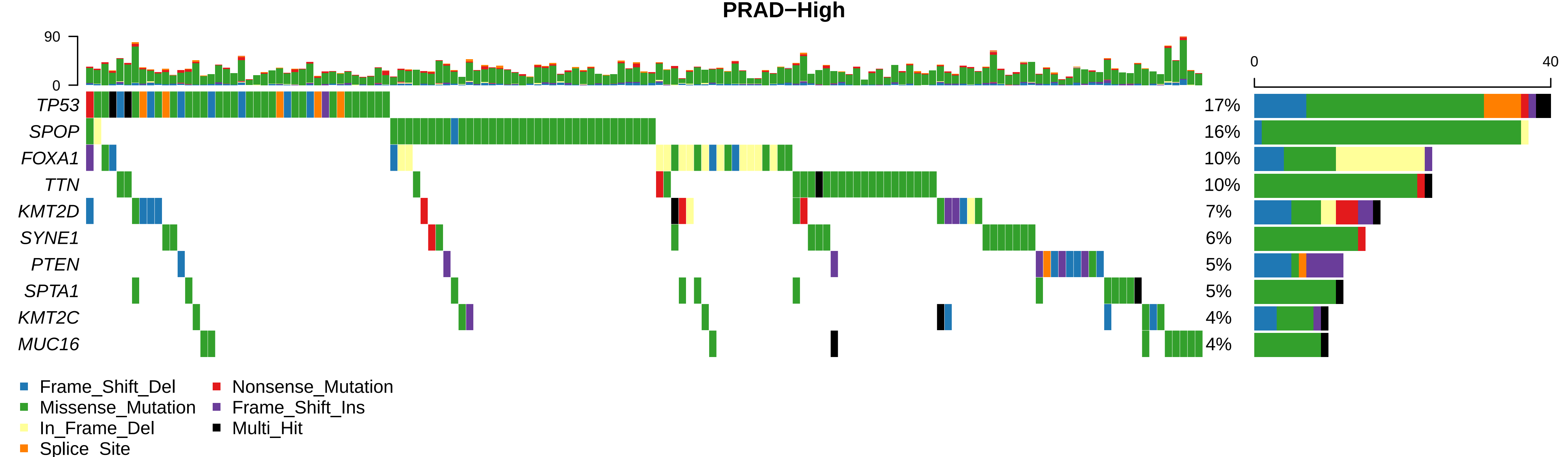
PRAD−High
90
0
TP53
SPOP
FOXA1
TTN
KMT2D
SYNE1
PTEN
SPTA1
KMT2C
MUC16
17%
16%
10%
10%
7%
6%
5%
5%
4%
4%
0
40
Frame_Shift_Del
Missense_Mutation
In_Frame_Del
Splice_Site
Nonsense_Mutation
Frame_Shift_Ins
Multi_Hit

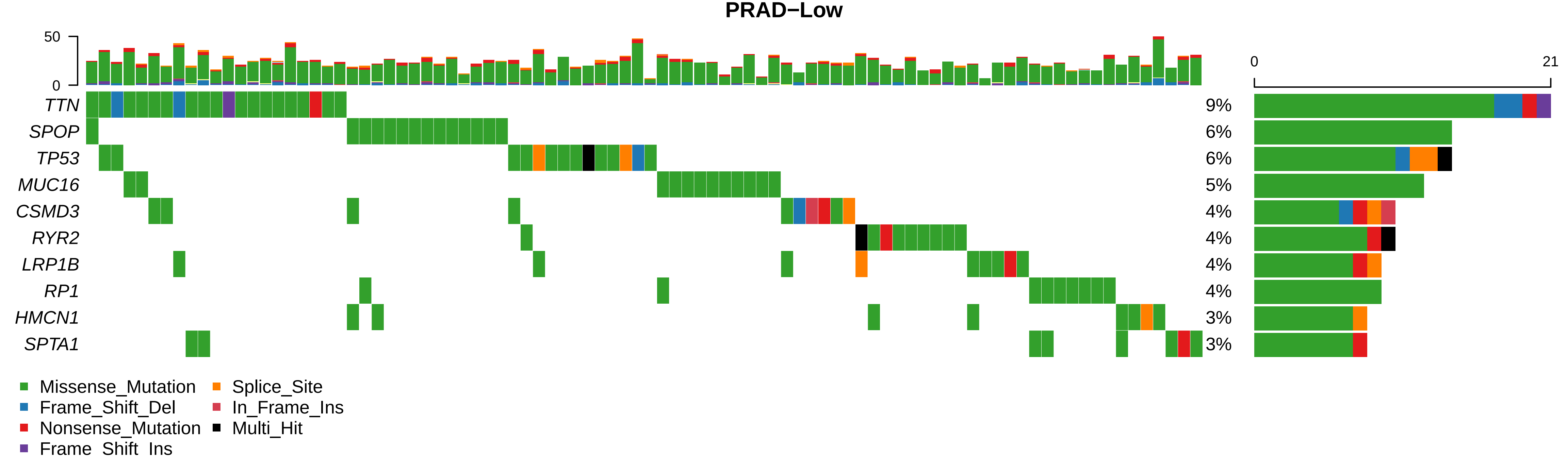
PRAD−Low
50
0
TTN
SPOP
TP53
MUC16
CSMD3
RYR2
LRP1B
RP1
HMCN1
SPTA1
9%
6%
6%
5%
4%
4%
4%
4%
3%
3%
0
21
Missense_Mutation
Frame_Shift_Del
Nonsense_Mutation
Frame_Shift_Ins
Splice_Site
In_Frame_Ins
Multi_Hit

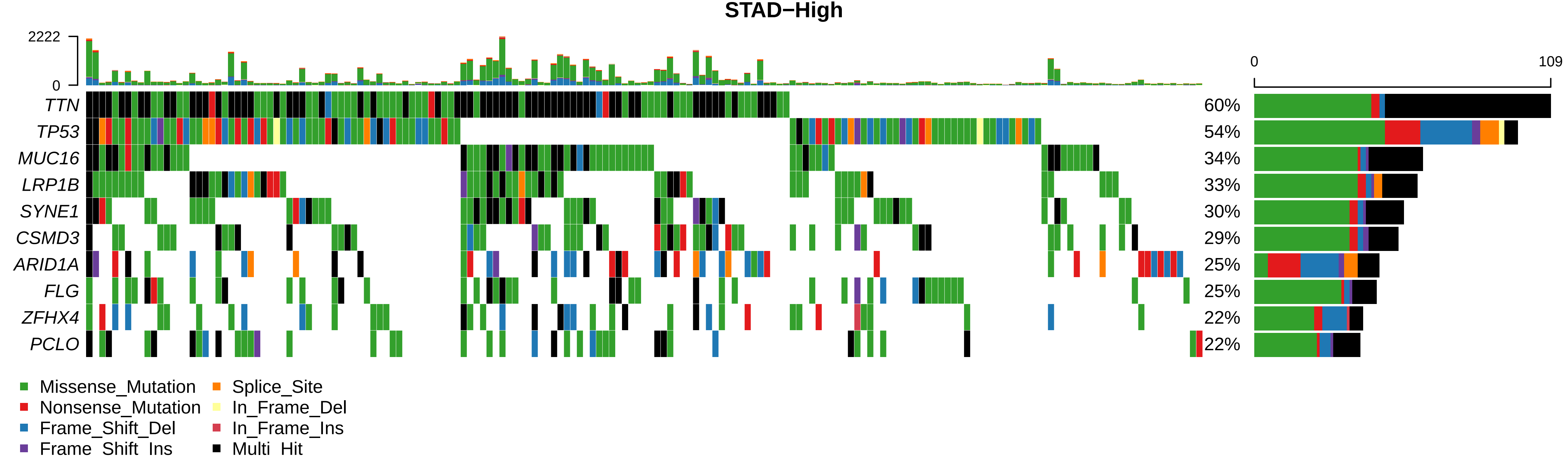
STAD−High
2222
0
0
109
TTN
TP53
MUC16
LRP1B
SYNE1
CSMD3
ARID1A
FLG
ZFHX4
PCLO
60%
54%
34%
33%
30%
29%
25%
25%
22%
22%
Missense_Mutation
Nonsense_Mutation
Frame_Shift_Del
Frame_Shift_Ins
Splice_Site
In_Frame_Del
In_Frame_Ins
Multi_Hit

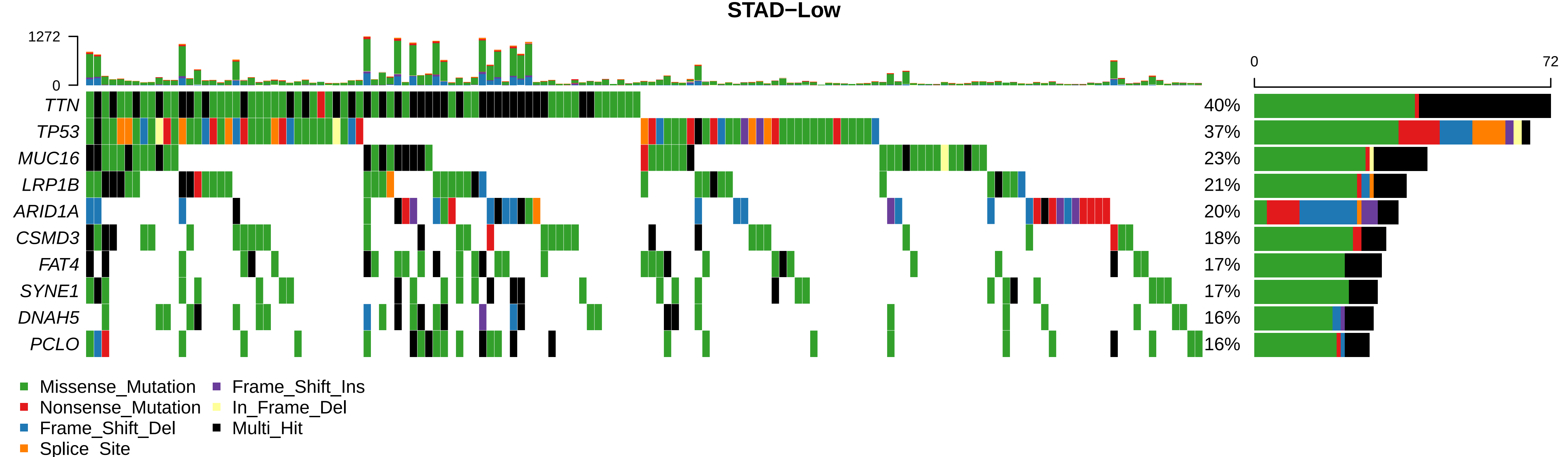
STAD−Low
1272
0
0
72
TTN
TP53
MUC16
LRP1B
ARID1A
CSMD3
FAT4
SYNE1
DNAH5
PCLO
40%
37%
23%
21%
20%
18%
17%
17%
16%
16%
Missense_Mutation
Nonsense_Mutation
Frame_Shift_Del
Splice_Site
Frame_Shift_Ins
In_Frame_Del
Multi_Hit

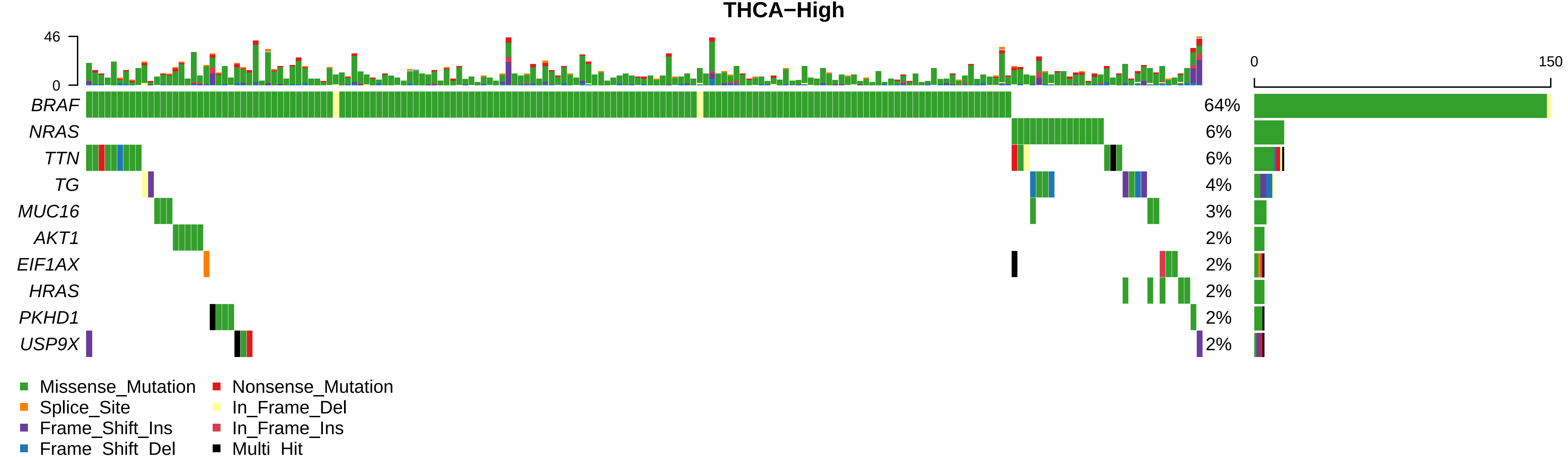
THCA−High
46
0
0
150
BRAF
NRAS
TTN
TG
MUC16
AKT1
EIF1AX
HRAS
PKHD1
USP9X
64%
6%
6%
4%
3%
2%
2%
2%
2%
2%
Missense_Mutation
Splice_Site
Frame_Shift_Ins
Frame_Shift_Del
Nonsense_Mutation
In_Frame_Del
In_Frame_Ins
Multi_Hit

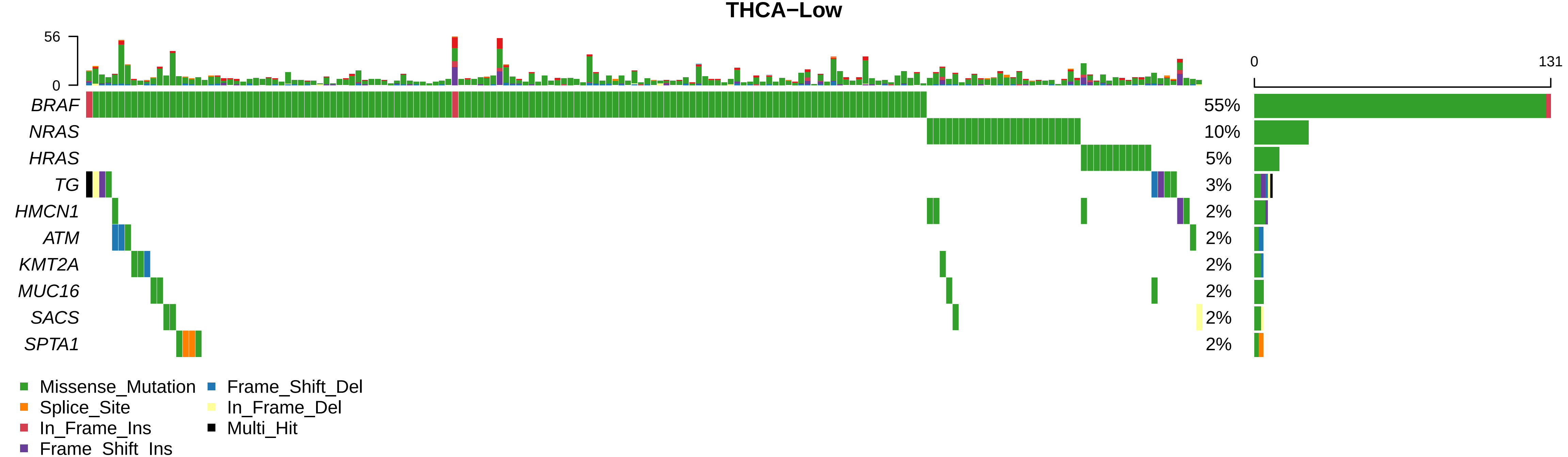
THCA−Low
56
0
0
131
BRAF
NRAS
HRAS
TG
HMCN1
ATM
KMT2A
MUC16
SACS
SPTA1
55%
10%
5%
3%
2%
2%
2%
2%
2%
2%
Missense_Mutation
Splice_Site
In_Frame_Ins
Frame_Shift_Ins
Frame_Shift_Del
In_Frame_Del
Multi_Hit
