# Supplementary material for: A score of DNA damage repair pathway with the predictive ability for chemotherapy and immunotherapy is strongly associated with immune signaling pathway in pan-cancer
Source: Front Immunol. 2022 Aug 23;13:943090. doi: 10.3389/fimmu.2022.943090 (PMC9445361; doi:10.3389/fimmu.2022.943090)
Supplement: Supplementary file 9 [file Image_9.pdf]

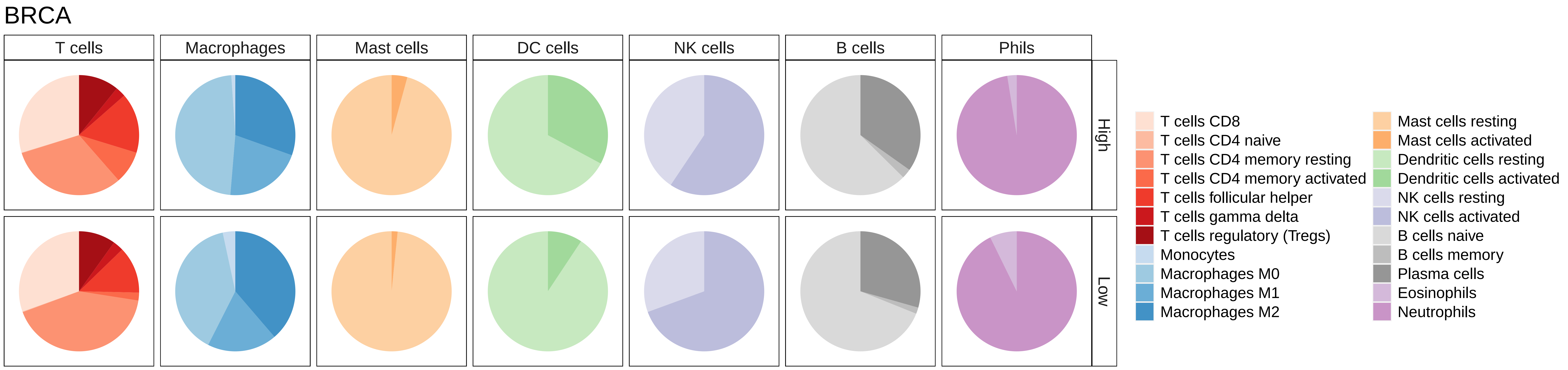
BRCA
T cells
Macrophages
Mast cells
DC cells
NK cells
B cells
Phils
High
Low
T cells CD8
T cells CD4 naive
T cells CD4 memory resting
T cells CD4 memory activated
T cells follicular helper
T cells gamma delta
T cells regulatory (Tregs)
Monocytes
Macrophages M0
Macrophages M1
Macrophages M2
Mast cells resting
Mast cells activated
Dendritic cells resting
Dendritic cells activated
NK cells resting
NK cells activated
B cells naive
B cells memory
Plasma cells
Eosinophils
Neutrophils

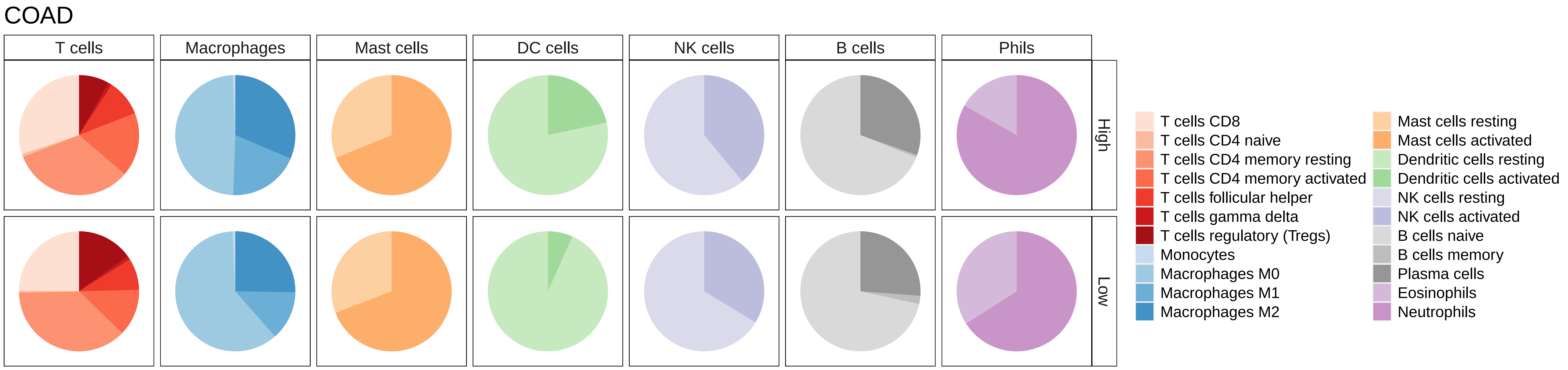
COAD
T cells
Macrophages
Mast cells
DC cells
NK cells
B cells
Phils
High
Low
T cells CD8
T cells CD4 naive
T cells CD4 memory resting
T cells CD4 memory activated
T cells follicular helper
T cells gamma delta
T cells regulatory (Tregs)
Monocytes
Macrophages M0
Macrophages M1
Macrophages M2
Mast cells resting
Mast cells activated
Dendritic cells resting
Dendritic cells activated
NK cells resting
NK cells activated
B cells naive
B cells memory
Plasma cells
Eosinophils
Neutrophils

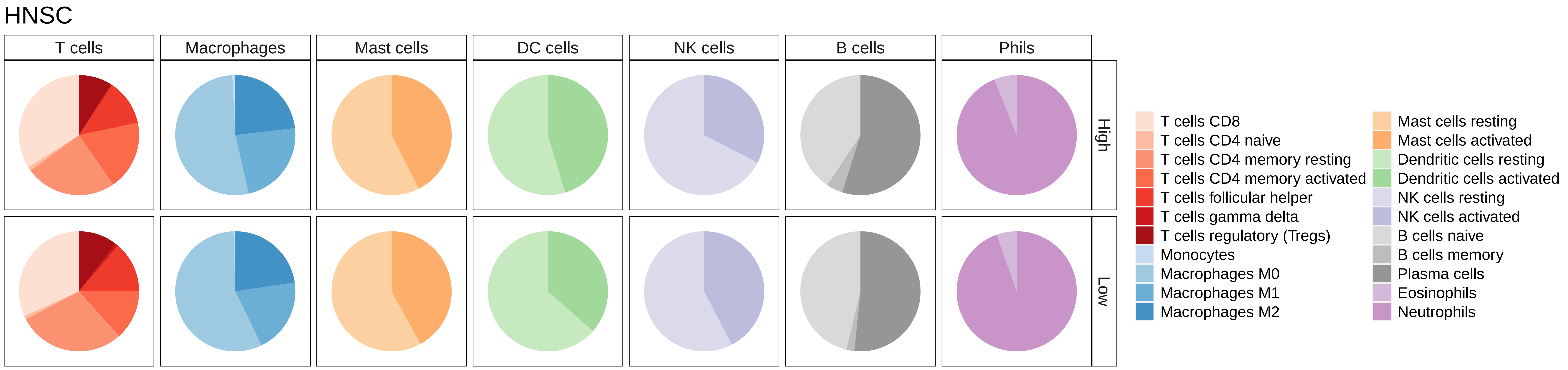
HNSC
T cells
Macrophages
Mast cells
DC cells
NK cells
B cells
Phils
High
Low
T cells CD8
T cells CD4 naive
T cells CD4 memory resting
T cells CD4 memory activated
T cells follicular helper
T cells gamma delta
T cells regulatory (Tregs)
Monocytes
Macrophages M0
Macrophages M1
Macrophages M2
Mast cells resting
Mast cells activated
Dendritic cells resting
Dendritic cells activated
NK cells resting
NK cells activated
B cells naive
B cells memory
Plasma cells
Eosinophils
Neutrophils

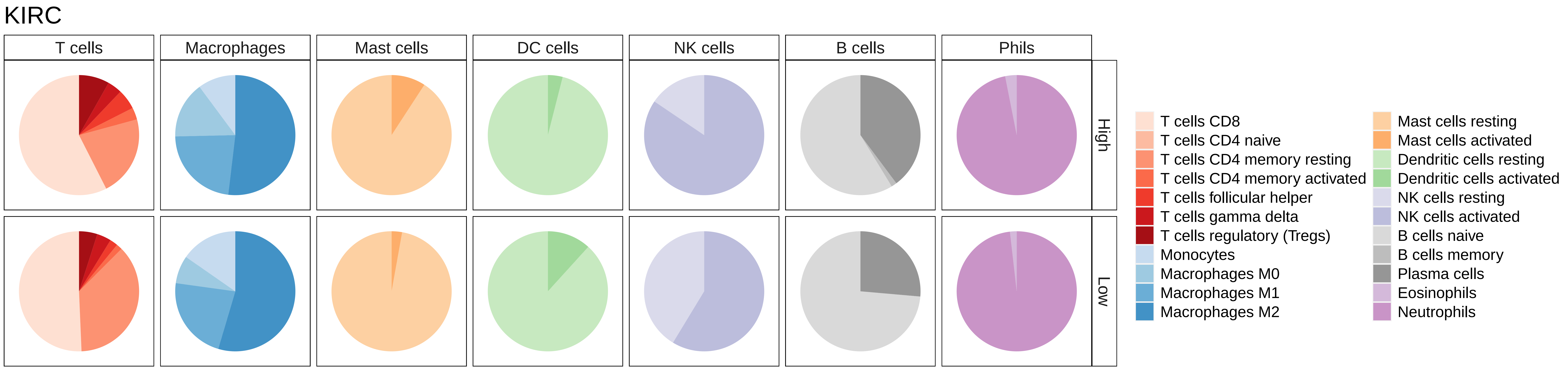
KIRC
T cells
Macrophages
Mast cells
DC cells
NK cells
B cells
Phils
High
Low
T cells CD8
T cells CD4 naive
T cells CD4 memory resting
T cells CD4 memory activated
T cells follicular helper
T cells gamma delta
T cells regulatory (Tregs)
Monocytes
Macrophages M0
Macrophages M1
Macrophages M2
Mast cells resting
Mast cells activated
Dendritic cells resting
Dendritic cells activated
NK cells resting
NK cells activated
B cells naive
B cells memory
Plasma cells
Eosinophils
Neutrophils

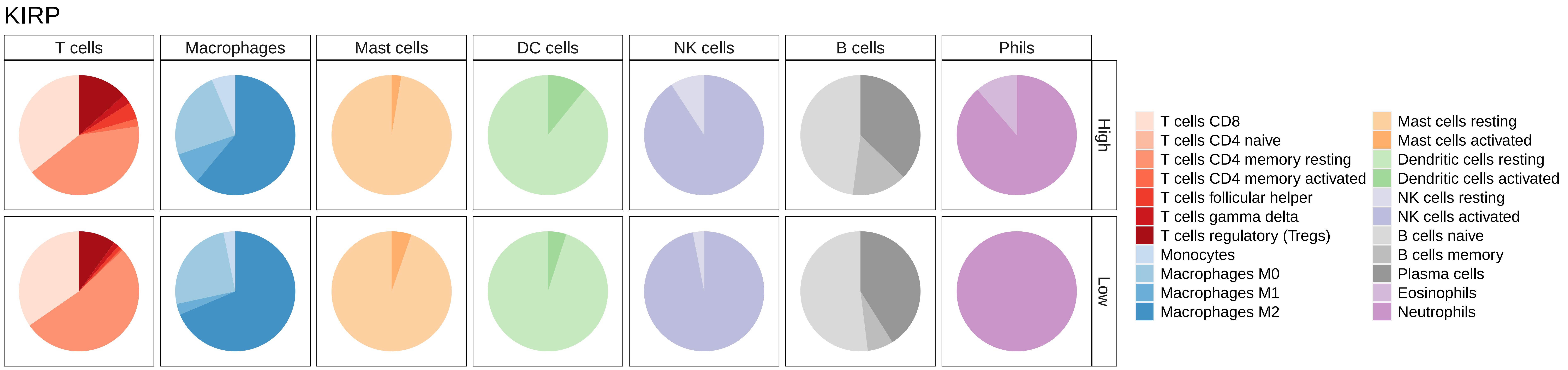

KIRP
T cells
Macrophages
Mast cells
DC cells
NK cells
B cells
Phils
High
Low
T cells CD8
T cells CD4 naive
T cells CD4 memory resting
T cells CD4 memory activated
T cells follicular helper
T cells gamma delta
T cells regulatory (Tregs)
Monocytes
Macrophages M0
Macrophages M1
Macrophages M2
Mast cells resting
Mast cells activated
Dendritic cells resting
Dendritic cells activated
NK cells resting
NK cells activated
B cells naive
B cells memory
Plasma cells
Eosinophils
Neutrophils

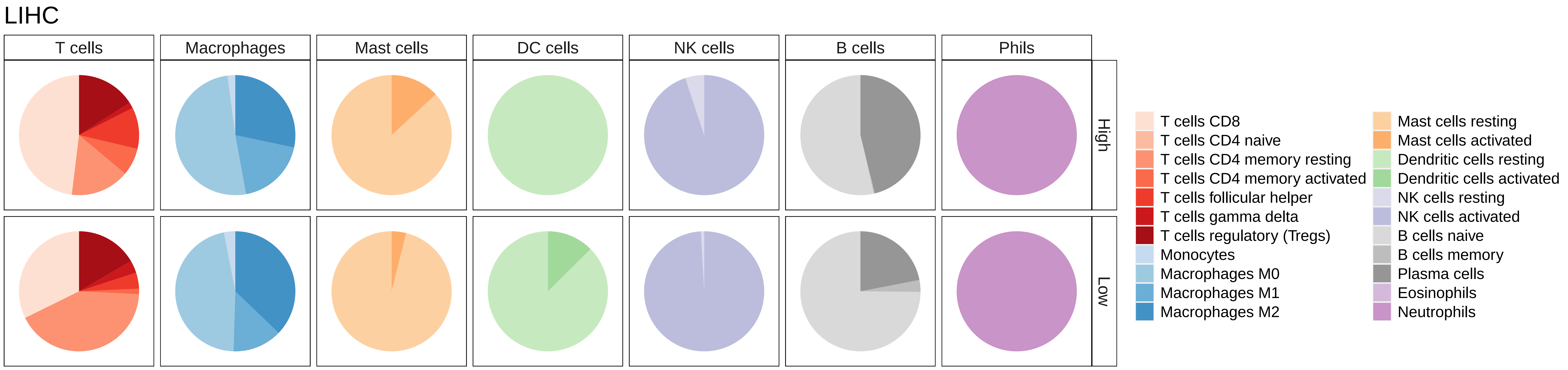

LIHC
T cells
Macrophages
Mast cells
DC cells
NK cells
B cells
Phils
High
Low
T cells CD8
T cells CD4 naive
T cells CD4 memory resting
T cells CD4 memory activated
T cells follicular helper
T cells gamma delta
T cells regulatory (Tregs)
Monocytes
Macrophages M0
Macrophages M1
Macrophages M2
Mast cells resting
Mast cells activated
Dendritic cells resting
Dendritic cells activated
NK cells resting
NK cells activated
B cells naive
B cells memory
Plasma cells
Eosinophils
Neutrophils

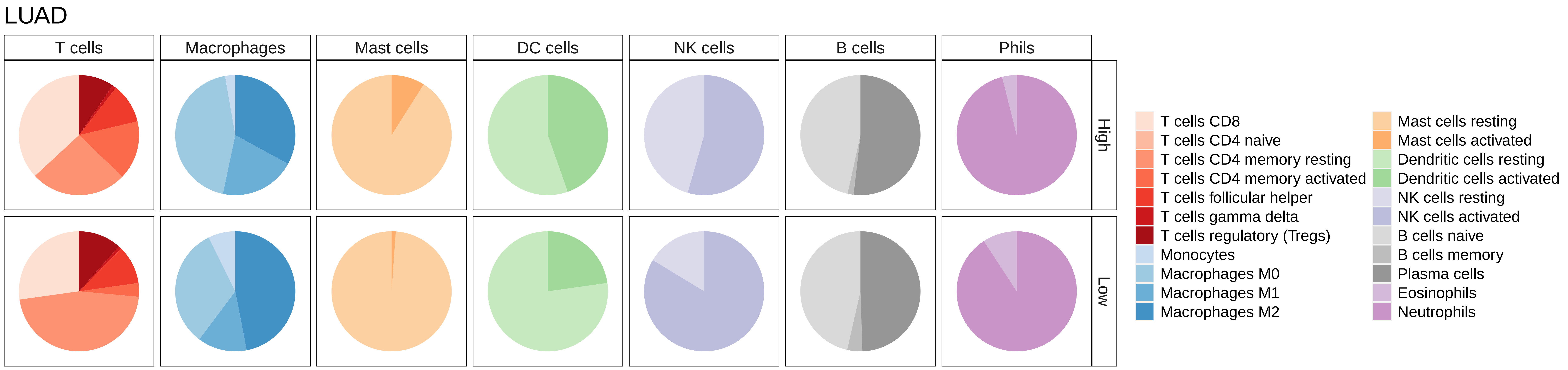

LUAD
T cells
Macrophages
Mast cells
DC cells
NK cells
B cells
Phils
High
Low
T cells CD8
T cells CD4 naive
T cells CD4 memory resting
T cells CD4 memory activated
T cells follicular helper
T cells gamma delta
T cells regulatory (Tregs)
Monocytes
Macrophages M0
Macrophages M1
Macrophages M2
Mast cells resting
Mast cells activated
Dendritic cells resting
Dendritic cells activated
NK cells resting
NK cells activated
B cells naive
B cells memory
Plasma cells
Eosinophils
Neutrophils

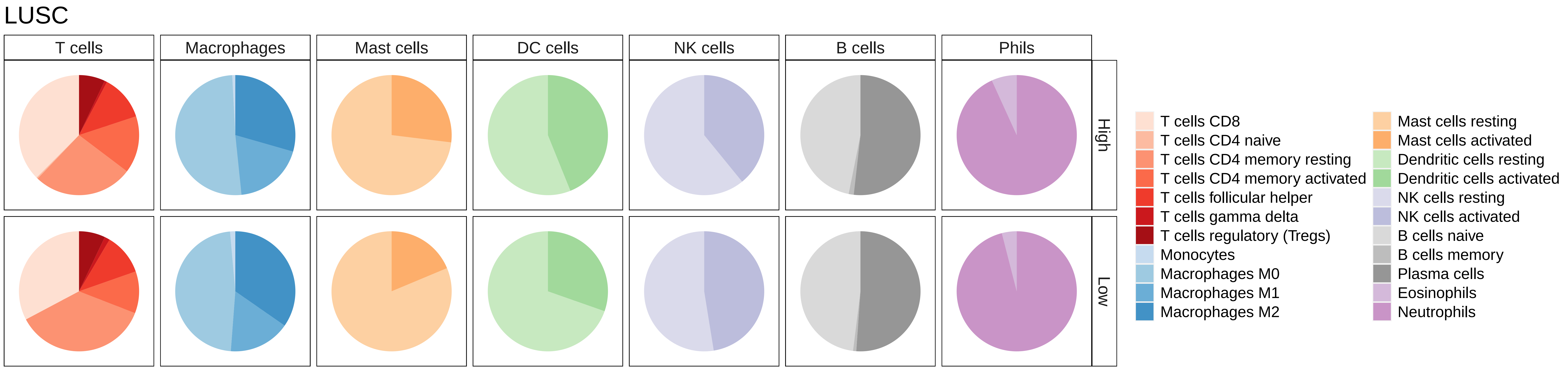
LUSC
T cells
Macrophages
Mast cells
DC cells
NK cells
B cells
Phils
High
Low
T cells CD8
T cells CD4 naive
T cells CD4 memory resting
T cells CD4 memory activated
T cells follicular helper
T cells gamma delta
T cells regulatory (Tregs)
Monocytes
Macrophages M0
Macrophages M1
Macrophages M2
Mast cells resting
Mast cells activated
Dendritic cells resting
Dendritic cells activated
NK cells resting
NK cells activated
B cells naive
B cells memory
Plasma cells
Eosinophils
Neutrophils

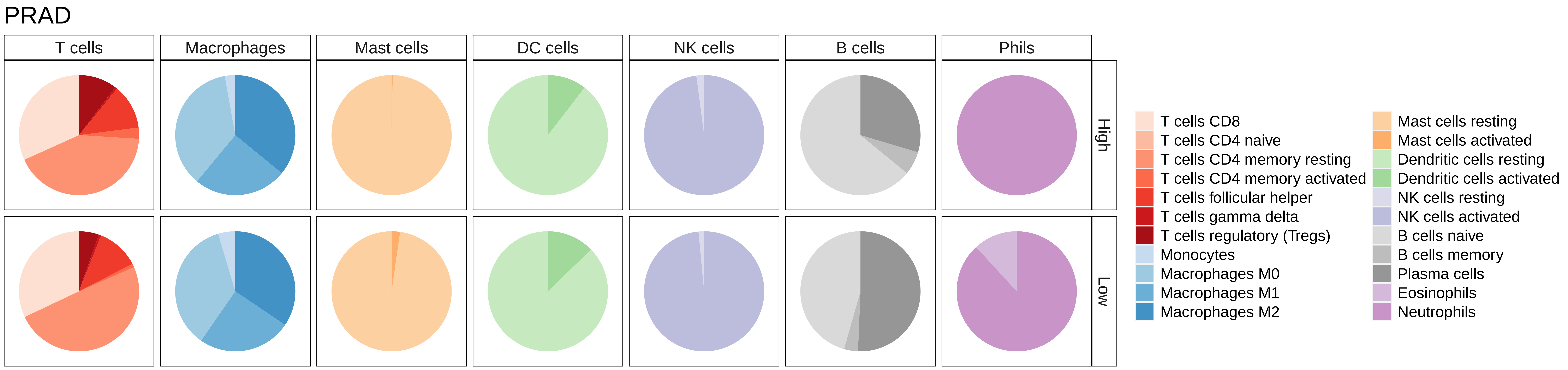
PRAD
T cells
Macrophages
Mast cells
DC cells
NK cells
B cells
Phils
High
Low
T cells CD8
T cells CD4 naive
T cells CD4 memory resting
T cells CD4 memory activated
T cells follicular helper
T cells gamma delta
T cells regulatory (Tregs)
Monocytes
Macrophages M0
Macrophages M1
Macrophages M2
Mast cells resting
Mast cells activated
Dendritic cells resting
Dendritic cells activated
NK cells resting
NK cells activated
B cells naive
B cells memory
Plasma cells
Eosinophils
Neutrophils

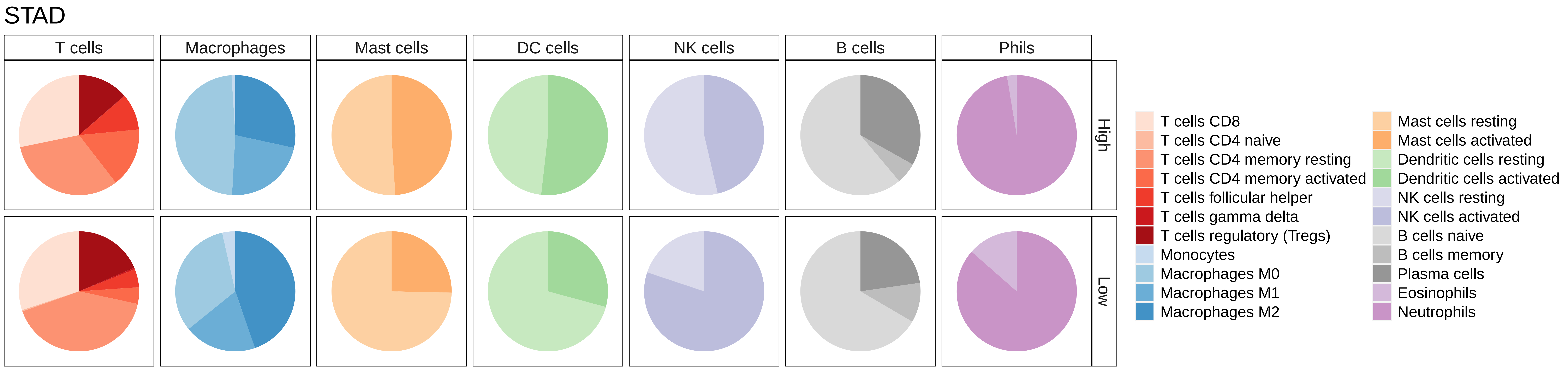

STAD
T cells
Macrophages
Mast cells
DC cells
NK cells
B cells
Phils
High
Low
T cells CD8
T cells CD4 naive
T cells CD4 memory resting
T cells CD4 memory activated
T cells follicular helper
T cells gamma delta
T cells regulatory (Tregs)
Monocytes
Macrophages M0
Macrophages M1
Macrophages M2
Mast cells resting
Mast cells activated
Dendritic cells resting
Dendritic cells activated
NK cells resting
NK cells activated
B cells naive
B cells memory
Plasma cells
Eosinophils
Neutrophils

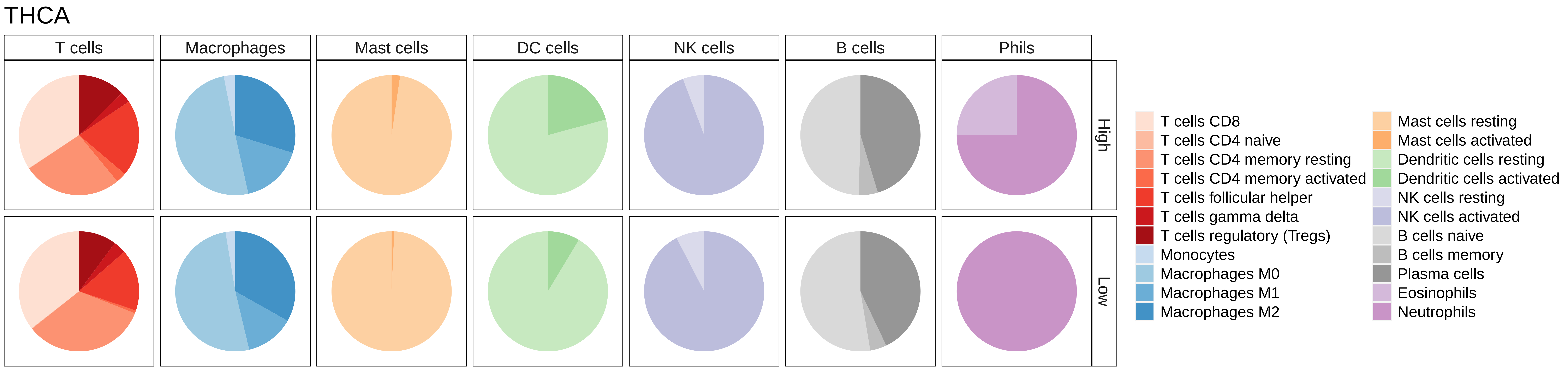

THCA
T cells
Macrophages
Mast cells
DC cells
NK cells
B cells
Phils
High
Low
T cells CD8
T cells CD4 naive
T cells CD4 memory resting
T cells CD4 memory activated
T cells follicular helper
T cells gamma delta
T cells regulatory (Tregs)
Monocytes
Macrophages M0
Macrophages M1
Macrophages M2
Mast cells resting
Mast cells activated
Dendritic cells resting
Dendritic cells activated
NK cells resting
NK cells activated
B cells naive
B cells memory
Plasma cells
Eosinophils
Neutrophils
